# Medicinal ethnobotany of wild plants: a cross-cultural comparison around Georgia-Turkey border, the Western Lesser Caucasus

**DOI:** 10.1186/s13002-020-00415-y

**Published:** 2020-11-23

**Authors:** Ceren Kazancı, Soner Oruç, Marine Mosulishvili

**Affiliations:** 1 School of Natural Sciences and Medicine, Ilia State University, Cholokashvili 3/5 Avenue, 0162 Tbilisi, Georgia; 2 Faculty of Forestry, Forest Botany, Artvin Çoruh University, Seyitler Yerleşkesi, 08100 Artvin, Turkey

**Keywords:** Medicinal ethnobotany, The Caucasus, Medicinal plants, Transhumant people, Cross cultural research, Border ethnobotany, Georgia, Turkey, Biocultural conservation

## Abstract

**Background:**

The Mountains of the Western Lesser Caucasus with its rich plant diversity, multicultural and multilingual nature host diverse ethnobotanical knowledge related to medicinal plants. However, cross-cultural medicinal ethnobotany and patterns of plant knowledge have not yet been investigated in the region. Doing so could highlight the salient medicinal plant species and show the variations between communities. This study aimed to determine and discuss the similarities and differences of medicinal ethnobotany among people living in highland pastures on both sides of the Georgia-Turkey border.

**Methods:**

During the 2017 and 2018 summer transhumance period, 119 participants (74 in Turkey, 45 in Georgia) were interviewed with semi-structured questions. The data was structured in use-reports (URs) following the ICPC classification. Cultural Importance (CI) Index, informant consensus factor (FIC), shared/separate species-use combinations, as well as literature data were used for comparing medicinal ethnobotany of the communities.

**Results:**

One thousand five hundred six UR for 152 native wild plant species were documented. More than half of the species are in common on both sides of the border. Out of 817 species-use combinations, only 9% of the use incidences are shared between communities across the border. Around 66% of these reports had not been previously mentioned specifically in the compared literature. Only 33 species have similar use reports in both countries, most important of which are *Plantago major*, *Urtica dioica*, *Picea orientalis*, *Anthemis* spp., *Sambucus ebulus*, *Achillea millefolium*, *Helichrysum rubicundum*, *Mentha longifolia*, *Pinus sylvestris* var. *hamata*, *Hypericum perforatum*, *Tussilago farfara*, *Helichrysum plicatum*, *Rumex crispus*, *Berberis vulgaris*, and *Origanum vulgare*. More than half of species reported in each country were found to have more than one part of the plant valued for medicinal use. The most common way of using plants medicinally in both countries is drinking the water infusion of aerial parts with flowers. Based on CI index value, two-thirds of the salient 15 genera in both countries have use reports in at least seven medicinal use categories. While the most cited category with highest FIC is digestive in Georgia, it is skin category in Turkey. Patterns of medicinal plant knowledge among studied communities appear to be connected with more than one cultural factor, in particular ethnolinguistic diversity, cultural background, and access to multilingual written folk and scientific literature, or probably a combination of various factors.

**Conclusion:**

Considering the regions’ floral similarity, common historical-cultural contact, and similar livelihood strategies of the communities, shared ethnomedicinal knowledge across the Georgia-Turkey border is quite low. Even though the impacts of accessing multilingual folk and scientific literature are likely to be significant, the factors that shape the medicinal plant knowledge patterns of the communities are shown to be variable among species, needing further research into intracultural diversity and socio-economical conditions, as well as the political history across the border.

## Introduction

The mountains of the Western Lesser Caucasus are part of the Caucasus Hotspot, one of the 36 global biodiversity hotspots of the world [1–3]. This hotspot harbors around 7000 vascular plant species, around 25% of which are endemic to the region [4]. Moreover, it is known to be a home to high linguistic, genetic, and ethnic diversity [5]. Indeed, various travelers and researchers have been impressed by the diversity of language and ethnicity of the region, calling here “the mountain of tongues” [6]. Similarly, several researchers highlight the significance of mountainous regions worldwide not only for biodiversity but also for biocultural diversity [7–9], “the diversity of life in all its manifestations: biological, cultural, and linguistic” [10]. Despite this, the lack of information on plant resources in ethnographic studies in particular has been identified [9], as well the lack of studies on the relationship between mountains, biodiversity, and cultural diversity [7]. More effort to document and protect traditional knowledge and practices in mountainous areas has been called for, to sustain continued social-ecological health and wellbeing of humanity [9].

Recent studies conducted in Georgia (Sakartvelo) reveal a noteworthy ethnobotanical knowledge of people living in various regions [11–17]. Furthermore, the book “Ethnobotany of the Caucasus” presents detailed information on about culturally salient 130 plant species currently and historically noted in the South Caucasus (Georgia, Armenia and Azerbaijan) [18]. However, apart from a number of ethnobotanical studies published in certain parts of the Western Lesser Caucasus region, little ethnobotanical knowledge has yet been systematically documented in the mountains around Turkey-Georgia border. For instance in Turkey, two general surveys conducted in some parts of Artvin province report the medicinal ethnobotany of 20 plant species [19, 20]. A recent survey conducted in a national park in Artvin also states medicinal knowledge of 37 plant species [21]. The neighboring province of Ardahan is another ethnobotanically least studied area of East Anatolia [22]. Folk knowledge of 18 medicinal plant species were reported around the Göle and Çıldır districts of Ardahan [20], while the ethnobotanical knowledge of 65 plant species were reported from the Çıldır district [23]. On the Georgian side, medicinal folk knowledge of at least 27 rare and endangered medicinal plants were recorded for the Samtskhe-Javakheti region [24]. In addition, around 40 medicinal plant species were listed in a systematic ethnobotanical study in Samtskhe-Javakheti region [14]. A study of folk usage of medicinal plants in Adjara reported knowledge relating to 194 plant taxa [25].

Recent studies conducted in Europe highlight the significance of cross-cultural and cross-border ethnobotanical research to fully understand the factors that shape plant knowledge and uses by communities living closely under similar environmental conditions [26–28]. Differences in cultural backgrounds (e.g., ethnicity, language, medicinal belief systems, religion) are proposed to be significant factors affecting varying concepts of medicinal knowledge within such communities in Europe [29–32]. Moreover, the influence of written literature on the current medicinal plant knowledge and usage has been highlighted in detail [30, 31] and the influence of USSR Pharmacopedia, especially for post-Soviet countries is discussed [27]. Similarly, the impact of “official” sources from Soviet times on traditional ethnomedicinal knowledge was investigated in a study in Armenia, which identified a “new tradition,” which they suggest has indirectly promoted the enrichment and preservation of phytomedicinal knowledge and traditions [33].

Given the above situation, the objectives of this study are:

- To document the medicinal folk knowledge about wild plants and highlight shared and divergent knowledge of use between transhumant communities around Georgia-Turkey border, as well as comparing the knowledge with the ethnomedicinal literature sources.
- To evaluate the cultural significance of the most salient plant families, genera, species, and their medicinal uses among participants in Georgia and in Turkey. Furthermore, the underlying factors of use/knowledge patterns for these plant species will be discussed.

## Material and methods

### Area of study

**Fig. 1:**
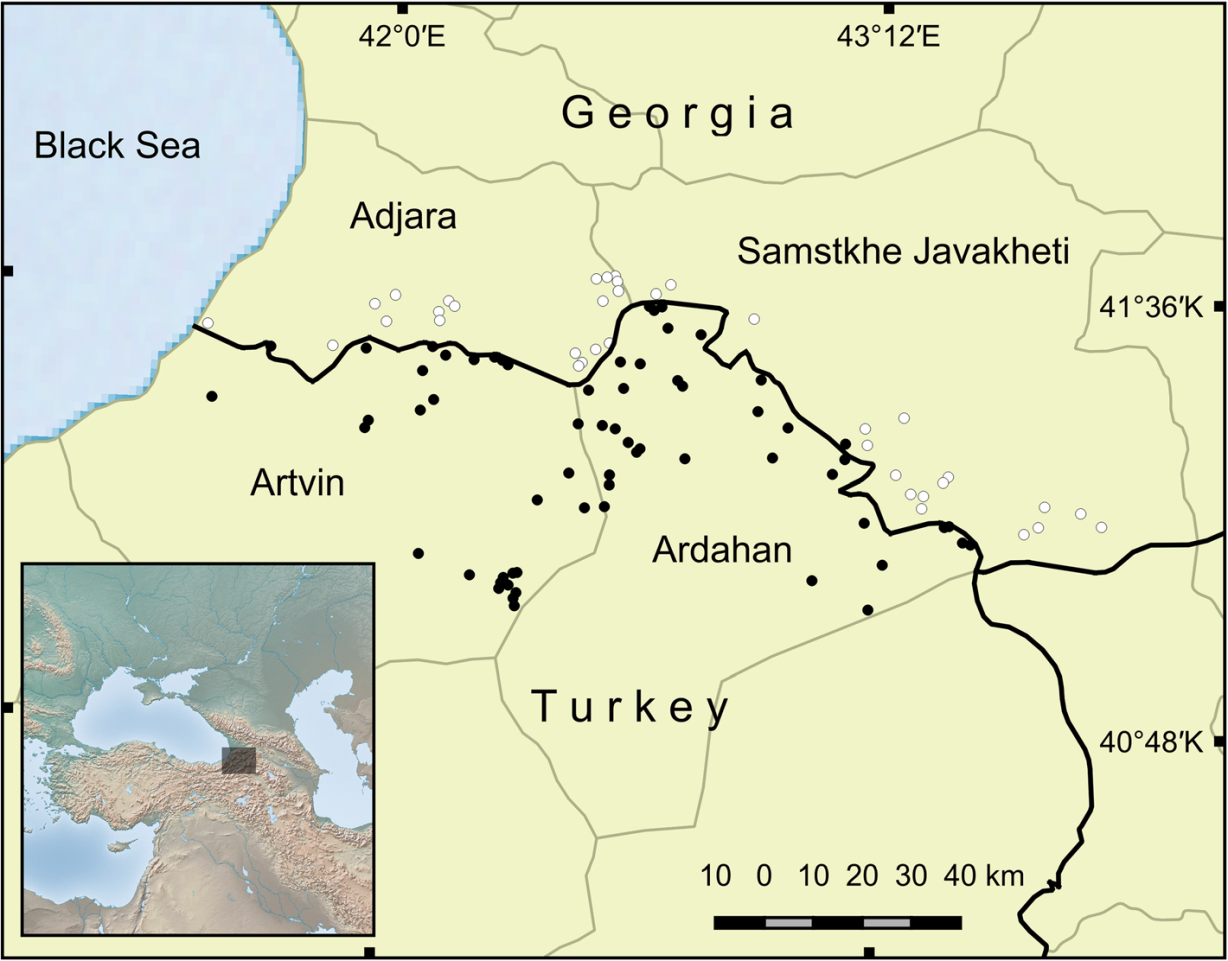
Map of the study area: Distribution of visited highland pastures and villages. Black dots correspond to places in Turkey; white dots refer to places in Georgia

The geographical area covered in this study is located along the border between Georgia and Turkey, in the Western Lesser Caucasus (Fig. 1). This corresponds to part of the highlands between the Hopa-Artvin-Ardahan-Çıldır main road (41° 23′ 38′′ N, 41° 25′ 08′′′ E–41° 01′33′′ N, 43° 28′14′′ E) in Turkey and Batumi-Khulo-Akhaltsikhe-Ninotsminda main road (41° 36′03′′ N, 41° 34′30′′ E–41° 08′ 30′′ N, 43° 47′ 24′′ E) in Georgia. It falls within the borders of Adjara and Samtskhe-Javakheti regions in Georgia; Artvin and Ardahan provinces in Turkey. The area includes the characteristics of three of the world’s ecological regions: The Caucasus Mixed Forest Ecoregion, Euxine Colchic Deciduous Forest Ecoregion, and to a lesser extent Eastern Anatolian Montane Steppe Ecoregion [34]. Its principal climates range from humid subtropical and mildly dry subtropical mountainous to continental climates. Dominant natural landscapes extend from forest and high mountain vegetation to Caucasian sub-alpine meadows and steppe meadows with freshwater lakes, mainly located along the Ardahan and the Samsthke-Javakheti border [35].

Between 1300 BCE and 580 ADE, the area fell within the kingdoms of Colchis, Diauehi, and Iberia. The region witnessed various wars, migrations, and deportations and several kingdoms, empires, principalities, and countries succeeded until the current day. The variety of ethnolinguistic groups inhabiting the area include Turks, Georgians, Armenians, Kurds, Azeris, Laz people, Hemshins, and Russians, with small-scale agriculture and relatively large-scale livestock farming as the main economic activities. Nearly all participants in this study were transhumant, maintaining an agro-pastoral way of life. Highland pastures, referred to as “*yayla*” in Turkey, are known as “*mta*” and/or “ialagi” (iala) in Georgia. People move to their summer pastures at the end of May, where for 3 to 5 months they live mainly in wooden houses, some living in dry stone dwellings or even in tents.

### Ethnobotanical data collection

To restrict the focus of the study on the ethnobotanical knowledge of transhumant people, more than two-thirds of the fieldwork was conducted in highland pastures along the Georgian-Turkish border. Firstly, over 150 potential highland pastures were identified between altitudes of 1600 m and 2500 m within the study area, using Google Earth. Later, possible research locations were selected from among those settlements according to a number of geographical barriers (mountains, rivers, lakes, passes) that would help identify high diversity of floral and cultural characters. By attempting to reach people who had maintained their agro-pastoral transhumance lifestyle, it was expected that these people would have been in contact with a variety of vegetation types during regular seasonal migrations, thus having a relatively strong living memory of traditional knowledge and practices related to wild plants.

In the summer of 2016, 2 weeks of non-systematic preliminary fieldwork were conducted, with the undertaking of informal interviews in 20 highland pastures and villages in Georgia and Turkey [36]. Over the following two summers (2017–2018), a total of around 90 days of systematic fieldwork were carried out during the period of transhumance (approximately June 15–September 15), visiting 102 highland pastures, 65 in Turkey and 37 in Georgia (Fig. 1). During that time, 119 participants were interviewed, 74 in Turkey (41 female; 33 male), and 45 in Georgia (28 female; 17 male). The mean ages of participants were 57 and 58 respectively in Georgia and in Turkey.

Official research and plant collection permits were obtained from the Ministry of Forest and Water Affairs (Issue date-no: 09/02/2018-E.8919), as well as from the Scientific Research and Ethical Committee of Artvin Çoruh University in Turkey (Issue date-no: 14/02/2018-E.2708). Ilia State University in Georgia was informed, and necessary official scientific research and travel permits were obtained from the Department of Land Border Protection of the Border Police of the Ministry of Internal Affairs of Georgia.

Initial investigation considered the flora in different vegetation zones (forest, meadow, wetlands, steppe, rocky areas) en route to and in the vicinity of each selected highland pasture. This process took around 2–3 h for each highland pasture. This reconnaissance involved the identification of wild plants, where possible to species level, in which photographs and a minimum of three samples were collected for each. This had a dual purpose, firstly to be able to show participants for them to identify plants they would talk about, and secondly to prepare herbarium voucher specimens for later detailed identification.

The research team was composed of three or four people. The first (female) and second (male) authors were always involved in the interviews, with a translator—either male or female. Throughout the study, the first author was always the principal interviewer. In Turkey, the interviews were conducted directly in the Turkish language, while in Georgia, interviews were in Georgian, Russian, or Turkish. The majority of the interviews in Georgia were performed with the help of translators who spoke Georgian, Russian, and English, either as a mother tongue or as a second language. Interviews were later translated into English. Two weeks before the fieldwork, the translators were provided with information and terminology relevant to the research. Information relating to the purpose of the study was given to all participants and their free, prior informed consent—for interviewing, recording, photographing, and/or publishing their knowledge—obtained orally from each at the beginning of their interviews. All interviews conformed to the International Society of Ethnobiology’s Code of Ethics [37]. The obligations of the Nagoya Protocol also being considered, it was approved that “the right of use and ownership of any traditional knowledge of all informants remains with them, and that any use of the information except for scientific publication, requires the additional consent of the traditional owners, and consensus on access to benefits derived possibly later use” [38].

A snowball technique was used to find participants who held significant traditional knowledge regarding wild plants and their usage. The majority of the participants were elderly transhumant people. Each was interviewed individually, for an average of 2 h, with semi-structured questionnaires. Usually, the person’s relatives and neighbors also contributed to the interview. The first author took notes directly in a notebook during all interviews. Depending on participants’ wishes, audio or video recordings were made of the interviews. Information about plants collected from the wild was documented, specifically with data regarding (1) their folk names in different languages and dialects, (2) collection time and place, (3) parts used for medicinal purposes, (4) processes of preparation and administration, and (5) source of the plant knowledge. In addition, observations were made and photos taken in byres, cellars, and other relevant places whenever possible so as to document unmentioned uses and also observe living ethnobotanical practices.

Initially, participants were asked to discuss points about wild plants that immediately came to mind (~ 15 min). Then they were shown fresh plants and asked to identify the vernacular names and usage of the plants (~ 45 min). Depending on the weather and participants’ willingness, a “walk around the house” was undertaken to observe wild plants in the proximity (~ 15 min). To confirm previous information and to gain further learning about various plants, participants were shown an illustrated plant catalog, including 400 plant species from the flora of the region (~ 45 min.). Certain participants were visited a second time to complete the first interview or to confirm information.

### Taxonomic identification of plants

Preliminary identification of plant species was carried out in the field by the authors. The plants were photographed with their coordinates and then voucher herbarium specimens were prepared by the first author for further identification. Relevant flora resources were used for identification [39–44]. Identified specimens from Georgia were checked by the third author, and stored in the National Herbarium of Georgia and in Ilia State University. Specimens identified in Turkey were checked by Prof. Özgür Eminağaoğlu and stored in the Herbarium of Artvin Çoruh University. Species were named based on current accepted names [45]. Furthermore, plant synonyms were given after consulting “Plant List of Turkey–Vascular Plants” [46] and “Vascular Plants of Georgia–A nomenclatural checklist” [47].

### Data analysis

Firstly, all reported plant species and their relevant ethnobotanical data were entered into a Microsoft® Excel spreadsheet in a use-report (UR) based order, following the categories in the Economic Botany Data Collection Standard [48]. Under each use-category, each different use was counted as one UR. For example, if a participant mentioned a species used for headache and coughing, it counted as 2 UR. Secondly, the data of wild plants and their related medicinal use reports were extracted. After a final classification of the categories, pivot tables were constructed for further analysis. The final classification of reported ailments and diseases considered The International Classification of Primary Care (ICPC-2-R) developed by World Organization of Family Doctors [49] and accepted by the World Health Organization (WHO), on suggestions by several authors of comparative ethnomedicinal studies [50, 51]. Thirteen medicinal use-categories for several emic subcategories were determined (Table 1).

**Table 1:** Assigned medicinal use categories for reported ailments and uses

Medicinal use categories	Ailments/Uses
Digestive	Constipation, diarrhea, dysentery, gastrointestinal infection, indigestion, liver disease, stomach ache, tooth bleaching, tooth inflammation, toothache, worm, clean intestine, gall disease, intestinal disease, jaundice, ulcer (mouth, stomach), induce vomiting, prevent vomiting
Respiratory	Asthma, bronchitis, cold, cough, influenza, lung disease, throat ache, throat inflammation, tonsil, shortness of breath, expectoration, nasal obstruction, mumps, quinsy
Cardiovascular	Vasodilator, hemorrhoids, heart disease, high blood pressure, varicose vein, blood circulation
Skin	Antiseptic, blister, boil, bruises, burn, eczema, irritation, wart, wound, antifungal, itching, belief (wart), psoriasis, rash, insect and snake bites, bleeding wound, skin care complaints, callus, chap, hair care complaints (dandruff, hair loss, wash and growth), herpes, inflammation
Endocrine	Diabetes, thyroid, increase milk supply, loss of appetite, sexual development (induce estrogen level)
General health and unspecified	Allergy, cleaning organs, fever, general disease, good for health, measles, tiredness and weakness, tuberculosis, vitamin deficiency, cancer, chickenpox, feeling ill, pain killer, sunstroke
Genitourinary	High menstrual bleeding, incontinence urine, kidney disease, kidney pain, kidney stone, prostate, urinary disease, vaginal discharge, women disease (infertility, inflammation), abortion, diuretic, bladder infection, man disease (infertility), menstruation problems, menopausal complaint
Muscle-skeletal	back pain, bone and joint pain, rheumatism, sprain, knee ache, fracture, cramp, footsoreness, induce synovia, numbness in arm, rachitic
Eye	Eye diseases, good for eyes, hypopyon
Ear	Earache
Blood	Anemia, cleansing blood, hematinic, iron deficiency
Neurological	Headache, dizziness, epilepsy, increase memory, nervous disease
Psychological	Relaxing, sedative, sleep disorders

To reduce error and better confirm the local perceptions of participants, minor modifications were made to the ICPC categories.

This paper considers ethnomedicinal data of wild plant species encountered during the 2017 and 2018 fieldwork for the current data analysis. Ethnobotanical interviews and data analysis focused on “wild” (non-cultivated) plants native to the study area. For instance, species such as *Malus domestica*, *Prunus x domestica*, *Morus alba*, *Robinia pseudoacacia*, etc. were excluded from the study since they are either widely cultured or exotic. Although they are also cultivated, *Corylus avellena*, *Juglans regia*, *Ribes rubrum*, etc. were included as they are native to the area or have run wild and become naturalized. There was no focus on or promotion of exotic plant species or unconventional (introduced) knowledge related to them. This choice was made not only for logistical reasons but also because even though exotic plants may somewhat enrich local ethnobotanical knowledge [52], there are also documented adverse effects on local biodiversity, ecosystem services and local community livelihoods as well as cultural diversity [52–54]. Furthermore, participant knowledge and perceptions of plant collecting places were used to decide which species qualified as “wild.” Based upon these criteria, a total of 152 wild plant species were included in the analysis.

Firstly, the number of species mentioned on both sides of the Georgia-Turkey border were compared using Venn diagrams. This illustrates the level of species richness and shared species for the evaluation of regional ethnomedicinal knowledge.

Secondly, common and distinct ethnomedicinal knowledge and use were compared among communities researched on both sites of the border as well as with relevant ethnobotanical literature in the Caucasus Ecoregion. A species-use combinations approach involved both medicinal sub-categories (reported ailments) and medicinal categories (e.g., digestive, respiratory, etc.). This presents the level of shared use/knowledge incidence and its variations among communities studied. The shared knowledge is given in bold, with knowledge unique to Turkey italicized and that unique to Georgia not italicized (Table S1).

Thirdly, the medicinally valuable plants were quantified and compared in function of most salient plant families, genera, species, as well as their plant parts, medicinal use, preparation, and application methods across the border. The following indices were used to evaluate the relative cultural significance, versatility of species and consensus on medicinal use and knowledge on the both sides of the border.

- The Cultural Importance index (CI): A widely used index in ethnobotanical studies (e.g., [55, 56]), it is known to produce reliable results in assessing the relative cultural significance of each plant species while comparing different regions with different participant numbers [57]. This is known to be effective not only in presenting the spread of use (number of participants) but also to highlight the diversity of uses (versatility) for certain plant species in cross-cultural ethnobotanical studies [57].

CI values are calculated by adding the number of use reports (UR) of all the participants in every use-category mentioned for a species, divided by the number of participants in the survey [57].

$$ \mathrm{CI}=\sum \limits_{i=1}^{i= NU}\frac{URI}{N} $$

NU: Total number of uses; *i*: varies from one use to NU; *N*: number of participants in the survey.UR: Use report.

In the case of this study, although the CI values of each species (CI_s_) were calculated separately (see in Table S1) instead of comparing only CI_s_, attention was paid to the Cultural Importance of each plant genus (CI_g_: total value of CI_s_ in the same genus) to reveal the more versatile genera. Besides, the cultural importance of each family (CI_f_) was computed by summing CI_g_ values for all genera of the same family [56]. These approaches will reduce the risks of under or over estimation of the CI for certain plant species due to misidentification of very similar species and subspecies; this would also compensate for possible effects of different folk taxonomic classifications among participants. It is known that closely related plant species share common natural products [58], whose type of usage may also clump phylogenetically [59].

Therefore, we believed that a CI_g_-based comparison would also serve as a sufficient indicator to illustrate key points of the study. The CI Index was also applied to medicinal use categories to estimate the contribution of each use category to CI_g_ [60, 61]. This time, the number of URs for each use-categories was divided by the number of participants in the survey.

b) Informant consensus factor (FIC): Another commonly used index for exploring potentially active medicinal plants for certain ailments, FIC was first proposed and used in medicinal ethnobotany studies to estimate the agreement of participants on a number of plant species according to specific use-categories (illnesses or ailments) [62].

FIC values are obtained as follows: number of use-reports in each use category (*n*_ur_) minus the number of taxa used (*n*_t_), divided by the number of use-reports in each category minus one [62, 63].

$$ \mathrm{Fic}=\left(\mathrm{nur}- nt\right)/\left( nur-1\right) $$

*n*_ur_: number of use-reports in each use category; *n*_t_: number of taxa used for that use category.Where FIC ranges from 0 to 1.

In this study, by using FIC index, the level of agreement of participants on the ethnomedicinal knowledge of wild plants species were evaluated.

FIC values close to 1 reflect a high consensus on a certain plant species for a given ailment (use-categories). On the other hand, FIC closer to 0 (zero) would indicate either a high degree of intracultural variation or a significant lack of documentation of the participants’ knowledge [64].

## Results and discussion

### Overall results, comparisons, and extrapolations

**Fig. 2:**
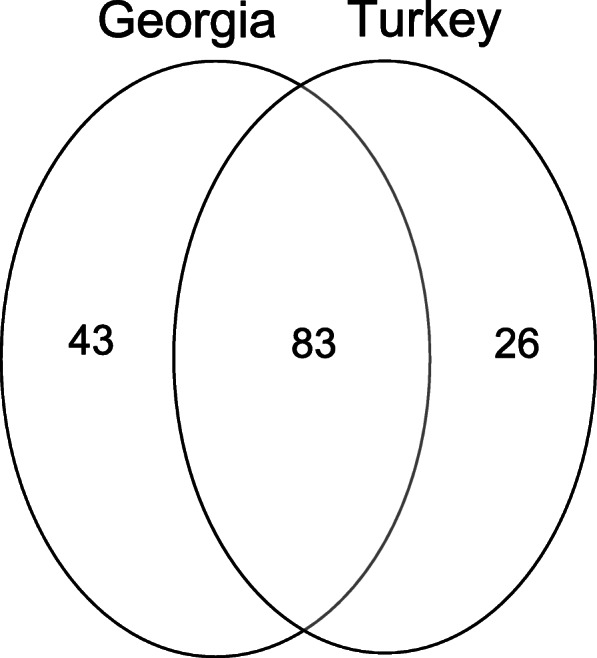
Overlaps between the recorded numbers of wild plant species among studied communities. *Note that the studied sites in Turkey include highland settlements of Artvin and Ardahan; the studied sites in Georgia include highland settlements of Adjara and Samtshke-Javakheti around the border

One thousand five hundred six use-reports for 152 native wild plant species are documented for medical purposes in the study area. Figure 2 shows the overlaps between the recorded numbers of wild plant species among studied communities. The regions on either side of the border share 83 of the reported plant species. Considering the fact that almost the same flora is found in the two regions, the level of similarity is low. In addition, the number of unique species (43 species) mentioned only by participants in Georgia is significantly higher than the number of unique species (26 species) mentioned only in Turkey (Fig. 2).

**Fig. 3:**
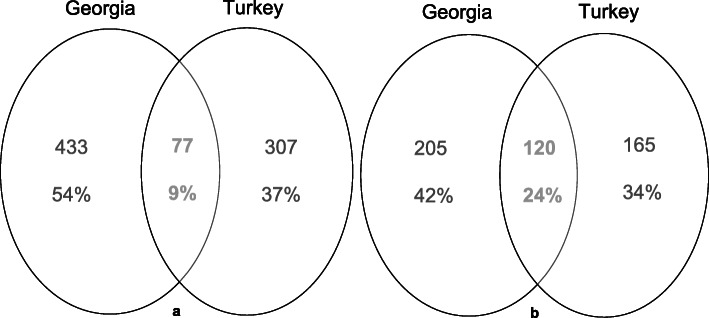
Overlap of species-use combinations. **a** comparing the emic/folk use category and **b** comparing medicinal use category

Table S1 summarizes the information about use reports of the 152 wild plant species and comparison with the literature sources. In the comparison of the 817 distinct species-use combinations based on medicinal emic categories (e.g., asthma, ulcer, wound healing, etc.), participants in both countries share similar medicinal knowledge of only 77 use incidences (9% of total species-use combinations) for 33 wild plant species (22% of reported species) in common (Fig. 3a). Most important of these species based on CI index are *Plantago major*, *Urtica dioica*, *Picea orientalis*, *Anthemis* spp., *Sambucus ebulus*, *Achillea millefolium*, *Helichrysum rubicundum*, *Mentha longifolia*, *Pinus sylvestris* var. *hamata*, *Hypericum perforatum*, *Tussilago farfara*, *Helichrysum plicatum*, *Rumex crispus*, *Berberis vulgaris*, and *Origanum vulgare*. In contrast, 433 distinct uses were reported only from Georgia, whereas only 307 unique uses were reported from Turkey.

When comparisons were based on medicinal use categories (e.g., digestive, respiratory, cardiovascular system etc.), out of 490 species-use combinations, only 120 (24% of the reports) of these were found to be in common in both countries (Fig. 3b). The results in Fig. 3 a and b indicate significantly low consensus of medicinal knowledge about shared native wild flora across the border. Participants in both countries use a significant number of shared species for different purposes. This lack of shared ethnomedicinal knowledge might be a sign of different epidemiology of certain ailments in communities studied as well as various medicinal knowledge systems in ethnolinguistically diverse communities on both sites of the border.

Table S1 reflects similarities and differences seen between information reported in this study and reports from 26 main literature sources related to folk knowledge of medicinal plants from the Caucasus Ecoregion, mainly North-Eastern and Eastern Black Sea Region of Turkey and Georgia, as well as Armenia and Azerbaijan, including literature from Soviet period. Of this study’s 817 distinct species-use combinations based on medicinal sub-categories (asthma, ulcer, wound healing, etc.), only 275 of them are similar with those in the above mentioned literature reports. This means that around 66% of this study’s use reports (as a species-medicinal subcategory combination) have not been mentioned specifically in these items of literature. Even, when comparisons are based on medicinal use categories, out of 490 species use combinations, only 276 of them are similar and around 44% of these use reports also have not been mentioned in these literature sources.

Nevertheless, although very limited, the fact that a number of plant species and similar medicinal reports are in common with various areas of the Caucasus Ecoregion would be indicative of consensus and high cultural value for the medicinal knowledge of certain wild plant species in the Ecoregion. The most important fifteen wild plant species consistent with both this study’s reports and those in literature sources are *Urtica dioica*, *Plantago major*, *Mentha longifolia*, *Hypericum perforatum*, *Pinus sylvestris* var. *hamata*, *Rosa canina*, *Achillea millefolium*, *Berberis vulgaris*, *Chelidonium majus*, *Sambucus ebulus*, *Vaccinium myrtillus*, *Picea orientalis*, *Helichrysum plicatum*, *Tussilago farfara*, and *Hyoscyamus niger*.

### Cultural importance index of families, genera and species

**Fig. 4:**
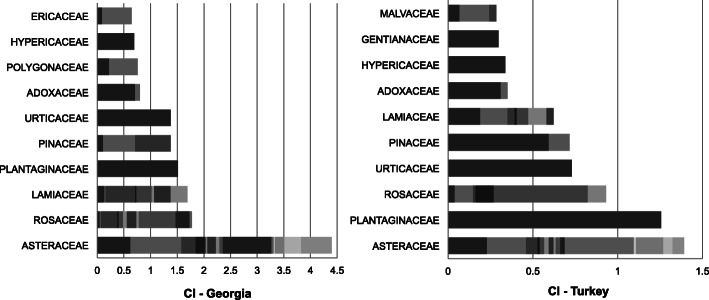
Cultural importance (CI) index of the 10 most important families in Georgia (left) and in Turkey (right). Each gray tones reflects the contribution of different genus to the total CI of each family

**Fig. 5:**
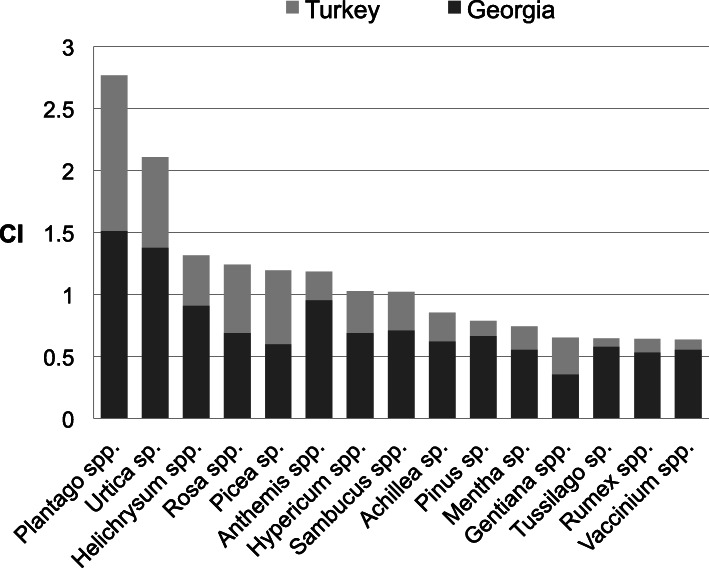
Cultural importance (CI) index of the 15 most important genera in the study area. (spp. indicates the contribution of more than one species, while sp. indicates the contribution of only one species)

Figure 4 shows the cultural importance (CI) index of most important families reported in Georgia and in Turkey. Out of 36 plant families recorded on the whole study area, 25 of them were recorded in both countries, whereas 8 of them were only from Georgia and 3 of them were only from Turkey. The three most important families according to CI index in order of importance in Georgia are Asteraceae, Rosaceae, and Lamiaceae while in Turkey they are Asteraceae, Plantaginaceae, and Rosaceae. Most of the important families are represented by several genus in Georgia, while most of them are represented by one or two genus in Turkey. The most important genera in order of importance are *Plantago*, *Urtica*, *Anthemis*, *Helichrysum*, *Sambucus* in Georgia and *Plantago*, *Urtica*, *Picea*, *Rosa*, *Helichrysum* in Turkey (Fig. 5).

Asteraceae is seen to have the highest number of genus (16 in Georgia; 15 in Turkey) in both countries. The CI index of this family in Georgia is more than threefold that of Turkey. Significant differences in CI are also noted at the species level. While *Anthemis* spp. (CI 0.96) are the most important species following *Achillea millefolium* (CI 0.62) on the Georgian side, in Turkey *Helichrysum rubicundum* (CI 0.28) is the most important, following *Anthemis* spp. and *Achillea millefolium* with same CI value of 0.23. These results might be a sign of differences in the level of knowledge regarding medicinal use of this plants among studied communities. The Rosaceae is the second culturally most important family in Georgia and third in Turkey, mostly respresented by *Rosa* genus comprising approximately half of the CI value of Rosaceae in both countries. Although several *Rosa* species have been recorded for medicinal use, a particular selection between two different species between two countries is noted. While *Rosa canina* with a CI of 0.31 is the most important *Rosa* species in Georgia; *Rosa spinosissima* (syn. *R. pimpinellifolia*) with a CI of 0.32 is the most important *Rosa* species in Turkey, being more than threefold that the CI index of *R. spinosissima* in Georgia (0.09). The small CI index value of *Rosa spinosissima* in Georgia may be due to the fact that it has a narrow distribution and low abundance in Georgia. However, as far as observed, this species has a relatively wider distribution and high abundance in Turkey. Another possible and more plausible explanation is that the particular medicinal use of this species in Turkey, especially in Ardahan, could be related to similar cultural background and unique ethnomedicinal knowledge of the people living there. The frequent use of *Rosa spinosissima* roots as medicinal tea in this province is an uncommon medicinal use, which has so far rarely been cited in the literature in Turkey. One previous report was from Çıldır, Ardahan [23] within the area of this study, while a further two more reports were from the nearby region, in Erzurum [65, 66]. Consensus in this traditional knowledge in Ardahan is likely to reflect the therapeutic efficacy of this plant, a point of interest to be noted for further ethnobotanical and ethnopharmacological studies.

Plantaginaceae is the second most important family in Turkey and fourth in Georgia. Their CI vlaues are close to each other (1.51 in Georgia; 1.26 in Turkey). *Plantago* spp. (*P. major and P*. *lancelolata*) are in the first place within the top 15 culturally salient genera in both countries. Indeed, *Plantago major* is one of the most frequently reported medicinal species in cited literature (Table S1). In addition, the third family in Georgia, Lamiaceae CI, is significantly greater than that in Turkey, being 1.69 and 0.62 respectively. Even the most reported genera in both countries are the same (*Mentha* and *Origanum*); *Mentha* with CI of 0.56 in Georgia and 0.19 in Turkey is a sign of significant differences between the two countries. Actually, *Mentha* is a widely known, reported, and used plant species as a food additive in the study area, in Turkey. Therefore, its low medicinal CI value could be related to its less known specific medicinal properties. Same reason might be relevant for Polygonaceae and Ericaceae families which are not represented in CI index of top ten families in Turkey. *Polygonum* spp., *Rumex* spp., and *Vaccinium* spp. are not consumed specifically as medicine but mainly known as food in Turkey.

### Plant parts used and preparation and administration methods

More than one plant part is known/used medicinally for 54% of the wild plants mentioned in Georgia and 57% of them in Turkey. Most commonly, the leaves is given for 27% of the URs in Georgia and 26% of URs in Turkey (Table 2). *Plantago* spp. constitute majority of the leaf use reports in both countries. The use of aerial parts with flower comes after leaf use in both countries. Most of the contribution to this use originates from *Helichrysum* spp., Achillea millefolium, Mentha longifolia and Origanum vulgare. Fruits have also similar use percentage in each country. *Rosa* spp., *Sambucus* spp., and *Crateagus* spp. are frequently used species for their fruits in the study area.

**Table 2:** Comparison of URs (%) for plant parts used, preparations, and applications in Georgia and in Turkey^a^

Plant parts used	UR (%)	UR (%)	Preparations	UR (%)	UR (%)	Applications	UR (%)	UR (%)
	Geo	Tur		Geo	Tur		Geo	Tur
Leaves	26.66%	25.83%	Infusion in water/milk/votka/oil/whey/sugar	47.99%	32.33%	Drink	49.41%	37.01%
Aerial parts with flowers	17.77%	19.49%	Decoction in water/milk/oil	22.63%	29.91%	Plaster	15.76%	22.51%
Fruits	13.98%	10.27%	Fresh or mixed with honey/olive/salt/another plant/beewax/spores/votka/yoghurt/resin	18.25%	23.72%	Bath	15.05%	20.54%
Aerial parts	11.26%	11.78%	Macerated	4.50%	7.70%	Eat	11.02%	11.78%
Flowers	9.36%	8.46%	Sweet dishes	2.37%	1.21%	Gargle	4.38%	1.06%
Roots/bulbs	5.33%	5.89%	Juice (fresh, vinegar)	1.54%	0.60%	Chew	1.90%	3.02%
Resin	3.08%	7.10%	Warmed on stove	1.18%	1.06%	Inhalation	1.07%	2.42%
Seeds	1.54%	4.38%	Burned	0.24%	1.96%	Embrocation	0.83%	0.60%
Entire plant	3.44%	1.36%	Ritual	0.71%	0.45%	Headbands	0.24%	0.45%
Branches	1.42%	2.57%	Dried and powdered	0.12%	0.76%	Ritual	0.12%	0.45%
Young seed cones	1.42%	0.76%	As vegetable (pickle, soup)	0.47%	0.30%	Fumigate	–	0.15%
Pollen cones	1.66%	0.15%				Tamp	0.12%	–
Outer barks	1.54%	–				Beating of skin	0.12%	–
Sap	0.47%	0.76%						
Young seed cones and pollen cones	0.71%	–						
Inner barks	0.24%	0.30%						
Pericarp/husk	–	0.45%						
Aerial roots	–	0.30%						
Heartwood	0.12%	–						
Receptacles	–	0.15%						

^a^Note that the information (words) in each row do not match with each other (see in Table 2 for the preparation and application of each plant part). Information was ranked in accordance of importance based on mean UR (%) values of the countries

Among the preparation methods, the most common one in both countries is infusion in water/milk/votka/oil/whey/sugar (48% of URs in Georgia, 32% of URs in Turkey). They are followed by decoction in water/milk/oil and fresh use.

A major application method in both countries is internally, specifically, drinking or eating. In Georgia, drinking and eating constitute 49% and 11% of the URs respectively; in Turkey, the corresponding UR data are 37% and 12% respectively. Plastering and bathing are major external application in both countries.

To summarize, communities in both countries utilize many parts of wild medicinal plants, with a variety of modes of preparation and application methods. However, the most common way of using wild plants medicinally in both countries is drinking the water infusion of aerial parts with flowers.

### Medicinal use-categories and salient species

In the study area on either side of the Georgian-Turkish border, the cultural importance of medicinal use categories as well as salient species used varies significantly among communities.

Table 3 shows the number of use-reports, number of species, cultural importance, and informant consensus for each medicinal use-categories. Comparison of the results in Table 3 reveals that ailments related to digestive and skin problems have the highest number of use-reports in both countries (in total, 43% of URs in Georgia, 49% of URs in Turkey). In order of importance, these categories are followed respectively by respiratory, cardiovascular, and muscle-skeletal disorders in both countries. Conversely, complaints related to ear, eye, blood, psychological, and neurological conditions have the least (or not any) use-reports in both countries. On the other hand, the informant consensus factor (FIC) in medicinal use categories are, in order of importance, digestive, skin, muscle-skeletal in Georgia and skin, digestive, muscle-skeletal in Turkey. There is no statistical consensus in the ear category in both countries; neither is there any consensus in the psychological and blood category in Turkey. However, it is noted that low (e.g., eye and neuro) or exceptionally high (e.g., in psych) FIC values in this table can be misleading since they were represented by very low use reports; it should not be interpreted as a high degree of intracultural variation but it might be a sign of insufficient documentation of participants’ knowledge [64].

**Table 3:** Number of use reports (URs), number of species, CI, and FIC values for each medicinal use category^a^

Medicinal use categories	Georgia				Turkey			
	♯ UR (%)	♯ of spp.	CI	FIC	♯ UR (%)	♯ of spp.	CI	FIC
Skin	163 (19.31%)	45	3.62	0.73	193 (29.15%)	47	2.61	0.76
Digestive	203 (24.05%)	51	4.51	0.75	128 (19.34%)	44	1.73	0.66
Respiratory	105 (12.44%)	37	2.33	0.65	75 (11.33%)	34	1.01	0.55
Cardiovascular	100 (11.85%)	50	2.22	0.51	69 (10.42%)	37	0.93	0.47
Muscle-skeletal	73 (8.65%)	25	1.62	0.67	58 (8.76%)	22	0.78	0.63
Genitourinary	69 (8.18%)	35	1.53	0.50	55 (8.31%)	34	0.74	0.39
Ghus	56 (6.64%)	37	1.24	0.35	23 (3.47%)	20	0.31	0.14
Endocrine	39 (4.62%)	24	0.87	0.39	35 (5.29%)	22	0.47	0.38
Neurological	11 (1.30%)	10	0.24	0.10	11 (1.66%)	10	0.15	0.10
Psychological	15 (1.78%)	5	0.33	0.71	2 (0.30%)	2	0.03	–
Blood	6 (0.71%)	4	0.13	0.40	7 (1.06%)	7	0.09	–
Eye	4 (0.47%)	3	0.09	0.33	4 (0.60%)	3	0.05	0.33
Ear	–	4	–	–	2 (0.30%)	2	0.03	–
Total	844 (100%)	126^b^	18.8		662 (100%)	109^b^	8.95	0.76

^a^Use categories were ranked in accordance of importance based on mean CI values

^b^As some species are cited in more than one use categories, these numbers (126 and 109) indicating the number of species recorded in Turkey and in Georgia are lower than the total of number of species recorded for each medicinal use category

To summarize, the most commonly cited and valued medicinal knowledge/use with high agreement among participants dealt with the treatment of digestive disorders in Georgia, whereas it is related with skin complains in Turkey, a result possibly pointing to the fact that communities living in the study area tend to suffer from ailments of these two medicinal categories. These use categories also have the highest wild plant richness in both countries.

### Skin

As stated previously, skin ailments are the most important category in Turkey (CI 2.61, FIC 0.76) comprising 29% of URs, while being the second category in Georgia (CI 3.62, FIC 0.73), with 19% of the URs (Table 3). Fifty-six percent of the URs mentioned by participants in Turkey are related to wound and boil. While Plantago major is the most important plant for boil treatment, *Picea orientalis and* Plantago major are the most important plants for wound healing in both countries. Plantago major leaves specifically is used for boils either macerated or plastered on skin after warming on stove in the study area. The use of Plantago major for boils and wounds was also frequently cited in the literature [12, 16, 17, 19–21, 23, 25, 65–72]. On the other hand, macerated resin of *Picea orientalis*, locally called “*pisi*,” is applied as a plaster for any kind of wound, a widely known application in Turkey, and is also mentioned for wounds and boils in Georgia. One unique report describes using it in a homemade wound healing salve, a mixture of *Picea* resin, beeswax, butter and olive oil. Such healing properties of *Picea orientalis* are also consistent with applications mentioned in several sources from Turkey [71, 73–76] and one source from Georgia [14]. In addition, *Urtica dioica and Anthemis* spp. are commonly reported species to treat hair care complaints by participants from both countries. A bath with water decoction of aerial parts of *Urtica dioica* or with the infusion of *Anthemis* spp. flowers are stated to treat hair care problems.

Sap of *Chelidonum majus* is the only shared species commonly used externally to cure warts in both countries. Magic rituals are also reported for this skin disorder. *Alnus glutinosa* and *Salix* spp. are the ones to cure warts in Turkey. With the former, as many *Alnus glutinosa* leaves as the number of warts are hung on the wall. It is thus believed that the warts will disappear when the leaves dry and fall to the ground. In the latter practice, people carve as many notches as the number of warts in a branch of *Salix* spp. and keep it over the roof of the house. The wart is predicted to disappear when the branch falls from the roof.

### Digestive system

The most widely used traditional cures for digestive complaints differ in both countries. In Georgia, the species with highest cultural importance in this category is Plantago major (CI_s_ 0.42), whereas in Turkey, *Picea orientalis* has the highest CI_s_ (0.28) for digestive problems. URs for Plantago major in Georgia and *Picea orientalis* in Turkey mainly stem from stomachache. Among the applications, drinking a water infusion of the leaves of Plantago major is commonly known for treating stomachache in Georgia. The use of Plantago major for stomach-related disorders was also reported in both countries [12, 16, 19, 66, 67, 75, 77]. Moreover, fresh resin of *Picea orientalis* is found to be commonly used as chewing gum or swallowed for stomach ache in Turkey. The use of *Picea* resin for stomach-related disorders was also reported in both countries [12, 71, 75, 76].

*Berberis vulgaris* is another important species for digestive problems with similar cultural importance in each country. Jaundice is the only illness mentioned for its use in Turkey. Participants frequently called it “*sarılık ağacı*” which literally means “the tree of yellowness” in Turkish. Similarly, it is frequently said to cure jaundice and liver-associated diseases in Georgia. Drinking of or/and bathing in a water decoction of *Berberis vulgaris* branches are the common mode of applications for jaundice in both countries. Using roots, bark, and also fruits had been previously noted for treatment of jaundice in Georgia [78]. An infusion of the fruit in salty water had been reported to cure jaundice in Azerbaijan [78]. Similar medicinal knowledge was reported close to the current study area only in the Turkish province of Erzurum. Bathing and drinking a water decoction of *Berberis vulgaris* root, and licking the root of *Berberis vulgaris x crateagina* for curing jaundice in this province were mentioned in several studies [20, 65, 66, 79].

The other commonly mentioned digestive-related problems and plants are Sambucus ebulus used for constipation and, *Anthemis* spp. and *Quercus petrea* species used to cure tooth ailments. Interestingly, only *Quercus petrea* has no mentions in Turkey. Among the applications, gargling a water decoction of the outer bark of Quercus petraea is commonly known for treating toothache in Georgia. There is not any reports of medicinal usage of *Quercus* species in previous studies conducted in the Turkish part of the Caucasus or even in the ethnomedicinal review for East Anatolia [80]. However, a similar use was reported in Georgian sources [81]. Thus, its unique medicinal use knowledge recorded in Georgia might be attributed to cultural background as well as to the remedies written in the literature, especially in ancient traditional medical books. Indeed, when participants were asked about the origin of their ethnomedicinal knowledge, although most in Georgia referred to their elders’ knowledge as a primary source, some also acknowledged their primary school education and others recalled Russian botanists who had conducted research in the region. Furthermore, several referred to the fifteenth century Georgian book “Karabadini,” which had formerly been known as “Ustsoro Karabadini,” the first almanac of medicinal remedies and medicinal knowledge, written by Kananeli Karaba in the tenth century. A further source of knowledge mentioned was the “Turmanidze family,” an acknowledged traditional Georgian medical family. Some also made a reference to Russian medicinal plant guidebooks and web pages. In contrast, no similar examples was mentioned by participants in Turkey, whose sole source of plant knowledge was their elders and people around. They also complained about lack of scientifically sound medicinal plant sources in Turkish. Georgian participants’ multilingualism enabled them to access a diverse range of literature related to medicinal plants that could account to some degree for the resulting variation in people’s ethnomedicinal knowledge across this border.

### Respiratory system

In accordance with measures of cultural importance (CI), diseases related to the respiratory system are the third most important medicinal category in both countries. The species with the highest cultural importance for respiratory diseases are *Pinus sylvestris* var. *hamata*. (CI_s_ 0.40) and *Tussilago farfara* (CI_s_ 0.38) in Georgia. In contrast in Turkey, *Helichrysum rubicundum* (CI_s_ 0.14) and *Rosa spinosissima* (CI_s_ 0.09) are mostly used for respiratory diseases.

The most common respiratory ailments in Georgia are cough (30% of respiratory URs), lung diseases (20%), and bronchitis (15%). Water infusions of young seed cones and pollen cones, or a mixture of both plant parts of *Pinus slyvestris* var. *hamata* are widely known tea remedies for treating common respiratory diseases. Mainly pollen, as well as cone, have been reported for these disorders and for asthma, from different parts of Georgia [12, 13, 15]. On the other hand, a decoction of the needles for cough was reported in Azerbaijan [82]. However, in Turkey, *Pinus sylvestris* has a very low value (CI_s_ 0.03). Only the infusion of young seed cones are reported for cough and asthma with a UR value of 2.

Another treatment reported only in Georgia, to be good for asthma, bronchitis and lung diseases is a jam made of young seed cones and pollen cones and also pollen cones mixed with honey. Indeed, during fieldwork in Georgia, the sale in local bazaars of fresh *Pinus sylvestris* cones and pollens as a medicine was frequently observed, traders inviting shoppers to taste their pollen. One participant offered jam made of young seed cones of *Pinus sylvestris*. This events revealed the continuing existence of a living traditional phytomedicine heritage in Georgia. In contrast, no participant in Turkey gave any clue of similar traditions related to *Pinus sylvestris*. In fact, most were astonished to hear of the usage of *Pinus* cones as a jam. Although the species is homogeneously distributed across the study area, and used across the border in various ways (material, construction, fuel), the greatly contrasting high medicinal CI values in Georgia and very low CI values in Turkey might be associated with cultural background of the communities as well as participants’ in Georgia accessing medicinal knowledge in multilingual literature sources. The high regard of local people for the Russian name, “sosna,” for *Pinus sylvestris* might be a reflection of multilingual literature on medicinal knowledge in Georgia. Similary, the leaves of *Tussilago farfara* has a wide range of uses in respiratory diseases (cough, bronchitis, asthma, lung diseases) in Georgia but in Turkey it is not reported in this research, even though it was widely reported in the literature [12, 14, 16, 17, 72, 83, 84].

Cough (14% of the respiratory URs), throatache (5%), bronchitis (5%), and asthma (4%) are the most commonly mentioned diseases in Turkey. A water infusion or decoction of *Helichrysum rubicundum* aerial parts with flowers, for cough and throat complains, is one of the most mentioned remedy (10 UR) only in Turkey. Its use in respiratory disease was scarcely mentioned in the cited literature in Turkey [65]. A water decoction of *Rosa spinosissima* root, mainly for cough and bronchitis, is another common remedy in Turkey. Furthermore, a water decoction of its fruits was given for cough which had also been reported from Turkey before [77]. Water decoction of roots is also mentioned to cure influenza, which has also been reported in this and other studies [23, 67]. Interestingly, for Georgia and other neighboring countries, no use-reports were found to be specified for *Rosa spinosissima* (syn. *R. pimpinellifolia*) or *Helichrysum rubicundum* for these respiratory disorders.

### Cardiovascular system

It is the fourth most important medicinal use category in Georgia (CI 2.22) and in Turkey (CI 0.93). Hemorrhoid (including its itching symptoms) is the most widely mentioned complaint among the participants in Turkey (66 UR, 39% of the cardiovascular URs). Similarly, in Georgia, it is comprising 31% of cardiovascular URs.

In Turkey, *Rosa* spp., in particular, *R. canina* (4 UR) and *R. spinosissima* (7 UR), are the most important species used against hemorrhoids. Fruits of *R. canina* and roots of *R. spinosissima* (occasionally their fruits) are widely known/used for hemorrhoids by participants in Turkey. A review related to plants used to treat hemorrhoids identified *Rosa canina* as most frequently used species in Turkey, while *Rosa spinosissima* (syn. *R. pimpinellifolia*) was mentioned in only one study conducted in Erzurum in 1999–2000 [79] in which only fruits, not roots, were reported to be used [85]. Later research did report the use of roots [66] and several other studies mentioned the use of fruits [70, 77]. The use of fruits and/or roots together to treat hemorrhoids was also reported as well [65]. However, in this particular fieldwork in Georgia, among *Rosa* spp., only *Rosa canina* (2 UR) was mentioned for hemorrhoids. Furthermore, neither *R. spinosissima* nor *R. canina* was mentioned for hemorrhoids in the sources from Georgia, Azerbaijan, or Armenia [86]. *Gentiana* spp. (9 UR) are widely recorded second species in Turkey for hemorrhoids.

On the other hand in Georgia, *Gentiana* spp. are the most important ones, mainly G*.* septemfida (8 UR) is used for hemorrhoids. A bathing water decoction or drinking water infusion of aerial parts with flowers is applied for treatment. This species has not been reported for hemorrhoids in the cited literature. Here, high blood pressure and hearth disease are the other major reports (65% of cardiovascular URs). The tea of *Helichrysum plicatum*, *Mentha longifolia*, and *Thymus* spp. are widely known to decrease high blood pressure. *Crataegus* spp. have notable importance (11 UR) for heart health in Georgia, where the fruits of these species are eaten fresh, consumed as compote or drunk as tea. Several studies from Georgia [12, 16, 87] and relevant sources from Azerbaijan and Armenia [88] also mention similar use-reports of fruits, flowers, or leaves of *Crataegus* spp. (mainly *C. pentagyna* and *C. monogyna*).

### Muscle-skeletal system

Muscle-skeletal complaints are among the top five medicinal use categories in both Georgia (CI 1.62) and Turkey (CI 0.78) with relatively high FIC values (0.67 in Georgia; 0.63 in Turkey) in both countries. Bone and joint pain, and rheumatism problems are widely reported by the participants in both countries (34% of the muscle-skeletal URs in Tukey; 74% of them in Georgia). A bathing in water decoction of Urtica dioica aerial parts or Equisetum arvense aerial parts is the most commonly used remedy in both countries. Plastering of body with fresh leaves of U*.* dioica is also applied for rheumatism in the study area. Among the rheumatism reports, local names given for the species indicate a very diverse ethnolinguistic background of the communities. The use of U*.* dioica for rheumatism is also frequently cited in the literature [18, 20, 21, 23, 65, 66, 69–71, 75, 76, 83]. These consensus might show the potential activity of the Urtica dioica for rheumatism. However, Equisetum arvense for rheumatism was only reported in one literature [83]. Moreover, a serious muscle-skeletal disease, rachitism, is mentioned only from Georgia. Bathing in water decoction or plastering of Tussilago farfara leaves, and bathing in water decoction of Equisetum arvense aerial parts are suggested as its remedies in Adjara.

### Genitourinary system

In this study, genitourinary disorders are more commonly mentioned in Georgia (69 UR, CI 1.53) than in Turkey (55 UR, CI 0.74). The majority of the use reports (64%) in Georgia concern the kidneys (kidney stones, pains, and other kidney diseases), while female (vaginal discharge, abortion, infertility) and male (prostate) genital conditions are proportionally more reported (60% of URs) in Turkey than in Georgia. *Helichrysum* spp. (*H. plicatum*, *H*. *rubicundum*) and *Rosa* spp. (mostly *R. canina*) are most frequently mentioned species for kidney related problems in Georgia. *Helichrysum* spp. were also widely reported species to cure kidney problems in the cited literature [18, 66, 69, 70, 73, 76, 77], but Rosa canina has few reports [18, 77]. In addition, *Plantago major* is used for various genitourinary diseases in Turkey. Drinking water infusion of *Helichrysum* spp. aerial parts with flowers, and water infusions or decoctions of *Rosa* fruits are the most common remedy for kidney-related problems in Georgia, whereas in Turkey drinking water decoction of *Rosa* spp. are mentioned to be good for the prostate. On the other hand, bathing with *Helichrysum* spp. is reported for women diseases in Turkey. Interestingly, there are several plants mentioned for abortion only in Turkey namely *Malva neglecta*, *Cephalaria gigantea*, *Achillea millefolium*, *Sambucus ebulus*, and *Viscum album*.

### General health and unspecified illnesses

The use of wild plants as preventive medicine for general health has 6.6% of the total medicinal use reports in Georgia, while in Turkey this constitutes 3.5% of them. A cure-all (panacea) and using plants against fever and pain are the most common emic categories in both countries. *Origanum vulgare*, *Ribes* spp., and *Satureja spicigera* species are mentioned for them in Georgia, while *Rosa*, *Heracleum*, and *Thymus* species are mentioned in Turkey. *Ribes* spp. are frequently mentioned against fever in Georgia, a use not previously specified in the literature. Moreover, *Picea orientalis*, *Pinus sylvestris* var. *hamata*, *Abies nordmanniana*, and *Tussilago farfara* are mentioned by participants for use against tuberculosis in Georgia. Others have previously reported these species except *Tussilago farfara* for the treatment of tuberculosis in Georgia [12, 16], while a similar record was made for *Picea orientalis* in Turkey [71].

### Endocrine system

Diabetes is the most commonly mentioned disease in both countries. It constitutes 69% of the endocrine reports in Georgia and 77% of them in Turkey. *Vaccinium* spp. (*V*. *mrytillus*, *V*. *arctostaphylos*, and *V. uliginosum*) and *Satureja spicigera* are the most important species used for the treatment of diabetes in Georgia, while in Turkey, *Crataegus* spp. (*C. monogyna*, *C. azarolus*, *C. pentagyna*) and *Sorbus aucaparia* are the most important species. Aerial parts of *Satureja spicigera* mixed with yoghurt is eaten or water infusion of the aerial part is drunk in Georgia. On the other hand, water infusion of young branches of both *Vaccinium* spp. with leaves is a widespread application employed for the treatment of diabetes by participants in Georgia, with one report of the eating of their fresh fruits. The same details, especially for *V*. *arctostaphylos*, were also mentioned in several studies from Georgia only [12, 16, 17, 25]. Similarly, although less commonly reported, *V*. *mrytillus* was mentioned in the same ways in Turkey, with fruits or/and leaves of *V*. *mrytillus* having been reported for diabetes around the study area, and in general in the Eastern Black Sea Region of Turkey [21, 68, 73, 74]. Moreover, the eating of fresh ripe fruits of *Crataegus* spp. and *Sorbus aucuparia* are recorded as the common treatment for diabetes in Turkey and, although uncommon, the same use is also reported in Georgia. The utilization of *Sorbus aucuparia* against diabetes has not been previously reported in/around this particular study region either in Turkey or in Georgia although its antidiabetic uses were reported in several sources [89]. Similarly, *Crataegus* spp. (C*.* azarolus var. *pontica*, C*.* monogyna, C*.* pentagyna) have not been mentioned as antidiabetic in the cited references (Table S1).

In summary, the six medicinal use categories mentioned above are represented by 17 plant species having at least seven UR in one of these categories in either country. They are ranked in accordance with the number of mean UR for each use category in Table 4.

**Table 4:** The most salient species with more than 6 UR for a medicinal category in either country

Latin name	UR Georgia	UR Turkey
Skin		
*Plantago major* L.	38	65
*Urtica dioica* L.	27	19
*Picea orientalis* (L.) Peterm.	8	18
*Anthemis* sp.	14	7
*Achillea millefolium* L.	10	5
Digestive		
*Picea orientalis* (L.) Peterm.	8	21
*Plantago major* L.	19	7
*Berberis vulgaris* L.	8	14
*Anthemis* sp.	18	2
*Sambucus ebulus* L.	16	4
*Achillea millefolium* L.	12	6
*Hyoscyamus niger* L.	6	12
*Hypericum perforatum* L.	9	3
*Quercus petraea subsp. iberica* (Steven ex M.Bieb.) Krassiln	11	–
Respiratory		
*Pinus sylvestris* var. *hamata* Steven	18	2
*Tussilago farfara* L.	17	–
*Picea orientalis* (L.) Peterm.	9	1
*Helichrysum rubicundum* (K.Koch) Bornm.	–	10
*Rosa spinosissima* L.	–	7
*Cardiovascular*		
*Gentiana septemfida* Pall.	8	3
*Rosa spinosissima* L.	2	7
Muscle and skeletal		
*Urtica dioica* L.	21	23
*Equisetum arvense* L.	9	5
*Genitourinary*		
*Rosa canina* L.	9	–
*Plantago major* L.	1	7

**Fig. 6:**
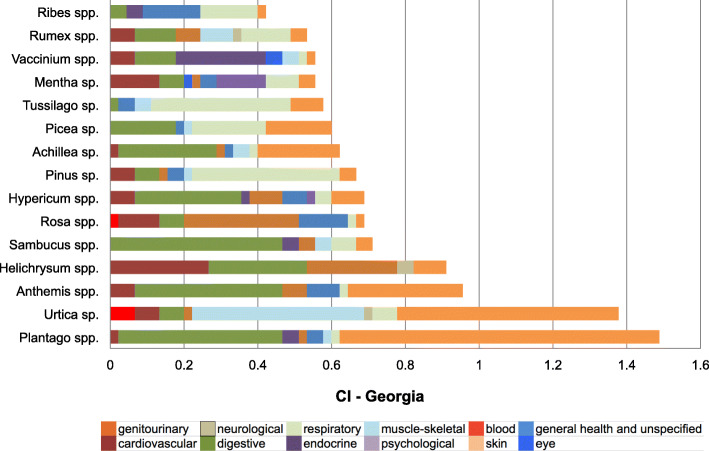
Cultural Importance (CI) index of top 15 genera in Georgia and their contribution to medicinal use categories. (spp. indicates contribution of more than one species, while sp. indicates the contribution of only one species)

**Fig. 7:**
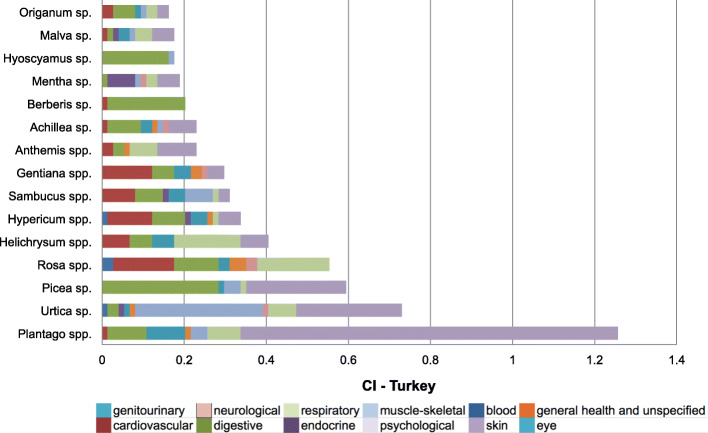
Cultural importance (CI) index of top 15 genera in Turkey and their contribution to medicinal use categories. (spp. indicates contribution of more than one species, while sp. indicates the contribution of only one species)

The 15 genera/species with highest CI and their relative importance in each medicinal use-category are shown in Figs. 6 and 7. It seems that the culturally most important species are also the most versatile species in terms of number of different uses. Based on CI index value, two-thirds of the top 15 genera in both countries have use reports for at least seven medicinal use categories. Therefore, the diversity of usage makes these species and genera of top priority for our participants’ health, well beings and cultures.

### Other medicinal use-categories

The categories with the fewest use reports (URs) in Georgia are psychological, neurological, blood, and eye complaints. Similar categories have the fewest reports in Turkey, namely neurological, blood, eye, psychological, and ear.

Drinking water infusions of *Tilia rubra* subsp. *caucasica* flowers is mentioned to be relaxing in Georgia. Regarding psychological complaints, tea of *Clinopodium grandiflorum*, *Mentha longifolia*, and *Hypericum perforatum*, seeds of *Papaver orientale* and flowers of *Tilia rubra* subsp. *caucasica* are mentioned for relaxing, sleep disorders, and as sedative by participants in Georgia. While in Turkey, *Echinops pungens* and *Papaver orientale* are mentioned for sleep disorders and as sedative. In the former, water infusion of leaves, while in the latter tea made of milk infused flowers is used.

As for neurological disorders, ten species are mentioned in each countries mostly for headache. Among these species, only *Urtica dioica* is shared by participants in both countries. Other than internal use of these species for headache as a tea, some species are applied externally as a headbands. *Petasites* spp. and *Rumex crispus* leaves are mentioned for this in Georgia while *Trachystemon orientalis* and *Urtica dioica* leaves are used in Turkey. Two previous unique reports from the Eastern Black Sea Region of Turkey also mention the external use of fresh *Rhododendron ponticum* leaves for headache [72, 73].

Considering blood-related problems, four species are given in Georgia. *Cornus mas* and *Urtica dioica* are used for anemia; *Rosa canina*, *Plantago major*, and *Urtica dioica* for cleansing the blood. All these species have also been reported for similar diseases in literature sources [14, 18, 76, 86]. On the other hand, seven species are named for blood problems in Turkey, six of which are used for anemia (Rosa spinosissima, Prunus avium, *Hypericum linarioides*, Urtica dioica, Prunus laurocerasus, Rosa canina), one for iron deficiency (Vaccinium myrtillus). A report of enhanced blood production of Vaccinium myrtillus and Prunus laurocerasus have also been mentioned in Turkey [71, 75].

For eye conditions, a drinking water infusion of Vaccinium myrtillus leaves and the eating of its fresh fruits are mentioned to be good for sight in Georgia. These remedies have also been reported from Georgia [25] and from Turkey [74]. External application of a water infusion of *Salix capraea* flower and a water infusion of Mentha longifolia aerial parts are reported to cure eye diseases in Georgia. On the other hand, in Turkey, a drinking water decoction of Crataegus rhipidophylla fruits and plastering *Arctium platylepis* leaves are mentioned for eye diseases. In addition, bathing with water decoction of *Teucrium polium* species aerial parts with flowers is reported for babies against hypopyon. Ear complaints are only mentioned in Turkey with two species. Plastering leaves of *Arctium platylepis* and drinking water infusion of *Tanacetum macrophyllum* flowers are reported to cure earache.

## Conclusions

The results of this study indicate that, due to combinations of high plant diversity, multicultural and multi-linguistic nature of the study area, the richness of traditional plant wisdom, unique knowledge, and depth of botanical understanding of people are reflected in the number of plant species they know, with their diverse folk plant names as well as methods of harvesting, preparing, and using these plants. Thus, this study area still maintains clear medicinal knowledge and practices regarding wild plants. Indeed, more than half of the distinct ethnomedicinal usage of wild plants documented in this study have not been reported in the Caucasus literature before. The majority of this plant knowledge is still present, partly in use or at least harbored in memories. However, especially in Turkey, many of the reported uses are no longer implemented in practice, only remaining in the memory of elders who still maintain their traditional agro-pastoral transhumant lifestyles.

What is striking is the fact that, despite environmental and floral similarity, common historical/cultural contact, and similar livelihood strategies, at the present time, shared ethnomedicinal knowledge/use across the border is unexpectedly low, forming less than 10% of the reported ethnomedicinal use incidences. It seems that patterns of medicinal knowledge in the study area are connected with multiple cultural factors, in particular ethnolinguistic diversity, cultural background, and access to multilingual written folk and scientific literature. These factors are shown to be variable among species. Due to its complex nature, while a generalized definite conclusion cannot be drawn, access to multilingual literary sources seems to be one of the most relevant driving forces to account for the medical plant knowledge patterns in the study area. To better understand the underlying factors and driving reasons for the shared and separate plant knowledge among different communities on both sides of this international border, future studies should consider cultural diversity (language, ethnicity), socio-economic conditions, as well as the political histories of each community. Restricting the comparison unit to a single plant genus, with structured questions may provide a more rigorous approach to the evaluation of patterns and dynamics of ethnobotanical knowledge. Most importantly, to identify factors that shape medicinal plant knowledge in such a multicultural area, strong collaboration between local people, botanists, ethnologists, ecologists, pharmacologists, linguists, anthropologists, and sociologists is essential for future research in the field of ethnobotany.

A number of practical implications related to medicinal wild plants have emerged from this study. Firstly, unique and shared plant species and their use knowledge documented in this study could encourage further phyto-pharmaceutical research for the development of natural botanical-based pharmaceuticals and phytotherapy practices. It is hoped that these will contribute to the health, well-being, and livelihood of these communities, in the Caucasus and worldwide. Secondly, Georgia’s National Biodiversity Strategy and Action Plan (2014–2020) has a recommendation for restoring traditional knowledge of local peoples related to biodiversity conservation and sustainable resource use, to be integrated into their legislation and national strategies by 2020 [87]. In Turkey, a recent Project of the Ministry of Forestry and Water Affairs has also taken a valuable step toward documenting biodiversity-based traditional knowledge [90]. As the links between traditional knowledge and the conservation of biodiversity receive increasing attention from the scientific community worldwide, ethnobotanical knowledge and practices related to plant resources in mountainous regions along the Georgia-Turkey border might contribute to future cross-border action plans and policies for plant conservation and management of vegetation resources. It is hoped this will add value to the development of functional models for biocultural diversity conservation, restoration, and sustainable uses of natural resources in the Caucasus.

## Supplementary information

**Additional file 1: Table S1.** Medicinal ethnobotany of reported wild plants in the study area.

## Data Availability

All data generated or analyzed during this study are included in this published article.

## Abbreviations

UR: Use-report
FIC: Informant consensus factor
CI: Cultural importance
ICPC: Index International Classification of Primary Care
