# Supplementary material for: Medicinal ethnobotany of wild plants: a cross-cultural comparison around Georgia-Turkey border, the Western Lesser Caucasus
Source: J Ethnobiol Ethnomed. 2020 Nov 23;16:71. doi: 10.1186/s13002-020-00415-y (PMC7681977; doi:10.1186/s13002-020-00415-y)
Supplement: Supplementary file 1 — Additional file 1: Table S1. Medicinal ethnobotany of reported wild plants in the study area. [file 13002_2020_415_MOESM1_ESM.docx]

Table 2 Medicinal ethnobotany of reported wild plants in the study area. (GEO: Georgia; TUR: Turkey; n: number of participants; UR: Use report; CI_s_: Cultural Importance Index of species)

| **Latin names of families and species (voucher or digital photograph number)^a^** | **Recorded local names^b^** | **Recorded plant part/s and processing techniques^c^** | **Medi-cinal use cate-gories^d^** | **Recorded medicinal subcategory and applications^e^** | **GEO  n=45   UR CIs** | | **TUR   n=74  UR CIs** | | **Same use in the literature^f^** | **Use in the  litera-ture^g^** |
| --- | --- | --- | --- | --- | --- | --- | --- | --- | --- | --- |
| ADOXACEAE |  |  |  |  |  |  |  |  |  |  |
| *Sambucus ebulus* L.  **(FP-SO 19, 20*)**  (CK, SO 361*, 419*, 1452, 1502) | antsli (ანწლი), tkis antsli; *antzli, aslı* | 1 fruits, fresh  2 fruits, infusion in water, keep under sun  3 fruits, infusion in alcohol at sunny place  4 fruits, infusion in water with sugar (1:1), leave it dark place  5 fruits, decoction in water  6 fruits, juice  7 fruits, sweet dish (jam)  8 fruits, syrup  9 fuits, wait 3 days in open cold air  10 leaves, warmed on stove  11 leaves, fresh  12 aerial parts, infusion in water  13 aerial parts, decoction in water  14 leaves, decoction in water  15 leaves, burned  16 roots, decoction in water | digest | jaundice *INT- D 12* | 16 | 0,60 | 4 | 0,28 | NO | x |
|  |  |  |  | constipation **INT- E 1**, 7, **D** *2*, 3, **4**, 6 |  |  |  |  | NO |  |
|  |  |  |  | worm *INT- D 2* |  |  |  |  | NO |  |
|  |  |  |  | dysentery INT- D 2 |  |  |  |  | NO |  |
|  |  |  |  | stomach ache INT- D 2, 3, E 1 |  |  |  |  | (26), (22), (15) |  |
|  |  |  |  | diarrhea INT- E 1, 2 |  |  |  |  | (1), (24), (26) |  |
|  |  |  | endoc | diabetes **INT***- D 9,* E 1 | 2 |  | 1 |  | NO | x |
|  |  |  | respir | cough *INT- E 8* | 3 |  | 1 |  | (24) | x |
|  |  |  |  | cold INT- E 1 |  |  |  |  | (24) |  |
|  |  |  |  | throat ache INT- E 1 |  |  |  |  | NO |  |
|  |  |  |  | influenza INT- D 3 |  |  |  |  | NO |  |
|  |  |  | skin | wart *EXT- F 15* | 2 |  | 2 |  | NO | x |
|  |  |  |  | itching *EXT- B 13* |  |  |  |  | NO |  |
|  |  |  |  | bruises EXT- P 10 |  |  |  |  | (26) |  |
|  |  |  | mu-sk | rheumatism **EXT***- B 14,* P 1, 10 | 2 |  | 5 |  | (2), (26), (19) | x |
|  |  |  |  | bone and joint pain *EXT- B 12* |  |  |  |  | NO |  |
|  |  |  |  | back pain *EXT- P 14* |  |  |  |  | NO |  |
|  |  |  |  | knee ache *EXT- P 14* |  |  |  |  | NO |  |
|  |  |  | genito | abortion *INT- D 6* | 2 |  | 2 |  | NO | x |
|  |  |  |  | woman disease *INT- E 1* |  |  |  |  | NO |  |
|  |  |  |  | kidney pain INT- E 1 |  |  |  |  | NO |  |
|  |  |  |  | urinary disease INT- E 1 |  |  |  |  | (26) |  |
|  |  |  | cardio | hemorrhoids *INT- E 1; EXT- B 5, 16* | 0 |  | 6 |  | (12), (26), (22) | x |
| *Sambucus nigra* L.  **(CK, SO 237*, 277*)**  (CK, SO 798) | didgula (დიდგულ), antzli; *antzli* | 1 fruits, 1kg fruit decocted in sugar, every morning drink on an empty stomach  2 aerial parts with fruits, decoction in water  3 flowers, infusion in water  4 fruits, infusion in sugar (1:1), insolation, one table spoon | digest | stomach ache INT- D 1; *EXT- B 2* | 5 | 0,11 | 1 | 0,03 | NO | x |
|  |  |  |  |  |  |  |  |  |  |  |
|  |  |  |  |  |  |  |  |  |  |  |
|  |  |  |  |  |  |  |  |  |  |  |
|  |  |  |  |  |  |  |  |  |  |  |
|  |  |  |  |  |  |  |  |  |  |  |
|  |  |  |  | constipation INT- D 1 |  |  |  |  | (26) |  |
|  |  |  |  |  |  |  |  |  |  |  |
|  |  |  |  | diarrhea INT- D 4 |  |  |  |  | (26) |  |
|  |  |  |  | dysentery INT- D 4 |  |  |  |  | NO |  |
|  |  |  |  | gastrointestinal infection INT- D 3 |  |  |  |  | NO |  |
|  |  |  | genito | women disease (infertility)  *EXT- B 2* | 0 |  | 1 |  | NO | NO |
| *Viburnum lantana* L.  **(CK, SO 9*, 455)**  (CK, SO 512*, 595*, 1498, 1664, 1673, 1704) | uzani (უზანი), uznis chikiri; *germoşa, dendereşik* | 1 fruits, fresh  2 branches, decoction in water, infused for one night  3 seeds, fresh  4 fruits, infusion in water, mixed with Mentha sp. | cardio | hemorrhoids INT- E 1 | 2 | 0,07 | 0 | 0,01 | (7), (17) | x |
|  |  |  |  | heart disease INT- D 4 |  |  |  |  | NO |  |
|  |  |  | digest | diarrhea INT- D 2 | 1 |  | 1 |  | (16), (26) | x |
|  |  |  |  | constipation *INT- E 3* |  |  |  |  | NO |  |
| *Viburnum opulus* L.  **(CK, SO 449, 488)**  (CK, SO 40, 1456, 1629, 1665) | jahola,  (ჯახველა, jakhvela); cahoyle, zakule | 1 fruits, fresh | cardio | high blood pressure INT- E 1 | 1 | 0,02 | 0 | 0,03 | (3), (15),  (24), (25) | x |
|  |  |  | genito | kidney disease *INT- E 1* | 0 |  | 1 |  | (11) | x |
|  |  |  | endoc | diabetes *INT- E 1* | 0 |  | 1 |  | NO | NO |
| ANACARDIACEAE |  |  |  |  |  |  |  |  |  |  |
| *Cotinus coggygria* Scop.  (CK, SO 2) | trimli (თრიმლი); *tirimli* | 1 branches, decoction in water  2 leaves, dried and powdered | GHUS | measles EXT- B 1; INT- D 1 | 1 | 0,02 | 0 | 0,01 | NO | NO |
|  |  |  |  |  |  |  |  |  |  |  |
|  |  |  |  |  |  |  |  |  |  |  |
|  |  |  | skin | rash *EXT- EM 2* | 0 |  | 1 |  | NO | NO |
| APIACEAE |  |  |  |  |  |  |  |  |  |  |
| *Anthriscus sylvestris* (L.) Hoffm.  **(CK, SO 509)**  (CK, SO 754) | ghimi (ჭყიმი, ch’q’imi)*; kımı, kımi* | 1 aerial parts, pickle  2 young aerial parts, fresh | endoc | loss of appetite **INT- E** 1, *2* | 1 | 0,02 | 1 | 0,01 | NO | NO |
| *Conium maculatum* L.  **(CK, SO 505)**  (FP-SO 21, 22, 23) | boligolov (Болиголов) | 1 flowers, infusion in alcohol, 20 days in dark place, start with 1 drop, increase until 10 drop doses in 50 ml water  2 flowers, infusion in water | GHUS | cancer INT- D 1 | 1 | 0,04 | 0 | 0,00 | NO | NO |
|  |  |  | neuro | headache INT- D 2 | 1 |  | 0 |  | NO | NO |
| *Ferula orientalis* L.  (FP-SO 24) | *çançur, çakşur, çaşur* | 1 roots, fresh  2 young aerial parts, fresh | genito | man disease (infertility) *INT- E 1* | 0 | 0,00 | 1 | 0,04 | NO | x |
|  |  |  | digest | stomach ache  *INT- E 2* | 0 |  | 1 |  | (17) | x |
|  |  |  | cardio | high blood pressure  *INT- E 2* | 0 |  | 1 |  | (6) | x |
| *Heracleum* spp.  **(CK, SO 470)**  (CK, SO 1338) | ghrenchela*; kekro, kekre, kerkaç, gölisi, gülisi, telehaj* | 1 stems, fresh  2 young leaves, fresh | endoc | diabetes *INT- E 1* | 0 | 0,02 | 1 | 0,05 | NO | x |
|  |  |  | GHUS | pain killer  *INT- E 1* | 0 |  | 2 |  | NO | x |
|  |  |  |  | good for health *INT- E 1* |  |  |  |  | NO |  |
|  |  |  | respir | asthma *INT- E 1* | 0 |  | 1 |  | NO | NO |
|  |  |  | digest | constipation INT- E 2 | 1 |  | 0 |  | NO | x |
| ARALIACEAE |  |  |  |  |  |  |  |  |  |  |
| *Hedera colchica* (K.Koch) K.Koch  **(CK, SO 84)**  (FP-SO 12) | suro (სურო) | 1 flowers, infusion in water | genito | vaginal discharge INT- D 1 | 1 | 0,02 | 0 | 0,00 | NO | x |
|  |  |  |  |  |  |  |  |  |  |  |
|  |  |  |  |  |  |  |  |  |  |  |
|  |  |  |  |  |  |  |  |  |  |  |
|  |  |  |  |  |  |  |  |  |  |  |
|  |  |  |  |  |  |  |  |  |  |  |
| ASPLENIACEAE |  |  |  |  |  |  |  |  |  |  |
| *Asplenium septentrionale* (L.) Hoffm.  (FP-SO 25, 26) | *mayasıl otu, basur otu* | 1 aerial parts, decoction in water | cardio | hemorrhoids *EXT- B 1* | 0 | 0,00 | 1 | 0,01 | NO | NO |
| ASTERACEAE |  |  |  |  |  |  |  |  |  |  |
| *Achillea millefolium* L.  **(CK, SO 476, 495, 533)** (CK, SO 1341, 1548) | hazerantertik (Հազարատերևուկ, hazaraterevuk), melagunda,  parsmanduki (ფარსმანდუკი), tsyiacheletnik (тысячелистник), asipurdzela, kvavis kuda (კრავიკუდა)*; hırpkesti, mide ağrısı çayı, hırpkesen, kılıç otu, diş otu, boriye çivike, civanperçemi, kındır ilacı* | 1 aerial parts with flowers, decoction in water  2 aerial parts with flowers, infusion in water  3 aerial parts with flowers, dried and powdered  4 aerial parts with flowers, burned ash  5 aerial parts with flowers, warmed on stove  6 aerial parts with flowers, infusion in water with *kalendula* and *romashka*  7 flowers, decoction in water  8 flowers, infusion in water  9 leaves, smash and mix with butter  10 leaves, infusion in water | cardio | hemorrhoids EXT- B 8 | 1 | 0,62 | 2 | 0,23 | (10), (21) | x |
|  |  |  |  | heart disease *INT- D 8* |  |  |  |  | NO |  |
|  |  |  | digest | indigestion INT- D 8 | 12 |  | 6 |  | NO | x |
|  |  |  |  | worm INT- D 8 |  |  |  |  | NO |  |
|  |  |  |  | stomach ache **INT- D** 1, **2** |  |  |  |  | (5), (25), (16), (18), (9), (22) |  |
|  |  |  |  | liver disease INT- D 2 |  |  |  |  | (25) |  |
|  |  |  |  | toothache **EXT- G 1,** *8* |  |  |  |  | (6) |  |
|  |  |  |  | intestinal disease INT- D 2 |  |  |  |  | NO |  |
|  |  |  |  | diarrhea *INT- D 2, 7* |  |  |  |  | (15) |  |
|  |  |  | genito | abortion *INT- D 7* | 1 |  | 2 |  | NO | x |
|  |  |  |  | kidney disease *INT- D 8* |  |  |  |  | NO |  |
|  |  |  |  | kidney pain INT- D 1 |  |  |  |  | (18) |  |
|  |  |  | GHUS | pain killer **INT- D** *2*, 6 | 1 |  | 1 |  | (19) | x |
|  |  |  | mu-sk | bone and joint pain EXT- B 8 | 2 |  | 1 |  | NO | x |
|  |  |  |  | sprain EXT- P 5 |  |  |  |  | NO |  |
|  |  |  |  | rheumatism *INT- D 2* |  |  |  |  | (17) |  |
|  |  |  | neuro | headache *INT- D 2* | 0 |  | 1 |  | (17) | x |
|  |  |  | respir | influenza INT- D 6 | 1 |  | 0 |  | NO | x |
|  |  |  | skin | eczema EXT- P 2, B 1 | 10 |  | 5 |  | NO | x |
|  |  |  |  | antiseptic EXT- P 2 |  |  |  |  | NO |  |
|  |  |  |  | boil EXT- P 2 |  |  |  |  | NO |  |
|  |  |  |  | inflammation **EXT- B** 2, *8* |  |  |  |  | (1), (25) |  |
|  |  |  |  | bleeding wound EXT- P 2, 8 |  |  |  |  | (5), (16), (18), (9) |  |
|  |  |  |  | wound **EXT- P** *2, 3*, 8, 9, 10 |  |  |  |  | (13), (25), (16), (10), (18), (9), (15) |  |
|  |  |  |  |  |  |  |  |  |  |  |
|  |  |  |  | herpes *EXT- P 4* |  |  |  |  | NO |  |
| *Anthemis* spp.  **(CK, SO 27)**  (CK, SO 39) | gvirila (გვირილა), romashka (ромашка) papatya; *gvirila papatya* | 1 aerial parts with flowers, infusion in water  2 aerial parts with flowers, decoction in water  3 flowers, infusion in water  4 flowers, decoction in water (mix with mint)  5 flowers, infusion in water mix with stingnettle, immortal, maple leaves  6 flowers, mix with honey  7 flowers, fresh | digest | ulcer (mouth) EXT- G 2 | 18 | 0,96 | 2 | 0,23 | NO | x |
|  |  |  |  | tooth inflammation EXT- G 1, 3, 4 |  |  |  |  | NO |  |
|  |  |  |  | stomach ache **INT**- D 1, 3, *E 7* |  |  |  |  | (16), (20), (18) |  |
|  |  |  |  | indigestion INT- D 1 |  |  |  |  | NO |  |
|  |  |  |  | toothache **EXT- G** 1, 2, **3**, 4 |  |  |  |  | NO |  |
|  |  |  |  | diarrhea INT- D 3 |  |  |  |  | NO |  |
|  |  |  | GHUS | allergy EXT- B 2 | 4 |  | 1 |  | NO | x |
|  |  |  |  | feeling ill INT- D 3 |  |  |  |  | NO |  |
|  |  |  |  | pain killer INT- D 3; **EXT- B 4** |  |  |  |  | NO |  |
|  |  |  | skin | hair care complaints (hair wash, loss)  **EXT- B 1, 3**, *4*, 5 | 14 |  | 7 |  | (16) | x |
|  |  |  |  | skin care complaints EXT- B 1 |  |  |  |  | NO |  |
|  |  |  |  | antiseptic INT- D 3 |  |  |  |  | NO |  |
|  |  |  |  | boil EXT- P 3 |  |  |  |  | NO |  |
|  |  |  |  | inflammation EXT- B 3; *INT- D 3* |  |  |  |  | NO |  |
|  |  |  |  | irritation EXT- B 3 |  |  |  |  | NO |  |
|  |  |  | cardio | hemorrhoids **EXT- B 4,** *3* | 3 |  | 2 |  | NO | x |
|  |  |  | genito | vaginal discharge EXT- B 3 | 3 |  | 0 |  | NO | x |
|  |  |  |  | kidney stone INT- D 3 |  |  |  |  | (22) |  |
|  |  |  |  | kidney disease INT- D 3 |  |  |  |  | (20) |  |
|  |  |  | respir | influenza INT- D 3 | 1 |  | 5 |  | NO | x |
|  |  |  |  | bronchitis *INT- E 6* |  |  |  |  | NO |  |
|  |  |  |  | cough *INT- D 3* |  |  |  |  | (6), (16), (18), (22) |  |
|  |  |  |  | shortness of breath  *INT- D 3* |  |  |  |  | NO |  |
| *Arctium platylepis*(Boiss. & Balansa) Sosn. ex Grossh.  **(CK, SO 485)**  (CK, SO 1387) | hozikay, ghabalak,  lopukh (лопух), lapuh, şerepuk, ghalaghunta (ღალაღუნტა); *düğe tabanı, kabalak, huzik* | 1 aerial parts, decoction in water  2 flowers, decoction in water  3 leaves, fresh  4 leaves, infusion in water  5 roots, mixed with *oduvanchik* 6 roots, decoction in water  7 roots, decoction in sunflower oil  8 roots, fresh | digest | tooth inflammation EXT- G 1 | 1 | 0,27 | 0 | 0,07 | NO | x |
|  |  |  | genito | prostate INT- D 2 | 1 |  | 0 |  | NO | x |
|  |  |  | GHUS | allergy INT- D 2 | 1 |  | 0 |  | NO | x |
|  |  |  | skin | hair care complaints (hair wash, loss)  EXT- B 4, 7 | 3 |  | 0 |  | NO | x |
|  |  |  |  | irritation EXT- B 6 |  |  |  |  | NO |  |
|  |  |  | mu-sk | knee ache **EXT- P** 3, *4* | 5 |  | 3 |  | NO | x |
|  |  |  |  | bone and joint pain **EXT- P 3** |  |  |  |  | (16) |  |
|  |  |  |  | rheumatism EXT- P 3, 4; *INT- E 8* |  |  |  |  | NO |  |
|  |  |  | endoc | diabetes INT- E 5 | 1 |  | 0 |  | NO | x |
|  |  |  | ear | ear ache *EXT- P 4* | 0 |  | 1 |  | NO | NO |
|  |  |  | eye | eye diseases *EXT- P 4* | 0 |  | 1 |  | NO | NO |
| *Artemisia absinthium* L.  (CK, SO 502)  (CK, SO 1568) | abzinda (აბზინდა), anzinta (აბზინთა), pelin | 1 leaves, infusion in water  2 aerial parts with flowers, decoction in water  3 aerial parts with flowers, infusion in water  4 leaves, fresh | respir | nasal obstruction *INT- V 1* | 0 | 0,16 | 1 | 0,01 | NO | x |
|  |  |  | digest | tooth inflammation EXT- G 2 | 6 |  | 0 |  | NO | x |
|  |  |  |  | toothache EXT- G 3; INT- C 4 |  |  |  |  | NO |  |
|  |  |  |  | stomach ache INT- D 1 |  |  |  |  | (6), (17) |  |
|  |  |  |  | worm INT- D 1 |  |  |  |  | (11) |  |
|  |  |  |  | constipation INT- D 1 |  |  |  |  | NO |  |
|  |  |  | GHUS | feeling ill INT- D 3 | 1 |  | 0 |  | NO | x |
| *Artemisia vulgaris* L.  **(FP-SO 27, 28, 29)** | (მყრალა-ბალახი,mkrala-balakhi), mkrala balagha | 1 aerial parts with flowers, infusion in water | cardio | heart disease INT- D 1 | 1 | 0,02 | 0 | 0,00 | NO | NO |
| *Carduus* spp.  **(FP-SO 32, 33)**  (FP-SO 30, 31) | *diken* | 1 flowers, infusion in water  2 entire plant, infusion in water  3 aerial parts, juice | respir | cold *INT- D 1* | 0 | 0,02 | 1 | 0,03 | NO | NO |
|  |  |  | mu-sk | rheumatism *INT- D 2* | 0 |  | 1 |  | NO | NO |
|  |  |  | skin | hair care complaints (hair loss) EXT- B 3 | 1 |  | 0 |  | (6) | x |
| *Centaurea macrocephala* Muss.Puschk. ex Willd.  (FP-SO 34) | *molla, beşkhi, güney çiçeği* | 1 flowers, decoction in water | skin | wound *EXT- P 1* | 0 | 0,00 | 1 | 0,01 | NO | NO |
| *Cichorium intybus* L.  **(CK-SO 43*)** (FP-SO 35) | cikori (цикорий, cikorij); *marçkakaç* | 1 aerial parts with flowers, burned ash  2 aerial parts, infusion in water  3 flowers, infusion in water  4 roots, infusion in water  5 roots, decoction in water  6 roots, coffee | skin | wound *EXT- P 1* | 0 | 0,16 | 2 | 0,03 | (18), (26) | x |
|  |  |  | digest | diarrhea INT- D 2 | 2 |  | 0 |  | (5), (8), (17) | x |
|  |  |  |  | stomach ache INT- D 3 |  |  |  |  | NO |  |
|  |  |  | endoc | diabetes INT- D 4 | 1 |  | 0 |  | (24), (15), (26) | x |
|  |  |  | genito | kidney stone INT- D 5 | 1 |  | 0 |  | NO | x |
|  |  |  | cardio | heart disease INT- D 6 | 2 |  | 0 |  | NO | x |
|  |  |  |  | high blood pressure INT-D 2 |  |  |  |  | NO |  |
|  |  |  | respir | cough INT- D 2 | 1 |  | 0 |  | NO | NO |
| *Cirsium caucasicum* Petr.  **(CK, SO 187*,209*, 32, 59)**  (CK, SO 1440) | birka, nari (ნარი); *deve tabanı* | 1 entire plant, infusion in water | digest | intestinal disease *INT- D 1* | 0 | 0,04 | 1 | 0,01 | NO | NO |
|  |  |  | GHUS | measles EXT- B 1 | 1 |  | 0 |  | NO | NO |
|  |  |  | cardio | hemorrhoids EXT- B 1 | 1 |  | 0 |  | NO | NO |
| *Cirsium kosmelii*(Adams) Fisch. ex Hohen.  **(FP-SO 36)** | ekala, ekliani balakhi | 1 leaves, decoction in water | digest | toothache EXT- G 1 | 1 | 0,02 | 0 | 0,00 | NO | NO |
| *Cyanus triumfettii* (All.) Dostál ex Á.Löve & D.Löve  *syn:* *Centaurea triumfettii* All.  **(FP-SO 37)** |  | 1 leaves, fresh  2 flowers, infusion in water | skin | wound *EXT- P 1* | 0 | 0,02 | 1 | 0,01 | NO | NO |
|  |  |  | respir | cough INT- D 2 | 1 |  | 0 |  | NO | NO |
| *Echinops pungens* Trautv.  (CK, SO 1556) | *yağlı diken, çobuk* | 1 receptacles, fresh  2 leaves, infusion in water | endoc | diabetes *INT- E 1* | 0 | 0,00 | 1 | 0,03 | NO | NO |
|  |  |  | psych | sleep disorders *INT- D 2* | 0 |  | 1 |  | NO | NO |
| *Eryngium billardierei* F.Delaroche  (FP-SO 38) | *kelem, diken* | 1 leaves, decoction in milk  2 leaves, macerated | skin | wound *EXT- P 1* | 0 | 0,00 | 1 | 0,03 | (14), (17) | x |
|  |  |  | cardio | hemorrhoids *EXT- EM 2* | 0 |  | 1 |  | NO | NO |
| *Eryngium giganteum* M.Bieb  **(CK, SO 34*,486, 508)** | samkbila ekala | 1 young aerial parts, fresh, press raw on aching tooth  2 leaves, fresh  3 aerial parts, decoction in water | digest | toothache INT- C 1, 2 | 3 | 0,07 | 0 | 0,00 | NO | NO |
|  |  |  |  | diarrhea INT- D 3 |  |  |  |  | NO |  |
| *Helichrysum plicatum* DC.  **(CK, SO 230*)** (FP-SO 39) | minduris nego, chaobis nego, antaram (անթառամ), nego(ნეგო), ukvdava (უკვდავა), besmertnik (бессмертник); *mayasir otu, pıltan, mayasır otu, sarı çiçek, nigo* | 1 aerial parts with flowers, infusion in water  2 aerial parts with flowers, decoction in water  3 aerial parts with flowers, decoction in milk  4 flowers, infusion in water | cardio | high blood pressure INT- D 1, 4 | 6 | 0,42 | 4 | 0,12 | NO | x |
|  |  |  |  | heart disease INT- D 1 |  |  |  |  | NO |  |
|  |  |  |  | hemorrhoids *INT- D 1; EXT B 1, 2* |  |  |  |  | NO |  |
|  |  |  | digest | liver disease INT- D 1; EXT- B 1 | 5 |  | 1 |  | NO | x |
|  |  |  |  | stomach ache INT- D 1 |  |  |  |  | NO |  |
|  |  |  |  | diarrhea INT- D 1 |  |  |  |  | NO |  |
|  |  |  |  | jaundice *INT- D 2* |  |  |  |  | (18) |  |
|  |  |  | genito | kidney disease **INT- D 1**, 4 | 4 |  | 1 |  | (5) | x |
|  |  |  |  | kidney stone INT- D 3 |  |  |  |  | (16), (10), (18), (14), (9), (22) |  |
|  |  |  | neuro | headache INT- D 1 | 2 |  | 0 |  | NO | NO |
|  |  |  | skin | hair care complaints (growth, loss)  **EXT- B 1**, 2 | 2 |  | 1 |  | NO | x |
|  |  |  | respir | cough *INT- D 1, 2* | 0 |  | 2 |  | (16) | x |
| *Helichrysum rubicundum* (K.Koch) Bornm.  **(CK, SO 19*, 30, 66, 472)** (CK, SO 1550, 1764) | nego (ნეგო), ukvdava (უკვდავა); *nego, sülenay, sulenay,  altın otu, sarı çiçek, sürvila, altın çiçek* | 1 aerial parts with flowers, infusion in water  2 aerial parts with flowers, decoction in water  3 entire plant, infusion in water  4 root, infusion in water  5 aerial parts with flowers, infusion in water, leave it dark place, after 4-5 day later drink it | genito | kidney disease **INT- D** 1, *2*, 3 | 7 | 0,49 | 3 | 0,28 | NO | x |
|  |  |  |  | women disease (infertility) INT- D 1; *EXT- B 2* |  |  |  |  | NO |  |
|  |  |  |  | kidney pain INT- D 1 |  |  |  |  | (16), (26), (8), (18) |  |
|  |  |  |  | prostate *INT- D 1* |  |  |  |  | NO |  |
|  |  |  | cardio | blood circulation INT- D 4 | 6 |  | 1 |  | NO | x |
|  |  |  |  | heart disease INT- D 1, 2, 5 |  |  |  |  | NO |  |
|  |  |  |  | hemorrhoids *EXT- B 1* |  |  |  |  | NO |  |
|  |  |  | skin | antiseptic EXT- B 4 | 2 |  | 4 |  | (26) | x |
|  |  |  |  | hair care complaints (hair wash)  **EXT- B 1**, *INT- D 2* |  |  |  |  | NO |  |
|  |  |  |  | psoriasis *EXT- B 2* |  |  |  |  | NO |  |
|  |  |  | digest | liver disease INT- D 1 | 7 |  | 3 |  | (1), (26) | x |
|  |  |  |  | stomach ache **INT- D 1** |  |  |  |  | (26) |  |
|  |  |  |  | jaundice *INT- D 2* |  |  |  |  | (18) |  |
|  |  |  | respir | throat inflammation *INT- D 1* | 0 |  | 10 |  | NO | x |
|  |  |  |  | throat ache *INT- D 1* |  |  |  |  | NO |  |
|  |  |  |  | cough *INT- D 1, 2* |  |  |  |  | (8) |  |
| *Petasites* spp.  **(FP-SO 41)**  (FP-SO 40) | barambo (ბარამბო), dilma (დილმა); *davut yaprağı* | 1 leaves, decoction in water  2 leaves, fresh | cardio | hemorrhoids EXT- B 1 | 1 | 0,04 | 0 | 0,01 | NO | NO |
|  |  |  | GHUS | fever *EXT- P 2* | 0 |  | 1 |  | NO | NO |
|  |  |  | neuro | headache *EXT- H 2* | 1 |  | 0 |  | NO | NO |
| *Senecio* sp.  **(FP-SO 42)** | khboshubla (ხბოშუბლა) | 1 roots, decoction in water | skin | irritation EXT- P 1 | 1 | 0,02 | 0 | 0,00 | NO | NO |
| *Tanacetum balsamita* L.  *syn:* *Pyrethrum balsamita* var. *tanacetoides* Boiss.  **(FP-SO 43)**  (FP-SO 44) | gvirila (გვირილა); *papatya* | 1 flowers, infusion in water  2 flowers, decoction in water  3 aerial parts, infusion in water, mix with thyme  4 leaves, decoction in water | digest | toothache EXT- G 2 | 3 | 0,16 | 1 | 0,08 | NO | NO |
|  |  |  |  | diarrhea INT- D 1 |  |  |  |  | NO |  |
|  |  |  |  | constipation *INT- D 1* |  |  |  |  | NO |  |
|  |  |  | GHUS | measles EXT- B 2 | 1 |  | 0 |  | NO | NO |
|  |  |  | respir | throat ache INT- D 1 | 1 |  | 1 |  | NO | NO |
|  |  |  |  | sinusitis *INT- D 2* |  |  |  |  | NO |  |
|  |  |  | skin | hair care complaints EXT- B 2 | 2 |  | 1 |  | NO | x |
|  |  |  |  | itching *EXT- B 1* |  |  |  |  | NO |  |
|  |  |  | cardio | hemorrhoids *EXT- B 4* | 0 |  | 1 |  | NO | NO |
|  |  |  | genito | vaginal discharge *INT- D 3* | 0 |  | 1 |  | NO | x |
|  |  |  | neuro | headache *INT- D 2* | 0 |  | 1 |  | NO | NO |
| *Tanacetum macrophyllum* (Waldst. & Kit.) Sch.Bip.  *syn:* *Pyrethrum macrophyllum* (Waldst. & Kit.) Willd.  **(CK, SO 37)**  (CK, SO 1028, 1446) | *sendel, papatya, sandal, acı papatya, sandal otu, sengel* | 1 leaves, warmed on stove  2 leaves, infusion in water | mu-sk | bone and joint pain EXT- P 1 | 1 | 0,02 | 0 | 0,08 | NO | NO |
|  |  |  | genito | women disease (inflammation) *INT- D 2* | 0 |  | 1 |  | NO | NO |
|  |  |  | respir | throat ache *INT- D 2* | 0 |  | 1 |  | NO | NO |
|  |  |  | ear | ear ache *INT- D 2* | 0 |  | 1 |  | NO | NO |
|  |  |  | skin | antiseptic *EXT- B 2* | 0 |  | 1 |  | NO | NO |
|  |  |  | GHUS | measles *INT- D 2* | 0 |  | 1 |  | NO | NO |
|  |  |  | endoc | loss of appetite *INT- D 2* | 0 |  | 1 |  | NO | NO |
| *Taraxacum* spp.  **(CK, SO 69, 545**)  (CK, SO 1289) | hadudik (Խատուտիկ, khatutik), oduvanchik (одуванчик), babuatsvera (ბაბუაწვერა); *ezegıze, kaz ayağı, ezeze* | 1 roots, infusion in water  2 roots, decoction in milk  3 flowers, infusion in water  4 flowers, infuson in votka at dark place  5 entire plant, decoction in water  6 entire plant, infusion in votka  7 sap, infusion in water  8 leaves, fresh  9 leaves, infusion in water | endoc | diabetes INT- D 6, C 8 | 3 | 0,31 | 0 | 0,05 | (6), (20), (17) | x |
|  |  |  |  | thyroid INT- E 8 |  |  |  |  | NO |  |
|  |  |  | digest | stomach ache INT- D 5, E 8 | 4 |  | 0 |  | (16) | x |
|  |  |  |  | liver disease INT- D 5 |  |  |  |  | (25) |  |
|  |  |  |  | constipation INT- D 1 |  |  |  |  | NO |  |
|  |  |  | respir | lung disease INT- D 5 | 1 |  | 0 |  | (8) | x |
|  |  |  | skin | hair care complaints (hair loss)  INT- D 5 | 1 |  | 4 |  | NO | x |
|  |  |  |  | wound *EXT- P 2, B 7* |  |  |  |  | (16), (9) |  |
|  |  |  |  | chap *EXT- B 7* |  |  |  |  | NO |  |
|  |  |  |  | callus *EXT- B 3* |  |  |  |  | NO |  |
|  |  |  | GHUS | pain killer INT- D 4 | 1 |  | 0 |  | NO | NO |
|  |  |  | mu-sk | back pain EXT- EM 4 | 4 |  | 0 |  | NO | x |
|  |  |  |  | bone and joint pain EXT- EM 4, 6;  INT D 1 |  |  |  |  | NO |  |
| *Tussilago farfara*L.  **(FP-SO 45)**  (FP-SO 46) | viris terpa (ვირისტერფა), dilma,  mati macheha (мать-и-мачеха), pirbamba (პირბამბა),  tetrigula bulgho,  anaanalık, porikuda, forikuda, gulbamba (გულბამბა),  dedinasveli, bamba balaghi (ბამბა-ბალახი),  borombo, barambu*; bir yüzlü yaprak, dilma,  pirbamba, iki yüzlü yaprak* | 1 leaves, infusion in water  2 leaves, decoction in water  3 leaves, fresh  4 leaves, macerated  5 leaves, infusion in water with *nego, pitna, bamba balakhi, gvirila* | digest | stomach ache INT- D 1 | 1 | 0,58 | 0 | 0,07 | NO | x |
|  |  |  | GHUS | tuberculosis INT- D 1 | 2 |  | 0 |  | NO | NO |
|  |  |  |  | fever EXT- P 3 |  |  |  |  | NO |  |
|  |  |  | mu-sk | rachitic EXT- P 3, B 2 | 2 |  | 0 |  | NO | NO |
|  |  |  | respir | cough INT- D 1, 2 | 17 |  | 0 |  | (1), (15), (17), (21), (24), (25) | x |
|  |  |  |  | bronchitis INT- D 1, 5 |  |  |  |  | (25), (21), (23) |  |
|  |  |  |  | asthma INT- D 1 |  |  |  |  | (21) |  |
|  |  |  |  | lung disease INT- D 1 |  |  |  |  | (25) |  |
|  |  |  | skin | wound **EXT- P** *1*, **3** | 4 |  | 5 |  | (15) | x |
|  |  |  |  | inflammation EXT- EM 4 |  |  |  |  | (15) |  |
|  |  |  |  | antiseptic EXT- EM 2 |  |  |  |  | NO |  |
|  |  |  |  | boil EXT- P 3 |  |  |  |  | NO |  |
| BERBERIDACEAE |  |  |  |  |  |  |  |  |  |  |
| *Berberis vulgaris* L.  **(CK, SO 27*, 52*, 458, 500)**  (CK, SO 56, 1651, 1693) | kotchahuri (კოწახური, k’ots’akhuri), shauna, barbarisi, (барбарис, barbaris) ; bınav, karmuk, çekehur, çıkahor, sakvitlo, karasorghun, sarılık otu, kasahor, koçahur, kotçahura | 1 leaves, infusion in water  2 branches, decoction in water  3 roots, decoction in water  4 fruits, decoction in water  5 branches with leaves, decoction in water  6 branches, decoction in three liter of water, mixed with one branch of Helichrysum sp. (nego) | digest | jaundice **EXT- B 2**, 6, 3, 4;  **INT- D** 6, 3, *2, 5* | 8 | 0,22 | 14 | 0,20 | (7), (8), (9), (13), (26) | x |
|  |  |  |  |  |  |  |  |  |  |  |
|  |  |  |  |  |  |  |  |  |  |  |
|  |  |  |  |  |  |  |  |  |  |  |
|  |  |  |  |  |  |  |  |  |  |  |
|  |  |  |  |  |  |  |  |  |  |  |
|  |  |  |  |  |  |  |  |  |  |  |
|  |  |  |  |  |  |  |  |  |  |  |
|  |  |  |  |  |  |  |  |  |  |  |
|  |  |  |  |  |  |  |  |  |  |  |
|  |  |  |  |  |  |  |  |  |  |  |
|  |  |  |  |  |  |  |  |  |  |  |
|  |  |  |  |  |  |  |  |  |  |  |
|  |  |  |  |  |  |  |  |  |  |  |
|  |  |  |  |  |  |  |  |  |  |  |
|  |  |  |  | liver disease INT- D 1, 3 |  |  |  |  | (25), (26) |  |
|  |  |  | cardio | hemorrhoids *EXT- B 2* | 1 |  | 1 |  | (8), (9) | x |
|  |  |  |  | high blood pressure INT- D 1 |  |  |  |  | (24), (26) |  |
|  |  |  |  |  |  |  |  |  |  |  |
|  |  |  | respir | throat ache INT- D 4 | 1 |  | 0 |  | NO | x |
| BETULACEAE |  |  |  |  |  |  |  |  |  |  |
| *Alnus glutinosa*subsp*. barbata* (C.A.Mey.) Yalt. *syn: Alnus barbata*C.A.Mey.  **(CK, SO 238*, 256*, 106)**  (CK, SO 328*, 16, 862, 1051) | tkhmela (თხმელა)*; kızılağaç,  bedzghi* | 1 fruits, infusion in water  2 leaves, infusion in water, mixed with hazelnut (Corylus sp.) leaves  3 pollen cones, infusion in water  4 leaf buds, macerated  5 leaves, fresh, how many wart you have hang in house that much leaves | genito | prostate INT- D 1 | 1 | 0,07 | 0 | 0,04 | NO | NO |
|  |  |  |  |  |  |  |  |  |  |  |
|  |  |  |  |  |  |  |  |  |  |  |
|  |  |  |  |  |  |  |  |  |  |  |
|  |  |  |  |  |  |  |  |  |  |  |
|  |  |  |  |  |  |  |  |  |  |  |
|  |  |  |  |  |  |  |  |  |  |  |
|  |  |  |  |  |  |  |  |  |  |  |
|  |  |  |  |  |  |  |  |  |  |  |
|  |  |  |  |  |  |  |  |  |  |  |
|  |  |  |  |  |  |  |  |  |  |  |
|  |  |  |  |  |  |  |  |  |  |  |
|  |  |  | skin | bruises EXT- B 2 | 1 |  | 2 |  | (4) | x |
|  |  |  |  | bleeding wound  *EXT- P 4* |  |  |  |  | (3), (8), (10), (11), (15) |  |
|  |  |  |  | wart *EXT*- *RT 5* |  |  |  |  | NO |  |
|  |  |  | respir | lung disease INT- D 3 | 1 |  | 0 |  | NO | NO |
|  |  |  | mu-sk | knee ache *EXT- P 4* | 0 |  | 1 |  | NO | x |
| *Betula pendula* Roth.  **(CK, SO 440)**  (CK, SO 22, 1536, 1590, 1643, 1659, 1695) | arki (არყი, ark’i), arkhi, beryoza (Берёза, bereza); *kayın* | 1 inner barks, decoction in water  2 leaves, infusion in water  3 inner barks, cut in pieces and roasted in butter | digest | tooth bleaching EXT- G 1 | 3 | 0,09 | 1 | 0,03 | NO | NO |
|  |  |  |  |  |  |  |  |  |  |  |
|  |  |  |  |  |  |  |  |  |  |  |
|  |  |  |  |  |  |  |  |  |  |  |
|  |  |  |  |  |  |  |  |  |  |  |
|  |  |  |  |  |  |  |  |  |  |  |
|  |  |  |  |  |  |  |  |  |  |  |
|  |  |  |  |  |  |  |  |  |  |  |
|  |  |  |  |  |  |  |  |  |  |  |
|  |  |  |  |  |  |  |  |  |  |  |
|  |  |  |  | gastrointestinal infection INT- D 1 |  |  |  |  | NO |  |
|  |  |  |  |  |  |  |  |  |  |  |
|  |  |  |  | stomach ache *INT- C 3* |  |  |  |  | NO |  |
|  |  |  |  | liver disease INT- D 2 |  |  |  |  | NO |  |
|  |  |  | genito | urinary disease INT- D 2 | 1 |  | 0 |  | NO | NO |
|  |  |  | respir | throat ache *INT- C 3* | 0 |  | 1 |  | NO | NO |
| *Corylus avellana* L.  **(CK, SO 11*, 248*, 442, 480)**  (CK, SO 778, 784, 810, 1627) | tkhili (თხილი); *fındık, tıkili* | 1 leaves, infusion in water  2 leaves, juice  3 leaves, fresh | skin | bruises EXT- B 1 | 1 | 0,07 | 2 | 0,03 | NO | x |
|  |  |  |  |  |  |  |  |  |  |  |
|  |  |  |  |  |  |  |  |  |  |  |
|  |  |  |  |  |  |  |  |  |  |  |
|  |  |  |  |  |  |  |  |  |  |  |
|  |  |  |  |  |  |  |  |  |  |  |
|  |  |  |  |  |  |  |  |  |  |  |
|  |  |  |  |  |  |  |  |  |  |  |
|  |  |  |  |  |  |  |  |  |  |  |
|  |  |  |  |  |  |  |  |  |  |  |
|  |  |  |  |  |  |  |  |  |  |  |
|  |  |  |  |  |  |  |  |  |  |  |
|  |  |  |  | bleeding wound *EXT- P 3* |  |  |  |  | NO |  |
|  |  |  |  | snake bite *INT- D 2* |  |  |  |  | NO |  |
|  |  |  | endoc | diabetes EXT - B 1 | 1 |  | 0 |  | NO | x |
|  |  |  | GHUS | allergy EXT- B 1 | 1 |  | 0 |  | NO | x |
| *Ostrya carpinifolia* Scop.  **(FP-SO 6)**  (CK, SO 778, 784, 810, 1627) | (უხრავი, ukhravi), ukraven, tudul | 1 flowers, infusion in water | neuro | headache INT- D 1 | 1 | 0,02 | 0 | 0,00 | NO | NO |
|  |  |  |  |  |  |  |  |  |  |  |
|  |  |  |  |  |  |  |  |  |  |  |
|  |  |  |  |  |  |  |  |  |  |  |
|  |  |  |  |  |  |  |  |  |  |  |
|  |  |  |  |  |  |  |  |  |  |  |
|  |  |  |  |  |  |  |  |  |  |  |
|  |  |  |  |  |  |  |  |  |  |  |
| BORAGINACEAE |  |  |  |  |  |  |  |  |  |  |
| *Symphytum asperum* Lepech.  **(CK, SO 474)** | shavakos, okopnik (окопник), saro (სარო) | 1 roots, infusion in pig fat, votka, and resin of *sosna*  2 roots, infusion in alcohol  3 roots, smashed and macerated  4 roots, infusion in water  5 roots, fresh  6 leaves, fresh | mu-sk | bone and joint pain EXT- P 4, 6 | 6 | 0,18 | 0 | 0,00 | (26) | x |
|  |  |  |  | sprain EXT- P 6 |  |  |  |  | NO |  |
|  |  |  |  | fracture EXT- P 1, 2 |  |  |  |  | (24), (25), (26) |  |
|  |  |  |  | rheumatism EXT- P 3 |  |  |  |  | NO |  |
|  |  |  | skin | bruises EXT- P 5 | 2 |  | 0 |  | NO | x |
|  |  |  |  | wound EXT- P 5 |  |  |  |  | (26) |  |
| *Trachystemon orientalis*(L.) D.Don   (FP-SO 47) | *bulğo, burgo* | 1 leaves, fresh | neuro | headache *EXT- H 1* | 0 | 0,00 | 1 | 0,03 | NO | NO |
|  |  |  | mu-sk | knee ache *EXT- P 1* | 0 |  | 1 |  | NO | NO |
| BRASSICACEAE |  |  |  |  |  |  |  |  |  |  |
| *Capsella bursa-pastoris* (L.) Medik.  **(CK, SO 538)**  (CK, SO 289) | pastushya sumka (пастушья сумка); *selmasık, ğıcı, dere maydanozu, piçok* | 1 leaves, infusion in water  2 leaves, fresh  3 aerial parts, mixed with *Sedum* sp. | genito | woman disease *INT- D 1* | 0 | 0,07 | 1 | 0,03 | NO | x |
|  |  |  | skin | inflammation *INT- D 1* | 2 |  | 1 |  | NO | x |
|  |  |  |  | bleeding wound EXT- P 2 |  |  |  |  | (16) |  |
|  |  |  |  | wound EXT- P 2 |  |  |  |  | (16) |  |
|  |  |  | GHUS | chickenpox EXT- P 3 | 1 |  | 0 |  | NO | x |
| *Cardamine hirsuta* L.  (FP-SO 48) | *sukubiyi* | 1 young aerial parts, fresh | cardio | hemorrhoids *INT- E 1* | 0 | 0,00 | 1 | 0,01 | NO | NO |
| *Sinapis arvensis* L*.* (FP-SO 49) | *tita* | 1 leaves, fresh | digest | stomach ache *INT- E 1* | 0 | 0,00 | 2 | 0,04 | NO | NO |
|  |  |  |  | worm  *INT- E 1* |  |  |  |  | NO |  |
|  |  |  | cardio | hemorrhoids *INT- E 1* | 0 |  | 1 |  | NO | NO |
| CAPRIFOLIACEAE |  |  |  |  |  |  |  |  |  |  |
| *Valeriana officinalis* L.  **(FP-SO 50)** | valeriya (валерьяна, valeryana) | 1 roots, infusion in water | cardio | high blood pressure INT- D 1 | 1 | 0,02 | 0 | 0,00 | NO | x |
| *Lonicera xylosteum* L.  (FP-SO 1) | *avi kakası, dindiger* | 1 fruits, fresh | digest | stomach ache *INT- E 1* | 0 | 0,00 | 1 | 0.01 | NO | NO |
| CORNACEAE |  |  |  |  |  |  |  |  |  |  |
| *Cornus mas* L.  **(FP-SO 7)**  (CK, SO 413*, 1797) | shindi (შინდი); *kızılcık* | 1 fruits, fresh  2 roots, decoction in water  3 fruits, sweet dish (jam) | blood | anemia INT- E 1 | 1 | 0,04 | 0 | 0,03 | (26) | x |
|  |  |  |  |  |  |  |  |  |  |  |
|  |  |  |  |  |  |  |  |  |  |  |
|  |  |  |  |  |  |  |  |  |  |  |
|  |  |  |  |  |  |  |  |  |  |  |
|  |  |  |  |  |  |  |  |  |  |  |
|  |  |  |  |  |  |  |  |  |  |  |
|  |  |  |  |  |  |  |  |  |  |  |
|  |  |  | digest | diarrhoea *INT- E 1* | 0 |  | 2 |  | (13), (26) | x |
|  |  |  |  | toothache *INT- V 2* |  |  |  |  | NO |  |
|  |  |  | cardio | heart disease INT- E 2 | 1 |  | 0 |  | NO | NO |
| CRASSULACEAE |  |  |  |  |  |  |  |  |  |  |
| *Sempervivum caucasicum* Rupr. ex Boiss.  **(FP-SO 51)** | kldisduma (კლდისდუმა) | 1 aerial parts, fresh  2 aerial parts, macerated | neuro | epilepsy INT- E 1 | 1 | 0,04 | 0 | 0,00 | NO | NO |
|  |  |  | skin | bleeding wound EXT- P 2 | 1 |  | 0 |  | (15) | x |
| *Sedum* spp.  **(FP-SO 52)** | tknikura (თიკნიყურა), kldis vardi | 1 aerial parts, macerated  2 aerial parts, mixed with *Capsella bursa pastoris* | cardio | hemorrhoids EXT- P 1 | 1 | 0,07 | 0 | 0,00 | NO | NO |
|  |  |  | GHUS | chickenpox EXT- P 2 | 1 |  | 0 |  | NO | x |
|  |  |  | skin | wound EXT- P 1 | 1 |  | 0 |  | (1), (12), (17), (25), (16), (26) | x |
| CUPRESSACEAE |  |  |  |  |  |  |  |  |  |  |
| *Juniperus  communis* L.  syn: *Juniperus  hemisphaerica*  C.Presl  **(CK, SO 178*, 511)**  (CK, SO 382, 439*, 614*, 1026, 1063, 1670) | *ardıç, arduç,* | 1 fruits, infusion in water  2 fruits, sweet dish (pekhmez)  3 fruits, decoction in water | respir | asthma *INT- D 1* | 0 | 0,00 | 1 | 0.04 | NO | x |
|  |  |  | digest | ulcer *INT- E 2* | 0 |  | 1 |  | NO | x |
|  |  |  | cardio | haemorrhoids *INT- D 3* | 0 |  | 1 |  | (7) | x |
| DENNSTAEDTIACEAE |  |  |  |  |  |  |  |  |  |  |
| *Pteridium tauricum* V.I. Krecz.  **(FP-SO 53)** | tabela | 1 aerial parts, infusion in water  2 leaves, fresh | mu-sk | bone and joint pain EXT- P 1 | 1 | 0,07 | 0 | 0,00 | NO | NO |
|  |  |  | genito | kidney pain EXT- P 2 | 2 |  | 0 |  | NO | NO |
|  |  |  |  | women disease (infertility) EXT- P 2 |  |  |  |  | NO |  |
| DIPSACACEAE |  |  |  |  |  |  |  |  |  |  |
| *Cephalaria gigantea*(Ledeb.) Bobrov  (CK, SO 1015,1494) | *sancı otu, sancı çiçeği, paliya yaprağı, poliyo  kanteper, gülhatın, polye* | 1 aerial parts with flowers, infusion in water  2 flowers, infusion in water  3 flowers, decoction in water  4 flowers, mix with Verbascum sp.  5 leaves, decoction in water  6 roots, decoction in water  7 seeds, dried | genito | women disease (infertility) *INT- D 1* | 0 | 0,00 | 5 | 0,15 | NO | x |
|  |  |  |  | menstruation problems *INT- D 1* |  |  |  |  | (6) |  |
|  |  |  |  | abortion *INT- E 4* |  |  |  |  | NO |  |
|  |  |  |  | women disease (inflammation) *INT- D 2* |  |  |  |  | NO |  |
|  |  |  |  | kidney stone *INT- D 3* |  |  |  |  | NO |  |
|  |  |  | skin | antiseptic *EXT- P 1* | 0 |  | 2 |  | NO | x |
|  |  |  |  | wound *EXT- P 7* |  |  |  |  | NO |  |
|  |  |  | digest | stomach ache *INT- D 2* | 0 |  | 2 |  | NO | x |
|  |  |  |  | jaundice *INT- D 2* |  |  |  |  | NO |  |
|  |  |  | respir | expectoration *INT- D 5, 6* | 0 |  | 2 |  | NO | x |
| DRYOPTERIDACEAE |  |  |  |  |  |  |  |  |  |  |
| *Dryopteris filix-mas* (L.) Schott  **(FP-SO 54)**  (FP-SO 55) | etseri, zirkbila; *ezer* | 1 leaves, burned ash  2 leaves, fresh  3 leaves, decoction in water  4 roots, decoction in water | skin | wart *EXT- P 1* | 0 | 0,09 | 1 | 0,01 | NO | x |
|  |  |  | genito | women disease (infertility) EXT- P 2 | 1 |  | 0 |  | NO | NO |
|  |  |  | respir | cough INT- D 4 | 2 |  | 0 |  | NO | NO |
|  |  |  |  | bronchitis INT- D 4 |  |  |  |  | NO |  |
|  |  |  | cardio | hemorrhoids EXT- B 3 | 1 |  | 0 |  | (26) | x |
| ELAEAGNACEAE |  |  |  |  |  |  |  |  |  |  |
| *Elaeagnus rhamnoides* (L.) A.Nelson  syn*: Hippophae rhamnoides* L.  **(CK, SO 443**)   (FP-SO 2) | aplipika (облепиха, oblepikha), | 1 fruits, juice | digest | stomach ache INT- D 1 | 1 | 0,02 | 0 | 0,00 | (12) | x |
|  |  |  |  |  |  |  |  |  |  |  |
|  |  |  |  |  |  |  |  |  |  |  |
|  |  |  |  |  |  |  |  |  |  |  |
|  |  |  |  |  |  |  |  |  |  |  |
|  |  |  |  |  |  |  |  |  |  |  |
|  |  |  |  |  |  |  |  |  |  |  |
|  |  |  |  |  |  |  |  |  |  |  |
|  |  |  |  |  |  |  |  |  |  |  |
| EQUISETACEAE |  |  |  |  |  |  |  |  |  |  |
| *Equisetum arvense* L.  **(CK-SO 101*, 202*)**  (FP-SO 56) | datvipanchari (დათვიფანჩარი), shvita (შვიტა), nadzva,  nadzvabalakha (ნაძვაბალახა),  datvapashva; *kırkkilit otu, doruk otu, at kuyruğu, piluncz, havsi, at kuyruğu* | 1 aerial parts, infusion in water  2 aerial parts, decoction in water | cardio | hemorrhoids **EXT- B 2** | 2 | 0,38 | 1 | 0,09 | (20) | x |
|  |  |  | genito | women disease (inflammation) EXT- B 2 | 5 |  | 1 |  | NO | x |
|  |  |  |  | kidney stone **INT- D 1** |  |  |  |  | (20) |  |
|  |  |  |  | kidney pain INT- D 1 |  |  |  |  | (1), (17) |  |
|  |  |  |  | prostate EXT- B 2 |  |  |  |  | NO |  |
|  |  |  | respir | lung disease INT- D 2 | 1 |  | 0 |  | NO | x |
|  |  |  | mu-sk | bone and joint pain **EXT- B 2;  INT- D** *1,* **2** | 9 |  | 5 |  | NO | x |
|  |  |  |  | rachitic EXT- B 1, 2 |  |  |  |  | NO |  |
|  |  |  |  | back pain EXT- B 2 |  |  |  |  | NO |  |
|  |  |  |  | rheumatism EXT- B 2 |  |  |  |  | (17) |  |
| ERICACEAE |  |  |  |  |  |  |  |  |  |  |
| *Rhododendron caucasicum* Pall*.* **(CK, SO 203*)**  (CK, SO 437) | deka, (დეკა, dek’a); *çekem, çekeme* | 1 leaves and roots, decoction in water  2 leaves, infusion in water  3 leaves, decoction in water | GHUS | general disease *INT- D 1* | 0 | 0,04 | 1 | 0,03 | NO | x |
|  |  |  |  |  |  |  |  |  |  |  |
|  |  |  |  |  |  |  |  |  |  |  |
|  |  |  |  |  |  |  |  |  |  |  |
|  |  |  |  |  |  |  |  |  |  |  |
|  |  |  |  |  |  |  |  |  |  |  |
|  |  |  |  |  |  |  |  |  |  |  |
|  |  |  |  |  |  |  |  |  |  |  |
|  |  |  |  |  |  |  |  |  |  |  |
|  |  |  |  |  |  |  |  |  |  |  |
|  |  |  |  |  |  |  |  |  |  |  |
|  |  |  |  |  |  |  |  |  |  |  |
|  |  |  |  |  |  |  |  |  |  |  |
|  |  |  |  |  |  |  |  |  |  |  |
|  |  |  | skin | itching *INT- D 2* | 0 |  | 1 |  | NO | x |
|  |  |  | endoc | diabetes INT- D 3 | 1 |  | 0 |  | NO | NO |
|  |  |  | respir | cough INT- D 3 | 1 |  | 0 |  | (25) | x |
| *Rhododendron luteum* Sweet  **(CK, SO 285*, 102)**  (CK, SO 528*, 634*, 747, 809, 1431, 1503, 1663) | yeli, (ieli,იელი); *yel* | 1 flowers, decoction in water  2 leaves, decoction in water | skin | irritation EXT- B 1 | 1 | 0,02 | 0 | 0,01 | NO | x |
|  |  |  |  |  |  |  |  |  |  |  |
|  |  |  |  |  |  |  |  |  |  |  |
|  |  |  |  |  |  |  |  |  |  |  |
|  |  |  |  |  |  |  |  |  |  |  |
|  |  |  | mu-sk | bone and joint pain *EXT- B 2* | 0 |  | 1 |  | NO | NO |
| *Rhododendron ponticum* L.  **(CK, SO 287*, 101, 107, 523)**  (CK, SO 387*, 573*, 1435, 1678) | shkeri (შქერი) | 1 leaves, decoction in water | skin | antiseptic EXT- B 1 | 1 | 0,02 | 0 | 0,00 | NO | x |
|  |  |  |  |  |  |  |  |  |  |  |
|  |  |  |  |  |  |  |  |  |  |  |
|  |  |  |  |  |  |  |  |  |  |  |
|  |  |  |  |  |  |  |  |  |  |  |
|  |  |  |  |  |  |  |  |  |  |  |
|  |  |  |  |  |  |  |  |  |  |  |
| *Vaccinium arctostaphylos* L.  **(CK, SO 58, 97, 531)**  (CK, SO 388*, 650, 665, 801, 1451, 1508, 1685) | maghali motsvi (მაღალი მოცვი), motsvi; *motsvi, mortsvi yaban mersini, likapa* | 1 leaves, infusion in water  2 fruits, fresh  3 young branches with leaves, decoction in water, mixed with (*Rubus* sp.) makvali leaves  4 young branches with leaves, infusion in water | endoc | diabetes INT- D 1, 4 | 4 | 0,24 | 0 | 0,03 | (15), (24), (25) | x |
|  |  |  |  |  |  |  |  |  |  |  |
|  |  |  |  |  |  |  |  |  |  |  |
|  |  |  |  | gout INT- D 1 |  |  |  |  | NO |  |
|  |  |  | cardio | high blood pressure INT- D 1 | 1 |  | 0 |  | NO | NO |
|  |  |  | digest | diarrhoea INT- E 2 | 2 |  | 0 |  | (15), (26) | x |
|  |  |  | mu-sk | bone and joint pain **INT- D 1,** E 2 | 2 |  | 1 |  | NO | NO |
|  |  |  | skin | eczema INT- D 3 | 1 |  | 0 |  | NO | NO |
|  |  |  | respir | influenza INT- E 2 | 1 |  | 0 |  | (24) | x |
|  |  |  | GHUS | general disease *INT- D 1* | 0 |  | 1 |  | NO | NO |
| *Vaccinium myrtillus* L.  **(CK, SO 231*, 520)**  (CK, SO 398*, 438*, 598*, 489, 517, 802, 1447, 1506) | motsvi (მოცვი), samkurnalo motsvi*; motsvi, motçi, mahabur* | 1 fruits, fresh  2 leaves, infusion in water  3 young branches with leaves, infusion in water | eye | eye diseases INT- D 3 | 2 | 0,27 | 0 | 0,05 | NO | x |
|  |  |  |  |  |  |  |  |  |  |  |
|  |  |  |  |  |  |  |  |  |  |  |
|  |  |  |  |  |  |  |  |  |  |  |
|  |  |  |  |  |  |  |  |  |  |  |
|  |  |  |  |  |  |  |  |  |  |  |
|  |  |  |  |  |  |  |  |  |  |  |
|  |  |  |  |  |  |  |  |  |  |  |
|  |  |  |  |  |  |  |  |  |  |  |
|  |  |  |  |  |  |  |  |  |  |  |
|  |  |  |  |  |  |  |  |  |  |  |
|  |  |  |  | good for eyes INT- E 1 |  |  |  |  | (3), (11) |  |
|  |  |  | GHUS | general disease *INT- D 2* | 0 |  | 1 |  | NO | x |
|  |  |  | endoc | diabetes **INT- E 1,** **D 2**, 3 | 6 |  | 2 |  | (2), (10), (11), (12), (21), (25) | x |
|  |  |  |  | gout INT- D 2 |  |  |  |  | (3) |  |
|  |  |  | digest | constipation INT- E 1 | 3 |  | 0 |  | (12), (11), (21) | x |
|  |  |  |  | indigestion INT- D 2 |  |  |  |  | (2), (11), (12) |  |
|  |  |  |  | diarrhoea INT- D 2 |  |  |  |  | NO |  |
|  |  |  | blood | iron deficiency *INT- D 2* | 0 |  | 1 |  | (12) | x |
|  |  |  | cardio | haemorrhoids EXT- B 3 | 1 |  | 0 |  | NO | NO |
| *Vaccinium uliginosum* L.  **(CK, SO 271*)** | motsvi (მოცვი) | 1 young branches with leaves, infusion in water  2 fruits, fresh | cardio | high blood pressure INT- D 1 | 1 | 0,04 | 0 | 0,00 | NO | NO |
|  |  |  | endoc | diabetes INT- E 2 | 1 |  | 0 |  | NO | NO |
| EUPHORBIACEAE |  |  |  |  |  |  |  |  |  |  |
| *Euphorbia* sp.  (FP-SO 57) | *sütlük, sütlü ot* | 1 sap 2 aerial parts, macerated | skin | wart *EXT- P 1, 2* | 0 | 0,00 | 2 | 0,03 | (8), (9), (10), (26) | x |
| FABACEAE |  |  |  |  |  |  |  |  |  |  |
| *Astracantha microcephala* (Willd.) Podlech  syn: *Astragalus microcephalus*Willd*.* **(CK, SO 479)**  (CK, SO 392*, 1583, 1713*)* | glerdzi (გლერძი); *geven, guni* | 1 roots, fresh  2 fruits, decoction in water  3 roots, burned  4 roots, decoction in water | digest | stomach ache EXT- EM 3 | 1 | 0,02 | 1 | 0,07 | NO | NO |
|  |  |  |  |  |  |  |  |  |  |  |
|  |  |  |  |  |  |  |  |  |  |  |
|  |  |  |  |  |  |  |  |  |  |  |
|  |  |  |  | jaundice *INT- D 4* |  |  |  |  | NO |  |
|  |  |  | skin | wound *EXT- P 1* | 0 |  | 2 |  | (8), (18) | x |
|  |  |  |  | psoriasis *INT- D 4* |  |  |  |  | NO |  |
|  |  |  | cardio | varicose vein *INT- D 2* | 0 |  | 1 |  | NO | NO |
|  |  |  | endoc | diabetes *INT- D 4* | 0 |  | 1 |  | NO | NO |
| *Trifolium alpestre* L.  **(FP-SO 58)**  (FP-SO 59) | klever (клевер), tsiteli samkura (წითელი სამყურა); *samkuri* | 1 flowers, infusion in water  2 leaves, fresh | endoc | sexual development (induce oestrogen level) INT- D 1 | 1 | 0,04 | 0 | 0,01 | NO | NO |
|  |  |  | cardio | varicose vein INT- D 1 | 1 |  | 0 |  | NO | NO |
|  |  |  | skin | wound *EXT-P 2* | 0 |  | 1 |  | NO | NO |
| *Trifolium ambiguum* M.Bieb.  (FP-SO 60) | klever (клевер)  yereknuk (Երեքնուկ),yonca | 1 flowers, infusion in water | endoc | sexual development (induce oestrogen level) INT- D 1 | 1 | 0,04 | 0 | 0,00 | (16) | x |
|  |  |  | digest | clean intestine INT- D 1 | 1 |  | 0 |  | NO | x |
| *Trifolium canescens*Willd*.* (FP-SO 61) | *samkuri* | 1 leaves, fresh | skin | wound *EXT- P 1* | 0 | 0,00 | 1 | 0,01 | NO | NO |
| *Trifolium pratense* L.  **(FP-SO 62)**  (FP-SO 63) | samkura (სამყურა), yereknuk (Երեքնուկ); *samkuri, samkuray* | 1 flowers, fresh  2 flowers, mix with honey and salt  3 flowers, infusion in water  4 leaves, fresh | skin | wart *EXT- P 1* | 0 | 0,04 | 2 | 0,04 | NO | x |
|  |  |  |  | wound *EXT- P 4* |  |  |  |  | (7), (13) |  |
|  |  |  | mu-sk | sprain *EXT- P 2* | 0 |  | 1 |  | NO | NO |
|  |  |  | cardio | high blood pressure INT- D 3 | 1 |  | 0 |  | NO | NO |
|  |  |  | genito | menstruation problems INT- D 3 | 1 |  | 0 |  | NO | NO |
| *Vicia villosa* Roth  (FP-SO 64) | *külül* | 1 fruits, infusion in water | respir | cough *INT- D 1* | 0 | 0,00 | 1 | 0,01 | NO | NO |
| FAGACEAE |  |  |  |  |  |  |  |  |  |  |
| *Quercus macranthera* Fisch. & C.A.Mey. ex Hohen.  **(CK, SO 438)**  (CK, SO 1796) | dub (Дуб) | 1 outer barks, decoction in water | respir | throat inflammation EXT- G 1 | 1 | 0,02 | 0 | 0,00 | NO | NO |
|  |  |  |  |  |  |  |  |  |  |  |
|  |  |  |  |  |  |  |  |  |  |  |
|  |  |  |  |  |  |  |  |  |  |  |
|  |  |  |  |  |  |  |  |  |  |  |
|  |  |  |  |  |  |  |  |  |  |  |
|  |  |  |  |  |  |  |  |  |  |  |
| *Quercus petraea*subsp. *iberica* (Steven ex M.Bieb.) Krassiln  syn*: Quercus  iberica*Steven ex M.Bieb.  **(CK, SO 126*, 259*)**  (CK, SO 389*, 434*, 525*, 555*, 565*, 633*, 785, 1046, 1049, 1057, 1487) | mukha (მუხა) | 1 outer barks, decoction in water, one tablespoon, in the morning  2 outer barks, infusion in water  3 outer barks, infusion in water, mixed with roots of *Urtica* sp. (chinchar) | digest | toothache EXT- G 1 | 11 | 0,27 | 0 | 0,00 | (26) | x |
|  |  |  |  |  |  |  |  |  |  |  |
|  |  |  |  |  |  |  |  |  |  |  |
|  |  |  |  |  |  |  |  |  |  |  |
|  |  |  |  |  |  |  |  |  |  |  |
|  |  |  |  |  |  |  |  |  |  |  |
|  |  |  |  |  |  |  |  |  |  |  |
|  |  |  |  | diarrhoea INT- D 1, 2 |  |  |  |  | (26) |  |
|  |  |  |  | tooth inflammation EXT- G 1 |  |  |  |  | (26) |  |
|  |  |  |  |  |  |  |  |  |  |  |
|  |  |  |  | gall disease INT- D 3 |  |  |  |  | NO |  |
|  |  |  |  | stomach ache INT- D 2 |  |  |  |  | NO |  |
|  |  |  |  |  |  |  |  |  |  |  |
|  |  |  | skin | hair care complaints (hair loss) INT- D 3 | 1 |  | 0 |  | NO | NO |
| GENTIANACEAE |  |  |  |  |  |  |  |  |  |  |
| *Gentiana asclepiadea* L.  **(CK, SO 64, 93, 530)** (FP-SO 65) | *asitava* | 1 aerial parts with flowers, decoction in water | cardio | hemorrhoids EXT- B 1 | 1 | 0,02 | 0 | 0,01 | NO | NO |
|  |  |  | GHUS | pain killer *INT- D 1* | 0 |  | 1 |  | NO | NO |
| *Gentiana cruciata* L.  **(CK, SO 471)** (CK, SO 832, 1014, 1555) | buasilis balakhi; *mayasir otu, asitava, mayasır otu, bağsır* | 1 aerial parts with flowers, decoction in water  2 aerial parts with flowers, infusion in water | cardio | hemorrhoids **EXT- B 1,** *2* | 1 | 0,02 | 5 | 0,12 | (9) | x |
|  |  |  | skin | antifungal *EXT- B 1* | 0 |  | 2 |  | NO | x |
|  |  |  |  | psoriasis *EXT- B 1* |  |  |  |  | NO |  |
|  |  |  | genito | vaginal discharge *EXT- B 1* | 0 |  | 1 |  | NO | x |
|  |  |  | digest | stomach ache *EXT- B 1* | 0 |  | 1 |  | (25) | x |
| *Gentiana gelida*M.Bieb.  **(FP-SO 66)**  (FP-SO 67) | lavanda*; mayasır otu, hırpkesen* | 1 aerial parts with flowers, decoction in water  2 aerial parts with flowers, infusion in water  3 flowers, infusion in water | genito | kidney disease EXT- B 3 | 1 | 0,02 | 2 | 0,11 | NO | NO |
|  |  |  |  | vaginal discharge *EXT- B 1* |  |  |  |  | NO |  |
|  |  |  | GHUS | pain killer *INT- D 2* | 0 |  | 1 |  | (5) | x |
|  |  |  | neuro | headache *INT-D 2* | 0 |  | 1 |  | NO | NO |
|  |  |  | skin | antifungal *EXT- B 1* | 0 |  | 2 |  | NO | NO |
|  |  |  | digest | stomach ache *EXT- B 1* | 0 |  | 2 |  | NO | NO |
|  |  |  |  | diarrhea *INT- D 2* |  |  |  |  | NO |  |
|  |  |  | cardio | hemorrhoids *EXT- B 1* | 0 |  | 1 |  | NO | NO |
| *Gentiana septemfida* Pall.  **(CK, SO 81, 526)** (CK, SO 720, 764) | buasilis balakhi,  mayasiris, pıkhana, asistava (ასისთავა)*; sancı otu mayasıl otu* | 1 aerial parts with flowers, decoction in water  2 aerial parts with flowers, infusion in water  3 flowers, infusion in water | genito | kidney disease EXT- B 3 | 1 | 0,29 | 0 | 0,05 | NO | NO |
|  |  |  | digest | induce vomiting INT- D 2 | 3 |  | 1 |  | NO | x |
|  |  |  |  | stomach ache **INT- D 2** |  |  |  |  | (25), (26) |  |
|  |  |  | skin | irritation EXT- B 2 | 1 |  | 0 |  | NO | NO |
|  |  |  | cardio | hemorrhoids **EXT- B 1, 2**; **INT- D 2**, T 2 | 8 |  | 3 |  | NO | NO |
| GROSSULARIACEAE |  |  |  |  |  |  |  |  |  |  |
| *Ribes alpinum* L.   **(CK, SO 457)**  (CK, SO 624*, 1618, 1701) | mertskhali (მერცხალი) tsiteli motskhari (მოცხარი), smorodina, (смородина,smorodina) | 1 fruits, fresh  2 leaves, infusion in water | endoc | diabetes INT- E 1 | 1 | 0,04 | 0 | 0,00 | NO | NO |
|  |  |  |  |  |  |  |  |  |  |  |
|  |  |  |  |  |  |  |  |  |  |  |
|  |  |  |  |  |  |  |  |  |  |  |
|  |  |  |  |  |  |  |  |  |  |  |
|  |  |  |  |  |  |  |  |  |  |  |
|  |  |  |  |  |  |  |  |  |  |  |
|  |  |  |  |  |  |  |  |  |  |  |
|  |  |  |  |  |  |  |  |  |  |  |
|  |  |  | respir | influenza INT- D 2 | 1 |  | 0 |  | NO | NO |
| *Ribes nigrum* L.  **(FP-SO 8)**  (CK, SO 1702) | mertskhali, (მერცხალი), smorodina (смородина) | 1 fruits, fresh | endoc | diabetes INT- E 1 | 1 | 0,02 | 0 | 0,00 | NO | NO |
|  |  |  |  |  |  |  |  |  |  |  |
|  |  |  |  |  |  |  |  |  |  |  |
|  |  |  |  |  |  |  |  |  |  |  |
|  |  |  |  |  |  |  |  |  |  |  |
|  |  |  |  |  |  |  |  |  |  |  |
|  |  |  |  |  |  |  |  |  |  |  |
|  |  |  |  |  |  |  |  |  |  |  |
|  |  |  |  |  |  |  |  |  |  |  |
| *Ribes* *petraeum* Wulfen  syn: Ribes  biebersteinii Berland. ex DC.  **(CK, SO 288*, 460)**  (CK, SO 475*, 622*, 1547) | motskhali, (motskhari, მოცხარი), smarodina, smorodina (смородина,smorodina,meskhali; *yerüzümü, merskali* | 1 leaves, infusion in water  2 fruits, vinegar  3 young leaves, infusion in water  4 fruits, sweet dish (compote)  5 fruits, infusion in water  6 fruits, fresh | GHUS | fever INT- D 1, 2, 3 | 5 | 0,20 | 0 | 0,03 | NO | x |
|  |  |  |  |  |  |  |  |  |  |  |
|  |  |  |  |  |  |  |  |  |  |  |
|  |  |  |  |  |  |  |  |  |  |  |
|  |  |  |  |  |  |  |  |  |  |  |
|  |  |  |  |  |  |  |  |  |  |  |
|  |  |  |  |  |  |  |  |  |  |  |
|  |  |  |  |  |  |  |  |  |  |  |
|  |  |  |  |  |  |  |  |  |  |  |
|  |  |  |  | good for health INT- E 4 |  |  |  |  | NO |  |
|  |  |  | respir | cough INT- D 1, 5 | 2 |  | 0 |  | NO | NO |
|  |  |  | endoc | diabetes *INT- E 6* | 0 |  | 2 |  | NO | NO |
|  |  |  | digest | toothache EXT- G 1 | 1 |  | 0 |  | NO | NO |
|  |  |  |  |  |  |  |  |  |  |  |
|  |  |  | skin | wound EXT- P 1 | 1 |  | 0 |  | NO | NO |
| *Ribes rubrum* L.  **(FP-SO 9)**  (CK, SO 58, 1703) | smarodina,  (смородина*)* | 1 fruits, sweet dish | GHUS | fever INT- E 1 | 1 | 0,02 | 0 | 0,00 | NO | NO |
|  |  |  |  |  |  |  |  |  |  |  |
|  |  |  |  |  |  |  |  |  |  |  |
|  |  |  |  |  |  |  |  |  |  |  |
|  |  |  |  |  |  |  |  |  |  |  |
|  |  |  |  |  |  |  |  |  |  |  |
|  |  |  |  |  |  |  |  |  |  |  |
|  |  |  |  |  |  |  |  |  |  |  |
|  |  |  |  |  |  |  |  |  |  |  |
| *Ribes* *uva-crispa* L.  syn: Grossularia reclinata (L.) Mill.  **(FP-SO 10)  (CK, SO 28*)** | (Крыжо́вник ,kryzhóvnik), krujavnik, smorodina,  haharch, (Հաղարջենի,հաղարջ, hagharjeni, hagharj) | 1 fruits, sweet dish (jam), 1:1 ratio of fruits and sugar, one table spoon  2 leaves, infusion in water  3 fruits, sweet dish (muraba)  4 fruits, drink (juice) | digest | diarrhoea INT- E 3 | 1 | 0,13 | 0 | 0,00 | NO | NO |
|  |  |  |  |  |  |  |  |  |  |  |
|  |  |  |  |  |  |  |  |  |  |  |
|  |  |  |  |  |  |  |  |  |  |  |
|  |  |  |  |  |  |  |  |  |  |  |
|  |  |  |  |  |  |  |  |  |  |  |
|  |  |  | GHUS | fever INT- E 3 | 1 |  | 0 |  | NO | NO |
|  |  |  | respir | cough INT- D 4, 2 | 4 |  | 0 |  | NO | NO |
|  |  |  |  | bronchitis INT- E 1 |  |  |  |  | NO |  |
|  |  |  |  | cold INT- E 1 |  |  |  |  | NO |  |
| HYPERICACEAE |  |  |  |  |  |  |  |  |  |  |
| *Hypericum bithynicum*Boiss.  *syn:* *Hypericum montbretii* var. *caucasicum* Woronov  **(CK, SO 41)**   CK, SO 80) | krazana (კრაზანა), kati balagha | 1 aerial parts with flowers, infusion in water | digest | stomach ache INT- D 1 | 4 | 0,13 | 0 | 0,00 | NO | NO |
|  |  |  |  | jaundice INT- D 1 |  |  |  |  | NO |  |
|  |  |  | genito | kidney pain INT- D 1 | 1 |  | 0 |  | NO | NO |
|  |  |  | GHUS | allergy INT- D 1 | 1 |  | 0 |  | NO | NO |
| *Hypericum linarioides*Bosse   (CK, SO 102, 314) | zveroboy (зверобой)*; kaymak çiçeği* | 1 aerial parts with flowers, infusion in water   2 aerial parts with flowers, decoction in water | GHUS | pain killer INT- D 1 | 1 | 0,02 | 0 | 0,08 | NO | NO |
|  |  |  | digest | stomach ache *INT- D 1; EXT- B 2* | 0 |  | 3 |  | NO | NO |
|  |  |  |  | diarrhea *INT- D 1* |  |  |  |  | NO |  |
|  |  |  | cardio | hemorrhoids *EXT- B 2* | 0 |  | 1 |  | NO | NO |
|  |  |  | blood | anemia *INT- D 1* | 0 |  | 1 |  | NO | NO |
|  |  |  | genito | kidney disease *INT- D 1* | 0 |  | 1 |  | NO | NO |
| *Hypericum montbretii* Spach  **(CK, SO 108)** | krazana (კრაზანა)*; mayasıl otu, kalba otu, mayasir otu, sarı çiçek* | 1 aerial parts with flowers, infusion in water   2 aerial parts with flowers, decoction in water | skin | eczema EXT- P 1 | 2 | 0,07 | 2 | 0,08 | NO | NO |
|  |  |  |  | wound EXT- P 1 |  |  |  |  | NO |  |
|  |  |  |  | antifungal *EXT- B 2* |  |  |  |  | NO |  |
|  |  |  |  | itching *INT- D 1* |  |  |  |  | NO |  |
|  |  |  | cardio | high blood pressure INT- D 1 | 1 |  | 2 |  | NO | x |
|  |  |  |  | hemorrhoids *EXT- B 1, 2* |  |  |  |  | (16) |  |
|  |  |  | genito | urinary disease *EXT- B 1* | 0 |  | 1 |  | NO | x |
|  |  |  | GHUS | fever *INT- D 1* | 0 |  | 1 |  | NO | NO |
| *Hypericum orientale*L.  s*yn: Hypericum ptarmicifolium* Spach  (CK, SO 7, 12) | *masır otu* | 1 aerial parts with flowers, infusion in water  2 aerial parts with flowers, decoction in water  3 entire plant, decoction in water | cardio | hemorrhoids *EXT- B 2, 3* | 0 | 0,00 | 2 | 0,04 | NO | NO |
|  |  |  | endoc | diabetes *INT- D 1* | 0 |  | 1 |  | NO | NO |
| *Hypericum perforatum* L.  (**CK, SO 475)** (CK, SO 1480) | krazana (კრაზანა), zveroboy (зверобой)*; lokman hekim çayı,  kaymak çiçeği, yayla çiçeği, sarı kantaron, mayasıl otu, basur çayı* | 1 aerial parts with flowers, infusion in water  2 aerial parts with flowers, decoction in water  3 aerial parts with flowers, infusion in oil   4 aerial parts with flowers, dried and powdered   5 aerial parts with flowers, infusion in water, mix with *Thymus sp. , Anthemis sp.* | cardio | high blood pressure INT- D 1 | 2 | 0,47 | 3 | 0,14 | (10) | x |
|  |  |  |  | hemorrhoids *EXT- B 1, 2; INT- D 1* |  |  |  |  | (10), (20), (15) |  |
|  |  |  | digest | diarrhea INT- D 1 | 9 |  | 3 |  | (15) | x |
|  |  |  |  | stomach ache **INT- D 1** |  |  |  |  | (6), (2), (16), (14), (9) |  |
|  |  |  |  | liver disease INT- D 1 |  |  |  |  | (3), (24), (15) |  |
|  |  |  |  | prevent vomiting INT- D 1 |  |  |  |  | NO |  |
|  |  |  |  | constipation INT- D 1 |  |  |  |  | NO |  |
|  |  |  |  | gall disease INT- D 1 |  |  |  |  | (24) |  |
|  |  |  | endoc | loss of appetite INT- D 1 | 1 |  | 0 |  | NO | NO |
|  |  |  | genito | kidney disease **INT- D 1** | 3 |  | 1 |  | (3), (24), (16) | x |
|  |  |  |  | urinary disease INT- D 1 |  |  |  |  | NO |  |
|  |  |  | GHUS | good for health INT- D 5 | 1 |  | 0 |  | (25) | x |
|  |  |  | psych | relaxing INT- D 1 | 1 |  | 0 |  | (11) | x |
|  |  |  | respir | influenza INT- D 1 | 2 |  | 1 |  | NO | NO |
|  |  |  |  | throat ache INT- D 1 |  |  |  |  | NO |  |
|  |  |  |  | cough *INT- D 1* |  |  |  |  | NO |  |
|  |  |  | skin | skin care complaints EXT- B 5 | 2 |  | 2 |  | NO | x |
|  |  |  |  | antiseptic INT- D 1 |  |  |  |  | (3) |  |
|  |  |  |  | wound *EXT- P 3, 4* |  |  |  |  | (10), (11), (20), (21), (23) |  |
| IRIDACEAE |  |  |  |  |  |  |  |  |  |  |
| *Crocus vallicola Herb.* **(CK, SO 265)** | satovliya | 1 bulb, fresh | GHUS | cancer INT- E 1 | 1 | 0,02 | 0 | 0,00 | NO | NO |
| JUGLANDACEAE |  |  |  |  |  |  |  |  |  |  |
| *Juglans regia* L.  (CK, SO 516*) | kakali (კაკალი), nigozi (ნიგოზი), ceviz; *ceviz,zengo, gagal,* | 1 pericarp (husk), infusion in water  2 leaves, infusion in water  3 aerial roots, children pass through the aerial root of walnut  4 fruits, fresh  5 leaves, fresh  6 aerial roots, washing wart with water on aerial root walnut tree | digest | tooth bleaching *EXT- G 1* | 0 | 0,02 | 3 | 0,09 | NO | x |
|  |  |  |  |  |  |  |  |  |  |  |
|  |  |  |  |  |  |  |  |  |  |  |
|  |  |  |  |  |  |  |  |  |  |  |
|  |  |  |  |  |  |  |  |  |  |  |
|  |  |  |  |  |  |  |  |  |  |  |
|  |  |  |  |  |  |  |  |  |  |  |
|  |  |  |  |  |  |  |  |  |  |  |
|  |  |  |  |  |  |  |  |  |  |  |
|  |  |  |  |  |  |  |  |  |  |  |
|  |  |  | neuro | headache *EXT- H 5* | 0 |  | 1 |  | NO | NO |
|  |  |  | skin | antifungal *EXT- P 2* | 0 |  | 2 |  | (18) | x |
|  |  |  |  | wart *RT-6* |  |  |  |  | NO |  |
|  |  |  | GHUS | measles *EXT- RT 3* | 0 |  | 1 |  | NO | x |
|  |  |  | endoc | thyroid INT- E 4 | 1 |  | 0 |  | (2), (12), (19), (26) | x |
| LAMIACEAE |  |  |  |  |  |  |  |  |  |  |
| *Clinopodium grandiflorum* (L.) Kuntze  *syn: Calamintha grandiflora* (L.) Moench  **(CK, SO 76)** | kaplibalakha (კაპლიბალახა), kaplisuna | 1 aerial parts with flowers,  infusion in water | psych | relaxing INT- D 1 | 3 | 0,13 | 0 | 0,00 | NO | NO |
|  |  |  |  | sedative INT- D 1 |  |  |  |  | NO |  |
|  |  |  | cardio | heart disease INT- D 1 | 1 |  | 0 |  | NO | NO |
|  |  |  | GHUS | good for health INT- D 1 | 1 |  | 0 |  | (15) | x |
|  |  |  | endoc | increase milk supply INT- D 1 | 1 |  | 0 |  | NO | NO |
| *Leonurus* sp.   **(CK, SO 20)** | guluhaya krapiva | 1 leaves, dried and powdered, infusion in votka keep one week in dark place. | neuro | nervous disease INT- D 1 | 1 | 0,02 | 0 | 0,00 | NO | x |
| *Mentha longifolia* (L.) L.  **(CK, SO 468)**  (CK, SO 683) | tentso,  veluri pitna, pitna (პიტნა), daghdz (դաղձ), nana, gareuli pitna; *yarpuz,  tsetso, yabani nane, pitna, nane, punk* | 1 aerial parts with flowers,  infusion in water  2 aerial parts with flowers, fresh  3 aerial parts with flowers, infusion in water, mix with chammomile  4 aerial parts with flowers, deocoction in water  5 aerial parts with flowers, infusion in water, mix with *Viburnum* sp.  6 aerial parts with flowers, infusion in water mix with *nego, bamba balakhi, askili, gvirila* | cardio | high blood pressure INT- D 1 | 6 | 0,56 | 0 | 0,19 | NO | x |
|  |  |  |  | heart disease INT- D 5 |  |  |  |  | (3), (26) |  |
|  |  |  | digest | stomach ache **INT**- E 2, *D 1* | 3 |  | 1 |  | (13), (6), (12), (20), (17), (9) | x |
|  |  |  | eye | eye diseases INT- D 1 | 1 |  | 0 |  | NO | NO |
|  |  |  | GHUS | allergy EXT- B 3 | 2 |  | 0 |  | NO | x |
|  |  |  | psych | relaxing INT- D 1 | 6 |  | 0 |  | (3), (26) | x |
|  |  |  |  | sleep disorders INT- D 1 |  |  |  |  | (26) |  |
|  |  |  | respir | lung disease INT- D 1 | 4 |  | 2 |  | (16) | x |
|  |  |  |  | influenza INT- D 1 |  |  |  |  | (14) |  |
|  |  |  |  | cold **INT- D** 1, *4* |  |  |  |  | (5), (16), (14) |  |
|  |  |  |  | bronchitis INT- D 6 |  |  |  |  | (13) |  |
|  |  |  | skin | hair care complaints (hair wash) **EXT- B 1** | 2 |  | 4 |  | NO | x |
|  |  |  |  | antiseptic EXT- B 4 |  |  |  |  | (26) |  |
|  |  |  |  | wound *EXT- P 2* |  |  |  |  | (26) |  |
|  |  |  |  | skin care complaints *EXT- B 1* |  |  |  |  | NO |  |
|  |  |  | endoc | increase milk supply *INT- D 1* | 0 |  | 5 |  | NO | x |
|  |  |  | mu-sk | rheumatism *INT- D 1* | 0 |  | 1 |  | NO | x |
|  |  |  | neuro | headache *INT- D 4* | 0 |  | 1 |  | (13), (26) | x |
|  |  |  | genito | woman disease INT- D 1 | 1 |  | 0 |  | NO | x |
| *Nepeta betonicifolia* C.A.Mey.  s*yn: Nepeta grossheimii* Pojark.  **(FP-SO 68)** | daghdz (դաղձ) | 1 aerial parts, infusion in water | cardio | high blood pressure INT- D 1 | 1 | 0,02 | 0 | 0,00 | NO | NO |
| *Origanum vulgare* L.  **(CK,SO 478)**  /CK,SO 1283) | tavshava (თავშავა), dushitza, marmuchi, urtz, ombalo; *çay otu, çam çayı, kekik* | 1 aerial parts, infusion in water  2 aerial parts with flowers, deocoction in water | cardio | high blood pressure INT- D 1 | 1 | 0,29 | 2 | 0,16 | (16) | x |
|  |  |  |  | hemorrhoids *INT- D 1; EXT- B 2* |  |  |  |  | NO |  |
|  |  |  | digest | stomach ache INT- D 1 | 5 |  | 4 |  | (14) | x |
|  |  |  |  | intestinal disease INT- D 1 |  |  |  |  | NO |  |
|  |  |  |  | diarrhea INT- D 1 |  |  |  |  | NO |  |
|  |  |  |  | gastrointestinal infection *INT- D 1* |  |  |  |  | (16) |  |
|  |  |  |  | crying babies *INT- D 1* |  |  |  |  | NO |  |
|  |  |  | genito | woman disease **INT- D 1** | 1 |  | 1 |  | NO | x |
|  |  |  | GHUS | good for health INT- D 1 | 3 |  | 0 |  | NO | NO |
|  |  |  |  | fever INT- D 1 |  |  |  |  | NO |  |
|  |  |  | mu-sk | back pain INT- D 1 | 1 |  | 1 |  | NO | NO |
|  |  |  |  | rheumatism *INT- D 1* |  |  |  |  | NO |  |
|  |  |  | respir | cough **INT- D 1** | 2 |  | 2 |  | NO | x |
|  |  |  |  | throat ache *INT- D 1* |  |  |  |  | NO |  |
|  |  |  | skin | hair care complaints (hair wash) *EXT- B 2* | 0 |  | 2 |  | NO | x |
|  |  |  |  | itching *EXT- B 2* |  |  |  |  | NO |  |
| *Prunella vulgaris* L.  **(CK, SO 27, 67, 517)** | putkris balakhi,  nagzobi balakhi | 1 aerial parts with flowers, infusion in water  2 aerial parts with flowers,  decoction in water with antzli leaves | skin | wound EXT- P 1 | 2 | 0,04 | 0 | 0,00 | NO | x |
|  |  |  |  | bruises EXT- P 2 |  |  |  |  | NO |  |
| *Salvia glutinosa* L.   (FP-SO 69) | *purçimela* | 1 aerial parts with flowers, fresh  2 leaves, dried and powdered | skin | wound *EXT- P 1* | 0 | 0,00 | 3 | 0,04 | (10) | x |
|  |  |  |  | wart *EXT- P 1* |  |  |  |  | NO |  |
|  |  |  |  | bleedind wound *EXT- P 2* |  |  |  |  | NO |  |
| *Satureja spicigera* (K.Koch) Boiss.  **(FP-SO 11)**  (CK, SO 519*, 581*, 619*, 631*, 1479, 1793) | kondari (ქონდარი), veluri kondari; *kondar* | 1 leaves, fresh  2 young aerial parts with flowers, mixed with yoghurt  3 young aerial parts with flowers, infusion in water  4 young aerial parts with flowers, decoction in water, mixed with simindis puncuri (corn silk) and maghadanos (parsley) root, drink three times a day with coffee cup | digest | stomach ache INT- E 1, D 3 | 4 | 0,33 | 0 | 0,01 | (26) | x |
|  |  |  |  |  |  |  |  |  |  |  |
|  |  |  |  |  |  |  |  |  |  |  |
|  |  |  |  |  |  |  |  |  | NO |  |
|  |  |  | GHUS | general disease INT- D 4 | 4 |  | 0 |  | NO | NO |
|  |  |  | skin | blister EXT- P 2 | 2 |  | 0 |  | NO | NO |
|  |  |  | endoc | diabetes INT- E 2, D 3 | 4 |  | 0 |  | NO | NO |
|  |  |  | respir | cough INT- D 3 | 1 |  | 0 |  | (26) | x |
|  |  |  | genito | women disease (infertility) *INT- D 3* | 0 |  | 1 |  | NO | NO |
| *Teucrium polium* L.  **(CK, SO 63)**  (CK, SO 23) | *bağsur otu, aylık otu* | 1 aerial parts, infusion in water  2 aerial parts with flowers, deocoction in water | cardio | hemorrhoids *INT- D 1* | 0 | 0,00 | 1 | 0,07 | (13), (16), (18) | x |
|  |  |  | eye | hypopyon *EXT- B 2* | 0 |  | 2 |  | NO | NO |
|  |  |  | digest | diarrhea *INT- D 1* | 0 |  | 1 |  | (13) | x |
|  |  |  |  | stomach ache *INT- D 1* |  |  |  |  | (16), (14) |  |
| *Thymus* spp*. (Thymus praecox* subsp. *grossheimii* (Ronniger) Jalas*; Thymus praecox* subsp*. caucasicus* (Willd. ex Ronniger) Jalas)  *syn: Thymus grossheimii* Ronniger; *Thymus caucasicus* Willd. ex Ronniger;   **(CK, SO 466)**  (CK, SO 1458, 1768, 1793) | chebrets, has zetron, daghdz (դաղձ), kek otu, zetron*; adaçayı,  kekik,  dağ çayı,  sancı otu, mayasir otu, mor çiçek, sancı otu, yayla çayı, kek otu, çay otu* | 1 aerial parts with flowers, infusion in water  2 aerial parts with flower, mix with chammomile and infusion in water  3 aerial parts with flower, infusion in water mix with Hypericum sp. , Anthemis sp.  4 aerial parts with flower, decoction in water  5 leaves, infusion in water  6 leaves, decoction in water | cardio | high blood pressure INT- D 4, 5 | 7 | 0,29 | 1 | 0,11 | (16) | x |
|  |  |  |  | hemorrhoids *EXT- B 4* |  |  |  |  | NO |  |
|  |  |  |  | heart disease INT- D 1 |  |  |  |  | NO |  |
|  |  |  | digest | diarrhea INT- D 5 | 1 |  | 1 |  | NO | x |
|  |  |  |  | stomach ache *INT- D 1* |  |  |  |  | (5), (2) |  |
|  |  |  | respir | bronchitis INT- D 5 | 2 |  | 2 |  | NO | x |
|  |  |  |  | cough INT- D 5, *6* |  |  |  |  | NO |  |
|  |  |  |  | cold *INT- D 5* |  |  |  |  | (2), (16) |  |
|  |  |  | GHUS | good for health **INT- D** *1*, 3 | 1 |  | 2 |  | NO | NO |
|  |  |  |  | sunstroke *INT- D 1* |  |  |  |  | NO |  |
|  |  |  | neuro | headache INT- D 1 | 1 |  | 0 |  | NO | NO |
|  |  |  | skin | skin care complaints EXT- B 3 | 1 |  | 1 |  | NO | x |
|  |  |  |  | boil *EXT- B 5* |  |  |  |  | NO |  |
|  |  |  | genito | vaginal discharge *INT- D 2* | 0 |  | 1 |  | NO | x |
| *Ziziphora clinopodioides*Lam. *syn: Ziziphora serpyllacea* M.Bieb.   (CK, SO 1585,1637) | *mayasıl otu* | 1 aerial parts with flowers, infusion in water | cardio | hemorrhoids *INT- D 1, EXT- B 1* | 0 | 0,00 | 2 | 0,04 | NO | NO |
|  |  |  | mu-sk | footsoreness *EXT- B 1* | 0 |  | 1 |  | NO | NO |
| LAURACEAE |  |  |  |  |  |  |  |  |  |  |
| *Laurus nobilis* L. | (dapna,დაფნა), dapne | 1 leaves, infusion in water | digest | toothache EXT- G 1 | 1 | 0,02 | 0 | 0,00 | (10) | x |
|  |  |  |  |  |  |  |  |  |  |  |
|  |  |  |  |  |  |  |  |  |  |  |
|  |  |  |  |  |  |  |  |  |  |  |
| MALVACEAE |  |  |  |  |  |  |  |  |  |  |
| *Alcea calvertii* (Boiss.) Boiss.  **(CK, SO 65)**  (CK, SO 1573) | *gül hatmi, hanımkız, gülhatın* | 1 flowers, decoction in water  2 flowers, infusion in water  3 aerial parts with flowers, decoction in water | mu-sk | rheumatism  *INT- D 1* | 0 | 0,00 | 1 | 0,07 | NO | NO |
|  |  |  | genito | women disease (infertility) *INT- D 1* | 0 |  | 4 |  | NO | x |
|  |  |  |  | menstruation problems *INT- D 2* |  |  |  |  | NO |  |
|  |  |  |  | women disease (inflammation) *EXT- B 3* |  |  |  |  | NO |  |
| *Malva neglecta* Wallr.  **(CK, SO 467, 493)**  (CK, SO 1686, 1745) | moloka (მოლოქა); *molokva,  gorcolo otu,  korgodan,  molok,  moloki, ebegümeci, korkut, goga,  doluk,  dolık* | 1 aerial parts with flowers, decoction in water  2 aerial parts, decoction in water with stingnettle  3 aerial parts with flowers, infusion in water  4 leaves, decoction in milk mixed with barley flour  5 leaves, decoction in water mixed with *pancar, kevkesk, pispis, belhevis* leaves  6 seeds, decoction in water  7 seeds, infusion in water  8 fruits, fresh  9 flowers, macerated  10 roots, decoction in water  11 roots, fresh  12 leaves, decoction in water with corn flour | cardio | hemorrhoids *EXT- B 4* | 0 | 0,02 | 1 | 0,18 | (17), (26) | x |
|  |  |  | digest | worm *INT- D 2* | 0 |  | 1 |  | NO | x |
|  |  |  | endoc | diabetes *INT- D 3* | 0 |  | 1 |  | NO | NO |
|  |  |  | genito | vaginal discharge *EXT- B 6* | 0 |  | 2 |  | NO | x |
|  |  |  |  | abortion *INT- D 10* |  |  |  |  | NO |  |
|  |  |  | mu-sk | rheumatism *EXT- B 5* | 0 |  | 1 |  | (2), (6) | x |
|  |  |  | respir | expectoration *INT- D 7* | 0 |  | 3 |  | NO | x |
|  |  |  |  | sinusitis *INT- V 1* |  |  |  |  | NO |  |
|  |  |  |  | quinsy *INT- E 8* |  |  |  |  | (22) |  |
|  |  |  | skin | itching *EXT- B 1* | 1 |  | 4 |  | NO | x |
|  |  |  |  | boil **EXT- P** *4, 11,* 12*, EM 9* |  |  |  |  | (13), (14), (26), (8) |  |
| *Tilia rubra* subsp.*caucasica* (Rupr.) V.Engl. *syn: Tilia  caucasica*Rupr.  **(FP-SO 12)**  (CK, SO 1765) | tsatskhvi,  (ცაცხვი); *ıhlamur* | 1 leaves with flowers, infusion in water  2 leaves with flowers, decoction in water | respir | influenza **INT- D** *1,* 2 | 2 | 0,09 | 3 | 0,04 | (2), (11), (19), (21), (22) | x |
|  |  |  |  |  |  |  |  |  |  |  |
|  |  |  |  |  |  |  |  |  |  |  |
|  |  |  |  |  |  |  |  |  |  |  |
|  |  |  |  |  |  |  |  |  |  |  |
|  |  |  |  |  |  |  |  |  |  |  |
|  |  |  |  |  |  |  |  |  |  |  |
|  |  |  |  |  |  |  |  |  |  |  |
|  |  |  |  | throat ache **INT- D** *1,* 2 |  |  |  |  | (22) |  |
|  |  |  | psych | relaxing INT- D 1, 2 | 2 |  | 0 |  | NO | NO |
| MELANTHIACEAE |  |  |  |  |  |  |  |  |  |  |
| *Veratrum album* L.  **(CK, SO 52)**  (CK, SO 1546) | *abitarat* | 1 leaves, decoction in water  2 roots, macerated | mu-sk | bone and joint pain *EXT- P 1* | 0 | 0,02 | 1 | 0,01 | NO | NO |
|  |  |  | skin | wound EXT- P 2 | 1 |  | 0 |  | NO | NO |
| PAPAVERACEAE |  |  |  |  |  |  |  |  |  |  |
| *Chelidonium majus* L.  **(FP-SO 70)** | chistotel (чистотел) kristesis, kristesiskhla (ქრისტესისხლა) | 1 sap, fresh  2 entire plant, fresh  3 aerial parts, decoction in water  4 aerial parts, infusion in water | skin | wart **EXT- P 1,** EM- 1 | 7 | 0,29 | 2 | 0,03 | (1), (24), (25), (10), (26), (21), (15) | x |
|  |  |  |  | wound EXT- P 2 |  |  |  |  | (24) |  |
|  |  |  | cardio | heart disease INT- D 3 | 1 |  | 0 |  | NO | NO |
|  |  |  | genito | kidney disease INT- D 3 | 1 |  | 0 |  | NO | x |
|  |  |  | digest | stomach ache INT- D 3 | 1 |  | 0 |  | NO | x |
|  |  |  | respir | throat ache EXT- G 3, 4 | 2 |  | 0 |  | NO | NO |
|  |  |  | mu-sk | bone and joint pain EXT- P 3 | 1 |  | 0 |  | NO | NO |
| *Fumaria officinalis* L.  (CK-SO 316) | *mayasır otu* | 1 aerial parts with flowers, decoction in water  2 aerial parts with flowers, infusion in water | skin | itching *INT- D 2* | 0 | 0,00 | 1 | 0,04 | (8) | x |
|  |  |  | cardio | hemorrhoids *INT- D 2; EXT- B 1* | 0 |  | 2 |  | (6) | x |
| *Papaver orientale* L.  **(CK, SO 543)**  (CK, SO 1561) | kakacho (ყაყაჩო) bangi; *haşhaş,  dodopol, diş otu, kakaço* | 1 flowers, dried, infusion in water  2 seeds, fresh  3 seed, infusion in water  4 flowers, infusion in milk  5 seeds, burned and put in water  6 leaves, fresh | psych | relaxing INT- D 1 | 3 | 0,07 | 1 | 0,07 | (9) | x |
|  |  |  |  | sedative **INT**- E 2, *D 4* |  |  |  |  | NO |  |
|  |  |  |  | sleep disorders INT- D 3 |  |  |  |  | NO |  |
|  |  |  | digest | constipation *INT- E 2* | 0 |  | 3 |  | NO | x |
|  |  |  |  | toothache *INT- V 5* |  |  |  |  | NO |  |
|  |  |  | neuro | headache *EXT- H 6* | 0 |  | 1 |  | NO | NO |
| PINACEAE |  |  |  |  |  |  |  |  |  |  |
| *Abies nordmanniana* (Steven) Spach  **(FP-SO 1**)  (CK, SO 364*, 405*, 424*, 466*, 606*) | sochi (სოჭი) | 1 entire plant, breath in the forest of this tree  2 resin, infusion in water  3 young seed cones, infusion in water  4 resin, macerated | respir | bronchitis INT- RT 1 | 3 | 0,11 | 0 | 0,00 | NO | NO |
|  |  |  |  |  |  |  |  |  |  |  |
|  |  |  |  |  |  |  |  |  |  |  |
|  |  |  |  |  |  |  |  |  |  |  |
|  |  |  |  |  |  |  |  |  |  |  |
|  |  |  |  |  |  |  |  |  |  |  |
|  |  |  |  |  |  |  |  |  |  |  |
|  |  |  |  | cough INT- D 2 |  |  |  |  | NO |  |
|  |  |  |  | lung disease INT- D 3 |  |  |  |  | NO |  |
|  |  |  | GHUS | tuberculosis INT- RT 1 | 1 |  | 0 |  | (24) | x |
|  |  |  | skin | wound EXT- P 4 | 1 |  | 0 |  | (13) | x |
| *Picea orientalis* (L.) Peterm.  **(CK, SO 26*, 110, 446)** (CK, SO 404*, 462*, 658, 1041, 1676) | nadzvi (ნაძვი), yel; *ladin,köknar, küknar, göknar, nadzvi* | 1 resin (pisi, khevi, çsisip), fresh  2 resin (khevi), infusion in water and mixed with honey  3 entire plant, breath in the forest of this tree  4 resin, decoction in water  5 resin, macerated  6 young seed cones, infusion in water  7 resin, infusion in water  8 pollen cones, infusion in water  9 resin, decoction in milk  10 resin, macerated and mixed with melted beewax, butter and olive oil  11 young seed and pollen cones, infusion in water | digest | stomach ache **INT- C 1,** *E 1* | 8 | 0,60 | 21 | 0,59 | (22) | x |
|  |  |  |  |  |  |  |  |  |  |  |
|  |  |  |  |  |  |  |  |  |  |  |
|  |  |  |  |  |  |  |  |  |  |  |
|  |  |  |  |  |  |  |  |  | NO |  |
|  |  |  |  | ulcer *INT-* *C 1, E 1* |  |  |  |  | (19), (20), (22), (24) |  |
|  |  |  |  | toothache INT- C 1 |  |  |  |  | NO |  |
|  |  |  |  | tooth bleaching **EXT- G 4** |  |  |  |  | NO |  |
|  |  |  | GHUS | tuberculosis INT- RT 3 | 1 |  | 0 |  | (19), (24) | x |
|  |  |  | genito | vaginal discharge *INT- E 1* | 0 |  | 1 |  | NO | NO |
|  |  |  |  |  |  |  |  |  |  |  |
|  |  |  | mu-sk | fracture *EXT- P 5* | 1 |  | 3 |  | NO | NO |
|  |  |  |  | sprain EXT- P 5 |  |  |  |  | NO |  |
|  |  |  | respir | tonsil INT- D 6 | 9 |  | 1 |  | NO | x |
|  |  |  |  | asthma INT- RT 3 |  |  |  |  | NO |  |
|  |  |  |  | lung disease **INT- D** 2, 6, 9, 11, *7*, C 1 |  |  |  |  | (19) |  |
|  |  |  |  |  |  |  |  |  |  |  |
|  |  |  |  | bronchitis INT- D 8 |  |  |  |  | (1) |  |
|  |  |  |  | cough INT- D 7 |  |  |  |  | NO |  |
|  |  |  | skin | wound **EXT- P 5**, *S 10* | 8 |  | 17 |  | (1), (10), (11), (20), (22) | x |
|  |  |  |  | bruises *EXT- P 5* |  |  |  |  | NO |  |
|  |  |  |  | burn *EXT- P 5* |  |  |  |  | (11), (19) |  |
|  |  |  |  | antiseptic *EXT- P 5* |  |  |  |  | NO |  |
|  |  |  |  | boil **EXT- P 5** |  |  |  |  | NO |  |
|  |  |  |  | antibacterial EXT- P 5 |  |  |  |  | NO |  |
| *Pinus sylvestris* var. *hamata* Steven  syn*: Pinus  sosnowskyi* Nakai  **(CK, SO 77, 447)**  (CK, SO 543*, 1175) | pich’vi (ფიჭვი), katris pichvi, sosna (сосна), katari (კატარი, k’at’ari), katri, tonazar, gadik*; çam, çam sakızı, toruk, ifti, küknar* | 1 pollen cones, infusion in water  2 young seed cones, infusion in water  3 heartwood, put near pillow  4 resin, fresh  5 young seed cones, decoction in water  6 pollen cones, mixed with honey  7 young seed cones, sweet dish (jam)  8 young seed and pollen cones, sweet dish (compote, jam)  9 young seed and pollen cones, decoction in water  10 young seed and pollen cones, infusion in water  11 young seed and pollen cones, mixed with honey  12 pollen cones, macerated  13 resin (pisi), macerated | cardio | high blood pressure INT- D 1, 2; EXT- RT 3 | 3 | 0,67 | 0 | 0,12 | NO | NO |
|  |  |  |  |  |  |  |  |  |  |  |
|  |  |  |  |  |  |  |  |  |  |  |
|  |  |  |  |  |  |  |  |  |  |  |
|  |  |  |  |  |  |  |  |  |  |  |
|  |  |  |  |  |  |  |  |  |  |  |
|  |  |  |  |  |  |  |  |  |  |  |
|  |  |  |  |  |  |  |  |  |  |  |
|  |  |  |  |  |  |  |  |  |  |  |
|  |  |  |  |  |  |  |  |  |  |  |
|  |  |  |  |  |  |  |  |  |  |  |
|  |  |  |  |  |  |  |  |  |  |  |
|  |  |  |  |  |  |  |  |  |  |  |
|  |  |  |  |  |  |  |  |  |  |  |
|  |  |  |  |  |  |  |  |  |  |  |
|  |  |  |  |  |  |  |  |  |  |  |
|  |  |  |  |  |  |  |  |  |  |  |
|  |  |  |  |  |  |  |  |  |  |  |
|  |  |  |  |  |  |  |  |  |  |  |
|  |  |  |  |  |  |  |  |  |  |  |
|  |  |  |  |  |  |  |  |  |  |  |
|  |  |  |  |  |  |  |  |  |  |  |
|  |  |  |  |  |  |  |  |  |  |  |
|  |  |  |  |  |  |  |  |  |  |  |
|  |  |  | digest | stomach ache **INT- C 4**, *E 4* | 3 |  | 3 |  | (12) | x |
|  |  |  |  | toothache EXT- G 5 |  |  |  |  | (6) |  |
|  |  |  | endoc | diabetes *INT- D 2* | 0 |  | 2 |  | NO | NO |
|  |  |  | GHUS | tuberculosis INT- E 6 | 2 |  | 0 |  | (24), (25) | x |
|  |  |  | genito | diuretic *INT- D 5* | 1 |  | 1 |  | (25), (26) | x |
|  |  |  |  | urinary disease INT- E 7 |  |  |  |  | (1) |  |
|  |  |  | mu-sk | rheumatism EXT- B 5 | 1 |  | 0 |  | (12), (26) | x |
|  |  |  | respir | lung disease INT- D 1, 10, E 7,  8, 6, 11, V 9 | 18 |  | 2 |  | (19), (25) | x |
|  |  |  |  |  |  |  |  |  |  |  |
|  |  |  |  |  |  |  |  |  |  |  |
|  |  |  |  | cough INT- D 1, 2 |  |  |  |  | (1), (6), (26) |  |
|  |  |  |  | asthma **INT- D** 1, *2*, E 7 |  |  |  |  | (1) |  |
|  |  |  |  |  |  |  |  |  |  |  |
|  |  |  |  | bronchitis **INT- D** 1, 10, 2, E 6, 11, 8 |  |  |  |  | (1), (12), (19), (25) |  |
|  |  |  |  |  |  |  |  |  |  |  |
|  |  |  | skin | wound *EXT- P 12* | 2 |  | 1 |  | (6), (8), (11), (18), (19), (26) | x |
|  |  |  |  | antibacterial EXT- P 13 |  |  |  |  | (13) |  |
|  |  |  |  | burn EXT- P 13 |  |  |  |  | (11), (19), (25), (26) |  |
| PLANTAGINACEAE |  |  |  |  |  |  |  |  |  |  |
| *Plantago lanceolata* L.  **(FP-SO 71)** | ham ba yaprak,  (подорожник) podoroznik, yezan lezu (Եզան լեզու); *yara yaprağı, bağa yaprağı, belhevis* | 1 leaves, infusion in water  2 leaves, fresh, mix with honey  3 leaves, fresh  4 leaves, decoction in milk  5 leaves, macerated | skin | boil EXT- B 2 | 1 | 0,07 | 3 | 0,05 | (10), (8), (19), (22) | x |
|  |  |  |  | wound *EXT- P 3, 5* |  |  |  |  | (3), (8), (14), (20), (18), (19), (22), (15) |  |
|  |  |  |  | inflammation *EXT- B 4* |  |  |  |  | (16), (8), (21), (18), (15) |  |
|  |  |  | endoc | sexual development (induce oestrogen level) INT- C 3 | 1 |  | 0 |  | NO | NO |
|  |  |  | digest | stomach ache INT- D 1 | 1 |  | 0 |  | (21), (17), (18), (15) | x |
|  |  |  | respir | cough *INT- D 1* | 0 |  | 1 |  | NO | x |
| *Plantago major* L.  **(CK, SO 515, 535)**  (CK, SO 53, 1563) | mravadlzarghva (მრავალძარღვა), baghi yaprak, balargzargva, has bagha yaprak, podoroznik (подорожник), yezan lezu (Եզան լեզու); bağa yaprağı,  yedi damar, *ohte damar, çkvit damari, belhavıs, yara yaprağı, balarzarghva, şortuk, sinirli ot, singir otu, damarlı ot, garduk, bağa yaprağı, belhebis, belgahavas* | 1 entire plant, juice  2 leaves, infusion in water  3 roots, decoction in water  4 roots, infusion in water  5 seeds, decoction in water  6 leaves, fresh  7 leaves, juice  8 leaves, fresh, mix with honey and *Polygonum aviculare*  9 leaves, infusion in votka  10 leaves, mix with beewax  11 leaves, mix with honey  12 leaves, macerated  13 leaves, infusion in whey  14 leaves, warmed on stove  15 leaves, mix with pine resin  16 leaves, decoction in water  17 leaves, infusion in water mix with stingnettle, thyme, chammomile  18 leaves, decoction in milk  19 leaves, fresh, mix with olive and salt | digest | stomach ache **INT- D** 1, 2, 3, *4,* 7, E *6* | 19 | 1,44 | 7 | 1,20 | (4), (6), (25), (10), (20), (9) | x |
|  |  |  |  | diarrhea **INT- D** 2, 3, *5* |  |  |  |  | NO |  |
|  |  |  |  | toothache EXT- G 3 |  |  |  |  | (6) |  |
|  |  |  |  | gall disease INT- D 2 |  |  |  |  | NO |  |
|  |  |  |  | constipation INT- D 2 |  |  |  |  | NO |  |
|  |  |  |  | liver disease INT- C 6, D 2 |  |  |  |  | NO |  |
|  |  |  |  | ulcer *INT- D 2, 17* |  |  |  |  | (24), (16) |  |
|  |  |  |  | gastrointestinal infection *INT- D 2, E 6* |  |  |  |  | (24), (20), (15) |  |
|  |  |  | blood | cleansing blood INT- D 2 | 1 |  | 0 |  | (22) | x |
|  |  |  | cardio | hemorrhoids **INT**- E 6, *D 2* | 1 |  | 1 |  | (13), (16), (10), (12, (20) | x |
|  |  |  | genito | kidney disease **INT**- E 8, *D 2, 7* | 1 |  | 7 |  | (19), (9) | x |
|  |  |  |  |  |  |  |  |  | NO |  |
|  |  |  |  | vaginal discharge *INT- D 16* |  |  |  |  | NO |  |
|  |  |  |  | bladder infection *INT- D 2* |  |  |  |  | NO |  |
|  |  |  |  | menstruation problems *INT- D 2* |  |  |  |  | NO |  |
|  |  |  |  | woman disease *INT- D 2* |  |  |  |  | (8) |  |
|  |  |  | endoc | sexual development (induce oestrogen level) INT- C 6 | 1 |  | 0 |  | NO | x |
|  |  |  | GHUS | cleaning organs INT- D 2 | 2 |  | 1 |  | NO | x |
|  |  |  |  | good for health INT- D 9 |  |  |  |  | NO |  |
|  |  |  |  | pain killer *EXT- P 12* |  |  |  |  | NO |  |
|  |  |  | mu-sk | bone and joint pain EXT- P 10 | 1 |  | 3 |  | NO | x |
|  |  |  |  | rheumatism *INT- D 2* |  |  |  |  | (2), (12) |  |
|  |  |  |  | numbness in arm *INT- D 16* |  |  |  |  | NO |  |
|  |  |  |  | fracture *EXT- P 12* |  |  |  |  | NO |  |
|  |  |  | respir | throat ache INT- D 2 | 1 |  | 5 |  | (5) | x |
|  |  |  |  | expectoration *INT- D 16* |  |  |  |  | (11) |  |
|  |  |  |  | bronchitis *INT- D 2* |  |  |  |  | NO |  |
|  |  |  |  | cough *INT- D 2* |  |  |  |  | (24), (25), (15) |  |
|  |  |  |  | shortness of breath *INT- D 2* |  |  |  |  | (8) |  |
|  |  |  | skin | boil **EXT- P** *2***, 6**, 10, 11, **12**, 13, **14**, 15, B 11, 16, *INT- D 2* | 38 |  | 65 |  | (4), (13), (17), (2), (8), (14), (19), (9), (22) | x |
|  |  |  |  | wound **EXT- P 6, 12**, *14, D 18* |  |  |  |  | (3), (13), (6), (8), (5), (24), (18), (25), (12), (21), (14), (19), (9), (15) |  |
|  |  |  |  | bleeding wound EXT- P 12 |  |  |  |  | (6), (24) |  |
|  |  |  |  | inflammation **INT- D 2;** *EXT- P 6, 12, 18, B 18* |  |  |  |  | (2), (16), (10), (8), (20), (18), (9), (22), (15) |  |
|  |  |  |  | insect bites *EXT- P 6, 19* |  |  |  |  | NO |  |
| POLYGONACEAE |  |  |  |  |  |  |  |  |  |  |
| *Polygonum carneum* C. Koch  **(CK, SO 68*)**  (CK, SO 511) | dvaluri (დვალური)*; dıvaruli, pancar, yayla pancarı* | 1 leaves, infusion in water  2 leaves, decoction in water  3 leaves, fresh  4 flowers, infusion in water | respir | cough INT- D 1 | 1 | 0,02 |  | 0,09 | NO | x |
|  |  |  |  | mumps *EXT- P 2* |  |  |  |  | NO |  |
|  |  |  | genito | prostate *INT- E 3* | 0 |  |  |  | NO | x |
|  |  |  | skin | skin care complaints *EXT- B 2* | 0 |  |  |  | NO | x |
|  |  |  |  | inflammation *INT- D 4* |  |  |  |  | (6), (2), (26) |  |
|  |  |  | digest | stomach ache *INT- D 4* | 0 |  |  |  | (6), (26), (22) | x |
|  |  |  | GHUS | pain killer *INT- D 4* | 0 |  |  |  | NO | NO |
| *Polygonum cognatum* Meisn. *syn: Polygonum alpestre* C.A.Mey.  **(FP-SO 72)** | matitela (მატიტელა), cancar; *madımak, pencar, kuştabak* | 1 young leaves, decoction in water  2 young leaves, infusion in water  3 young leaves, vegetable dish  4 flowers, infusion in water  5 aerial parts, infusion in water | genito | kidney disease INT- D 2 | 3 | 0,20 | 0 | 0,03 | (9) | x |
|  |  |  |  | kidney pain INT- D 2 |  |  |  |  | NO |  |
|  |  |  | mu-sk | increase synovia INT- D 1 | 1 |  | 0 |  | NO | NO |
|  |  |  | endoc | diabetes INT- E 3 | 2 |  | 0 |  | (8), (18) | x |
|  |  |  | neuro | dizziness INT- D 2 | 1 |  | 0 |  | NO | NO |
|  |  |  | respir | cough INT- D 2 | 1 |  | 1 |  | (16) | x |
|  |  |  |  | throat ache *INT- D 4* |  |  |  |  | NO |  |
|  |  |  | skin | hair care complaints (hair wash)  *EXT- B 5* | 0 |  | 1 |  | NO | NO |
| *Polygonum hydropiper* L.  **(CK, SO 244*)** | tsalika (წალიკა) | 1 entire plant, decoction in water | cardio | hemorrhoids EXT- B 1 | 1 | 0,02 | 0 | 0,00 | NO | NO |
| *Rumex acetosella* L.  **(CK, SO 40)**  (CK, SO 1454, 1541) | kukumjava; *kuzi kulak, tırşo, kuzu kulağı* | 1 leaves, fresh | digest | indigestion *INT- E 1* | 1 | 0,02 | 1 | 0,01 | NO | x |
|  |  |  |  | liver disease INT- E 1 |  |  |  |  | NO |  |
| *Rumex crispus* L.  **(CK, SO 70)**  (CK, SO 654, 776) | hıreni, ghvalo, aveluk (Ավելուկ), shırenk-hıren;  *ğalo,  evelik,  tırşoberg,  poliyo,  tırşo* | 1 flowers, infusion in water  2 leaves, fresh  3 leaves, fresh mixed with honey  4 leaves, decoction in water  5 roots, decoction in water  6 roots, soup  7 roots, infusion in water  8 roots, mix with yogurt  9 roots, warmed on stove  10 seeds, infusion in water | genito | urinary disease **INT- D** *1,* 10 | 3 | 0,49 | 1 | 0,07 | NO | x |
|  |  |  | respir | throat ache EXT- P 2, 3 | 6 |  | 0 |  | (8) | x |
|  |  |  |  | influenza INT- D 5, 6 |  |  |  |  | NO |  |
|  |  |  | GHUS | fever *EXT- B 4* | 0 |  | 1 |  | (26) | x |
|  |  |  | digest | indigestion *INT- D 4* | 3 |  | 1 |  | NO | x |
|  |  |  |  | diarrhea INT- D 7 |  |  |  |  | (8), (26) |  |
|  |  |  |  | stomach ache INT- D 7 |  |  |  |  | NO |  |
|  |  |  |  | kidney pain EXT- P 2 |  |  |  |  | NO |  |
|  |  |  |  | gall disease INT- D 1 |  |  |  |  | NO |  |
|  |  |  | neuro | headache EXT- H 2 | 1 |  | 0 |  | NO | NO |
|  |  |  | mu-sk | bone and joint pain EXT- B 4, P 2;  *INT- D 5* | 4 |  | 1 |  | NO | NO |
|  |  |  |  | rheumatism INT- D 5 |  |  |  |  | NO |  |
|  |  |  |  | back pain EXT- B 5 |  |  |  |  | NO |  |
|  |  |  | skin | burn EXT- P 8, 9 | 2 |  |  |  | NO | x |
|  |  |  | cardio | hemorrhoids **INT-** *D 7;* EXT- B 5 | 3 |  | 1 |  | (16), (8) | x |
|  |  |  |  | high blood pressure INT- D 10 |  |  |  |  | NO |  |
| *Rumex scutatus* L.  (CK, SO 73) | *kuzu kulağı, tırşo* | 1 aerial parts, fresh | endoc | loss of appetite *INT- E 1* | 0 | 0,00 | 1 | 0,03 | (16) | x |
|  |  |  | cardio | hemorrhoids *INT- E 1* | 0 |  | 1 |  | NO | NO |
| RANUNCULACEAE |  |  |  |  |  |  |  |  |  |  |
| *Caltha palustris* L.  **(FP-SO 74)** | lilpar, kızı dikeni | 1 aerial parts, infusion in water | skin | hair care complaints (hair wash, dandruff) EXT- B 1 | 2 | 0,04 | 0 | 0,00 | NO | NO |
| *Ranunculus sp.* | tsalika (წალიკა) | 1 flowers, fresh | mu-sk | rheumatism EXT- P 1 | 1 | 0,02 | 0 | 0,00 | (4), (6), (16), (8), (20), (18), (19), (9) | x |
| ROSACEAE |  |  |  |  |  |  |  |  |  |  |
| *Alchemilla* spp.  **(CK, SO 33, 519)**  (CK, SO 1433) | manzhetska (Манжетка), marmichiya (მარმუჭი, marmuchi); *ayı pençesi aslan pençesi* | 1 aerial parts, infusion in water  2 aerial parts, decoction in water | genito | woman disease *INT- D 1, 2* | 1 | 0,04 | 2 | 0,04 | (12), (19) | x |
|  |  |  |  | menopausal complaint INT- D 1 |  |  |  |  | NO |  |
|  |  |  | digest | stomach ache INT- D 1 | 1 |  | 0 |  | (12), (20), (22) | x |
|  |  |  | respir | throat ache *INT- D 1* | 0 |  | 1 |  | NO | x |
| *Cotoneaster integerrimus Medik.* **(FP-SO 2)**  (CK, SO 1551) | kldevashla | 1 fruits, fresh | genito | women disease (infertility) INT- E 1 | 1 | 0,02 | 0 | 0,00 | NO | NO |
|  |  |  |  |  |  |  |  |  |  |  |
|  |  |  |  |  |  |  |  |  |  |  |
|  |  |  |  |  |  |  |  |  |  |  |
|  |  |  |  |  |  |  |  |  |  |  |
|  |  |  |  |  |  |  |  |  |  |  |
|  |  |  |  |  |  |  |  |  |  |  |
|  |  |  |  |  |  |  |  |  |  |  |
|  |  |  |  |  |  |  |  |  |  |  |
|  |  |  |  |  |  |  |  |  |  |  |
| *Crataegus azarolus L.* var. *pontica* (K.Koch) K.I.Chr.  syn: Crataegus  pontica K.Koch  **(FP-SO 13)**  (CK, SO 557*, 559*, 1770) | kurkantela (კურკანტელა); *datvi baba* | 1 fruits, fresh  2 fruits, sweet dish (pekmez) | cardio | heart disease INT- E 1 | 1 | 0,02 | 0 | 0,01 | NO | NO |
|  |  |  |  |  |  |  |  |  |  |  |
|  |  |  |  |  |  |  |  |  |  |  |
|  |  |  |  |  |  |  |  |  |  |  |
|  |  |  |  |  |  |  |  |  |  |  |
|  |  |  |  |  |  |  |  |  |  |  |
|  |  |  |  |  |  |  |  |  |  |  |
|  |  |  | endoc | diabetes *INT- E 2* | 0 |  | 1 |  | NO | NO |
| *Crataegus monogyna* Jacq.  *syn: Crataegus  kyrtostyla*Fingerh*.* **(CK, SO 452, 496)**  (CK, SO 1784) | kuneli (კუნელი), kirkat, kurkantela (კურკანტელა), knapi, vartan alucha (ալոճ, aloch); *kırkat, alıç* | 1 fruits, fresh  2 fruits, drink (juice)  3 fruits, sweet dishes (pekmez) mixed with Rosa sp. kuşburnu  4 fruits, sweet dish (compote) | genito | kidney disease INT- D 2 | 1 | 0,13 | 0 | 0,03 | NO | NO |
|  |  |  |  |  |  |  |  |  |  |  |
|  |  |  |  |  |  |  |  |  |  |  |
|  |  |  |  |  |  |  |  |  |  |  |
|  |  |  |  |  |  |  |  |  |  |  |
|  |  |  |  |  |  |  |  |  |  |  |
|  |  |  |  |  |  |  |  |  |  |  |
|  |  |  |  |  |  |  |  |  |  |  |
|  |  |  |  |  |  |  |  |  |  |  |
|  |  |  |  |  |  |  |  |  |  |  |
|  |  |  | cardio | heart disease INT- E 1, 4 | 4 |  | 0 |  | (24), (26), (23) | x |
|  |  |  | endoc | diabetes **INT- E 1**, *3* | 1 |  | 2 |  | NO | NO |
| *Crataegus orientalis* Pall. ex M.Bieb.  **(CK, SO 55*, 461)**  (FP-SO 3) | alucha, aloch, (ալոճ), vartan, kuneli (კუნელი), knapi; *ardıç* | 1 fruits, fresh  2 fruits, infusion in water | cardio | heart disease INT- E 1 | 2 | 0,04 | 0 | 0,01 | (8) | x |
|  |  |  |  |  |  |  |  |  |  |  |
|  |  |  |  |  |  |  |  |  |  |  |
|  |  |  |  |  |  |  |  |  |  |  |
|  |  |  |  |  |  |  |  |  |  |  |
|  |  |  |  |  |  |  |  |  |  |  |
|  |  |  |  |  |  |  |  |  |  |  |
|  |  |  |  |  |  |  |  |  |  |  |
|  |  |  | respir | asthma *INT- D 2* | 0 |  | 1 |  | (8) | x |
| *Crataegus pentagyna* Waldst. & Kit. ex Willd.  **(FP-SO 14)**  (CK, SO 1788) | shavi kuneli (შავი კუნელი), kuneli, kirkat, kriva; *tavşan kakası, kara kırkat* | 1 fruits, fresh  2 fruits, sweet dish (pekmez)  3 fruits, drink (juice)  4 fruits, decoction in water, mixed with Mentha sp. | cardio | heart disease INT- E 1, D 4 | 3 | 0,07 | 0 | 0,03 | (3), (15), (24), (25), (26) | x |
|  |  |  |  |  |  |  |  |  |  |  |
|  |  |  |  |  |  |  |  |  |  |  |
|  |  |  |  |  |  |  |  |  |  |  |
|  |  |  |  |  |  |  |  |  |  |  |
|  |  |  |  |  |  |  |  |  |  |  |
|  |  |  |  |  |  |  |  |  |  |  |
|  |  |  |  |  |  |  |  |  |  |  |
|  |  |  |  |  |  |  |  |  |  |  |
|  |  |  |  |  |  |  |  |  |  |  |
|  |  |  |  | vasodilator INT- D 3 |  |  |  |  | NO |  |
|  |  |  | endoc | diabetes *INT- E 1, 2* | 0 |  | 2 |  | NO | NO |
| *Crataegus pseudoheterophylla* Pojark.  **(CK, SO 20*)**  (FP-SO 4) | kuneli (კუნელი),tsiteli kuneli, litsvi (ლიცვი) | 1 fruits, fresh  2 fruits, drink (juice) | cardio | heart disease INT- E 1, 2 | 2 | 0,04 | 0 | 0,00 | NO | NO |
|  |  |  |  |  |  |  |  |  |  |  |
|  |  |  |  |  |  |  |  |  |  |  |
|  |  |  |  |  |  |  |  |  |  |  |
|  |  |  |  |  |  |  |  |  |  |  |
|  |  |  |  |  |  |  |  |  |  |  |
| *Crataegus rhipidophylla* Gand.  **(FP-SO 15)**  (FP-SO 5) | *ardıç, saphanelay* | 1 fruits, decoction in water  2 fruits, fresh | eye | eye diseases *INT- D 1* | 0 | 0,00 | 1 | 0,03 | NO | NO |
|  |  |  |  |  |  |  |  |  |  |  |
|  |  |  |  |  |  |  |  |  |  |  |
|  |  |  |  |  |  |  |  |  |  |  |
|  |  |  |  |  |  |  |  |  |  |  |
|  |  |  | skin | itching *INT- E 2* | 0 |  | 1 |  | NO | NO |
| *Filipendula* sp.  (FP-SO 75) | kapur (ქაფურა, kapura) | 1 entire plant, decoction in water  and mix with votka | mu-sk | bone and joint pain EXT- P 1 | 1 | 0,02 | 0 | 0,00 | NO | NO |
| *Fragaria viridis*Weston  **(CK, SO 153)** | mindris martskvi (მარწყვი,martsqvi), chighelek, marktsvi, sacsi | 1 fruits, fresh  2 leaves, infusion in water  3 leaves, decoction in water | GHUS | fever INT- E 1 | 1 | 0,09 | 0 | 0,00 | (3) | x |
|  |  |  | mu-sk | bone and joint pain EXT- B 3 | 1 |  | 0 |  | NO | NO |
|  |  |  | cardio | hemorrhoids INT- D 3 | 2 |  | 0 |  | NO | NO |
|  |  |  |  | high blood pressure INT- D 2 |  |  |  |  | NO |  |
| *Mespilus germanica* L.  **(FP-SO 16)**  (CK, SO 521*, 554*, 1050) | mushmula (Мушмула, mushmulá́); *muşmullah,* | 1 flowers, infusion in water  2 fruits, fresh  3 leaves, infusion in water, mixed with grape leaves | endoc | diabetes *INT- E 2* | 1 | 0,07 | 1 | 0,01 | (2), (21) | x |
|  |  |  |  |  |  |  |  |  |  |  |
|  |  |  |  |  |  |  |  |  |  |  |
|  |  |  |  |  |  |  |  |  |  |  |
|  |  |  |  |  |  |  |  |  |  |  |
|  |  |  |  |  |  |  |  |  |  |  |
|  |  |  |  |  |  |  |  |  |  |  |
|  |  |  |  |  |  |  |  |  |  |  |
|  |  |  |  | thyroid INT- E 2 |  |  |  |  | NO |  |
|  |  |  | cardio | heart disease INT- D 1 | 1 |  | 0 |  | (22) | x |
|  |  |  | mu-sk | bone and joint pain INT- D 3 | 1 |  | 0 |  | NO | NO |
| *Prunus padus* L.  *syn: Padus avium* Mill.  **(FP-SO 3)** | *karakiraz* | 1 fruits, fresh | GHUS | vitamin deficiency *INT- E 1* | 0 | 0,00 | 1 | 0,01 | NO | NO |
|  |  |  |  |  |  |  |  |  |  |  |
|  |  |  |  |  |  |  |  |  |  |  |
|  |  |  |  |  |  |  |  |  |  |  |
|  |  |  |  |  |  |  |  |  |  |  |
|  |  |  |  |  |  |  |  |  |  |  |
|  |  |  |  |  |  |  |  |  |  |  |
|  |  |  |  |  |  |  |  |  |  |  |
| *Prunus avium*(L.) L.  *syn: Cerasus avium (L.)* Moench  **(FP-SO 4)**  (CK, SO 402*, 33, 859, 1052, 1061, 1445, 1626, 1662) | bali (ბალი); *yabani kiraz, acı kiraz, tsare bali* | 1 fruits, fresh  2 fruits, decoction in water  3 leaves, decoction in water | genito | high menstrual bleeding INT- D 2 | 1 | 0,02 | 1 | 0,03 | NO | NO |
|  |  |  |  |  |  |  |  |  |  |  |
|  |  |  |  |  |  |  |  |  |  |  |
|  |  |  |  |  |  |  |  |  |  |  |
|  |  |  |  |  |  |  |  |  |  |  |
|  |  |  |  |  |  |  |  |  |  |  |
|  |  |  |  |  |  |  |  |  |  |  |
|  |  |  |  |  |  |  |  |  |  |  |
|  |  |  |  |  |  |  |  |  |  |  |
|  |  |  |  |  |  |  |  |  |  |  |
|  |  |  |  | vaginal discharge *INT- D 3* |  |  |  |  | NO |  |
|  |  |  | blood | anemia *INT- E 1* | 0 |  | 1 |  | NO | NO |
| *Prunus divaricata* Ledeb.  **(CK, SO 453, 459, 464)**  (CK, SO 35, 1539, 1646) | tkemali (ტყემალი), tkhemali, shavi tkhemali; *sarol, erik, temal* | 1 fruits, mixed with vodka and salt  2 fruits, sweet dish (pestil-kılapi)  3 fruits, mixed with honey  4 fruits, sweet dish (ezme)  5 roots, decoction in water | skin | wound EXT- P 1 | 1 | 0,13 | 0 | 0,04 | NO | x |
|  |  |  |  |  |  |  |  |  |  |  |
|  |  |  |  |  |  |  |  |  |  |  |
|  |  |  |  |  |  |  |  |  |  |  |
|  |  |  |  |  |  |  |  |  |  |  |
|  |  |  |  |  |  |  |  |  |  |  |
|  |  |  |  |  |  |  |  |  |  |  |
|  |  |  |  |  |  |  |  |  |  |  |
|  |  |  |  |  |  |  |  |  |  |  |
|  |  |  |  |  |  |  |  |  |  |  |
|  |  |  | mu-sk | bone and joint pain EXT- P 1 | 2 |  | 1 |  | NO | NO |
|  |  |  |  | sprain *EXT- P 3* |  |  |  |  | NO |  |
|  |  |  |  | back pain EXT- P 3 |  |  |  |  | NO |  |
|  |  |  | respir | throat ache INT- E 3 | 2 |  | 0 |  | NO | x |
|  |  |  |  | lung disease INT- E 3 |  |  |  |  | NO |  |
|  |  |  | cardio | haemorrhoids *INT- D 5* | 0 |  | 1 |  | NO | NO |
|  |  |  | endoc | diabetes *INT- E 4* | 0 |  | 1 |  | NO | NO |
|  |  |  | digest | stomach ache INT- E 2 | 1 |  | 0 |  | NO | NO |
| *Prunus laurocerasus* L. *syn: Laurocerasus  officinalis*M. Roem.  **(CK, SO 53, 83)**  (FP-SO 6) | tskavi (წყავი)*; karayemiş, zevağh* | 1 leaves, decoction in water  2 fruits, sweet dish (jam)  3 fruits, fresh | neuro | headache *INT- D 1* | 0 | 0,02 | 1 | 0,03 | (21) | x |
|  |  |  |  |  |  |  |  |  |  |  |
|  |  |  |  |  |  |  |  |  |  |  |
|  |  |  |  |  |  |  |  |  |  |  |
|  |  |  |  |  |  |  |  |  |  |  |
|  |  |  |  |  |  |  |  |  |  |  |
|  |  |  |  |  |  |  |  |  |  |  |
|  |  |  | blood | hematinic *INT- E 2* | 0 |  | 1 |  | (20) | x |
|  |  |  | cardio | haemorrhoids INT- E 3 | 1 |  | 0 |  | NO | x |
| *Pyrus communis*L.  **(CK, SO 22*, 450, 462)**  (CK, SO 445*, 55, 1036, 1054, 1632, 1642) | panta (პანტა) | 1 fruits, drink (vodka)  2 fruits, drink (juice) | digest | stomach ache INT- D 1 | 2 | 0,04 | 0 | 0,00 | (26) | x |
|  |  |  |  |  |  |  |  |  |  |  |
|  |  |  |  |  |  |  |  |  |  |  |
|  |  |  |  |  |  |  |  |  |  |  |
|  |  |  |  |  |  |  |  |  |  |  |
|  |  |  |  |  |  |  |  |  |  |  |
|  |  |  |  |  |  |  |  |  |  |  |
|  |  |  |  | constipation INT- D 2 |  |  |  |  | NO |  |
| *Rosa boissieri* Crép.  **(CK, SO 29*, 62*, 122*, 507)**  (FP-SO 7) | askili (ასკილი); *kuşburnu,güz askili, esgül* | 1 fruits, fresh  2 fruits, decoction in water  3 roots, decoction in water | genito | kidney disease INT- D 2 | 3 | 0,09 | 0 | 0,04 | (7) | x |
|  |  |  |  |  |  |  |  |  |  |  |
|  |  |  |  |  |  |  |  |  |  |  |
|  |  |  |  |  |  |  |  |  |  |  |
|  |  |  |  |  |  |  |  |  |  |  |
|  |  |  |  | incontinence urine INT- D 2 |  |  |  |  | NO |  |
|  |  |  | GHUS | vitamin deficiency INT- E 1 | 1 |  | 0 |  | NO | NO |
|  |  |  | digest | intestinal disease *INT- D 3* | 0 |  | 1 |  | (7) | x |
|  |  |  | respir | shortness of breath *INT- D 3* | 0 |  | 2 |  | NO | NO |
|  |  |  |  | cough *INT- D 2* |  |  |  |  | NO |  |
| *Rosa canina* L.  **(CK, SO 465)**  (CK, SO 1496, 1698, 1712, 1791) | askili (ასკილი), masur (մասուր), shipovnik (Шиповник),cherepidza*; kuşburnu,şilan, diken, ekali, yaz askili* | 1 fruits, infusion in water  2 fruits, decoction in water  3 roots, decoction in water  4 fruits, fresh  5 fruits, sweet dish (marmalade)  6 roots, infusion in water | genito | kidney disease INT- D 6 | 9 | 0,31 | 0 | 0,14 | (26) | x |
|  |  |  |  | prostate INT- D 1 |  |  |  |  | (26) |  |
|  |  |  |  | kidney pain INT- D 1 |  |  |  |  | NO |  |
|  |  |  |  | kidney stone INT- D 2 |  |  |  |  | (16) |  |
|  |  |  | cardio | hemorrhoids **INT- D** 1, *2, 4*, *E 5*;  *EXT- B 2* | 2 |  | 4 |  | (11), (13), (14), (22) | x |
|  |  |  |  |  |  |  |  |  |  |  |
|  |  |  | blood | cleansing blood INT- D 1 | 1 |  | 1 |  | NO | x |
|  |  |  |  | anemia *INT- E 4* |  |  |  |  | (26) |  |
|  |  |  | digest | stomach ache INT- D 1, 2 | 2 |  | 1 |  | (2), (14), (22), (26) | x |
|  |  |  |  | gall disease *EXT- B 2* |  |  |  |  | (26) |  |
|  |  |  | respir | cough *INT- D 2, 3* | 0 |  | 3 |  | (6), (10), (13), (14), (16), (21), (26) | x |
|  |  |  |  | influenza *INT- D 3* |  |  |  |  | (2), (5), (11), (17), (21) |  |
|  |  |  | GHUS | general disease *INT- D 3* | 0 |  | 1 |  | (26) | x |
| *Rosa hirtissima* Lonacz.  **(FP-SO 17)**  (FP-SO 8) | askili (ასკილი), masur (մասուր), grosni*; kuşburnu* | 1 fruits, decoction in water  2 fruits, infusion in water | genito | kidney disease INT- D 1 | 2 | 0,09 | 0 | 0,01 | NO | NO |
|  |  |  |  |  |  |  |  |  |  |  |
|  |  |  |  |  |  |  |  |  |  |  |
|  |  |  |  |  |  |  |  |  |  |  |
|  |  |  |  |  |  |  |  |  |  |  |
|  |  |  |  |  |  |  |  |  |  |  |
|  |  |  |  |  |  |  |  |  |  |  |
|  |  |  | GHUS | tiredness and weakness INT- D 2 | 1 |  | 0 |  | NO | NO |
|  |  |  | digest | stomach ache **INT- D 1** | 1 |  | 1 |  | NO | NO |
| *Rosa* *mollis* Sm.   **(CK, SO 264*, 280*)**  (FP-SO 9) | askili (ასკილი); *esgül* | 1 fruits, decoction in water  2 fruits, infusion in water, mixed with Helichrysum sp. (nego), Mentha sp. (pitna), Rosa sp. (askili) and chamomile (gvirila)  3 leaves, decoction in water | GHUS | general disease INT- D 1 | 2 | 0,11 | 0 | 0,01 | NO | NO |
|  |  |  |  | vitamin deficiency INT- D 1 |  |  |  |  | NO |  |
|  |  |  | cardio | heart disease INT- D 1 | 1 |  | 0 |  | NO | NO |
|  |  |  | genito | prostate *INT- D 1* | 0 |  | 1 |  | NO | NO |
|  |  |  | respir | bronchitis INT- D 2 | 1 |  | 0 |  | NO | NO |
|  |  |  | skin | antiseptic EXT- P 3 | 1 |  | 0 |  | NO | NO |
| *Rosa spinosissima* L.  syn: *Rosa  pimpinellifolia* L.   **(CK, SO 274)**  (CK, SO 615*, 24, 1019, 1024, 1529, 1669, 1711) | shavi askili (შავი ასკილი), masur (մասուր),chorni; *askil,eskül, esgül, kara eskül, kara kuşburnu, şilan, kuşburnu, siyah kuşburnu* | 1 fruits, fresh  2 fruits, infusion in water, 3 times per day  3 fruits, decoction in water  4 roots, decoction in water  5 roots, infusion in water | blood | anemia *INT- D 3* | 0 | 0,09 | 1 | 0,32 | NO | NO |
|  |  |  | GHUS | vitamin deficiency INT- E 1 | 2 |  | 2 |  | NO | NO |
|  |  |  |  | general disease *INT- D 4* |  |  |  |  | NO |  |
|  |  |  |  | tiredness and weakness INT- D 2 |  |  |  |  | NO |  |
|  |  |  |  |  |  |  |  |  |  |  |
|  |  |  | cardio | blood pressure INT- D 2 | 2 |  | 7 |  | NO | x |
|  |  |  |  | haemorrhoids *EXT- B 3, 4; INT- D 3, 4* |  |  |  |  | (7), (8), (9), (16), (18) |  |
|  |  |  |  |  |  |  |  |  |  |  |
|  |  |  |  | hearth disease INT- E 1 |  |  |  |  | (16) |  |
|  |  |  | respir | influenza *INT- D 4* | 0 |  | 7 |  | (6), (5) | x |
|  |  |  |  | shortness of breath *INT- D 4* |  |  |  |  | NO |  |
|  |  |  |  | bronchitis *INT- D 4* |  |  |  |  | NO |  |
|  |  |  |  | cough *INT- D 3, 4* |  |  |  |  | (8), (16) |  |
|  |  |  | neuro | headache *INT- D 2* | 0 |  | 2 |  | NO | NO |
|  |  |  |  | dizziness *INT- D 5* |  |  |  |  | NO |  |
|  |  |  | digest | stomach ache *INT- D 2, 3* | 0 |  | 4 |  | (6), (8), (16) | x |
|  |  |  |  | intestinal disease *INT- D 4* |  |  |  |  | (7), (8) |  |
|  |  |  |  | clean intestine *INT- D 4* |  |  |  |  | NO |  |
|  |  |  | genito | prostate *INT- D 3* | 0 |  | 1 |  | NO | NO |
| *Rosa villosa* L.  **(CK, SO 451, 497)**  (CK, SO 1118, 1528, 1594, 1769) | *kuşburnu,dıbılga* | 1 fruits, decoction in water  2 roots, infusion in water | digest | stomach ache *INT- D 1* | 0 | 0,00 | 1 | 0,03 | NO | NO |
|  |  |  |  |  |  |  |  |  |  |  |
|  |  |  |  |  |  |  |  |  |  |  |
|  |  |  |  |  |  |  |  |  |  |  |
|  |  |  | respir | cough *INT- D 2* | 0 |  | 1 |  | NO | NO |
| *Rubus caesius* L.  **(CK, SO 90)**  (FP-SO 10) | makvali (მაყვალი, mak'vali), bardi | 1 roots, decoction in water  2 leaves, infusion in water | genito | kidney disease INT- D 1 | 1 | 0,07 | 0 | 0,00 | NO | NO |
|  |  |  |  |  |  |  |  |  |  |  |
|  |  |  |  |  |  |  |  |  |  |  |
|  |  |  |  |  |  |  |  |  |  |  |
|  |  |  |  |  |  |  |  |  |  |  |
|  |  |  | digest | kidney disease INT- D 1 | 1 |  | 0 |  | NO | x |
|  |  |  | mu-sk | bone and joint pain INT- D 2 | 1 |  | 0 |  | NO | NO |
| *Rubus caucasicus* Focke  **(CK, SO 516)**  (CK, SO 656, 1432, 1505) | makvali (მაყვალი) | 1 fruits, fresh  2 leaves, decoction in water, mixed with *Vaccinium* sp. and *Rubus* *idaeus* leaves | respir | cold INT- E 1 | 1 | 0,04 | 0 | 0,00 | NO | NO |
|  |  |  |  |  |  |  |  |  |  |  |
|  |  |  |  |  |  |  |  |  |  |  |
|  |  |  |  |  |  |  |  |  |  |  |
|  |  |  |  |  |  |  |  |  |  |  |
|  |  |  |  |  |  |  |  |  |  |  |
|  |  |  |  |  |  |  |  |  |  |  |
|  |  |  |  |  |  |  |  |  |  |  |
|  |  |  | skin | eczema INT- D 2 | 1 |  | 0 |  | NO | NO |
|  |  |  |  |  |  |  |  |  | NO |  |
| *Rubus idaeus* L.  **(CK, SO 168*, 94, 454)**  (CK, SO 348*, 408*, 417*, 468*, 601*, 812, 1021, 1504, 1530, 1675) | malina (Малина), makvali (მაყვალი), smorodina (смородина), jolo | 1 young leaves, infusion in water  2 leaves, infusion in water  3 fruits, sweet dish (jam), 1:1 sugar: fruit, boil 5 min., one table spoon  4 fruits, sweet dish (compote)  5 leaves, decoction in water, mixed with Vaccinium sp. Leaves | respir | throat ache INT- D 1 | 3 | 0,16 | 0 | 0,00 | NO | x |
|  |  |  |  |  |  |  |  |  |  |  |
|  |  |  |  |  |  |  |  |  |  |  |
|  |  |  |  |  |  |  |  |  |  |  |
|  |  |  |  |  |  |  |  |  |  |  |
|  |  |  |  |  |  |  |  |  |  |  |
|  |  |  |  |  |  |  |  |  |  |  |
|  |  |  |  |  |  |  |  |  |  |  |
|  |  |  |  |  |  |  |  |  |  |  |
|  |  |  |  |  |  |  |  |  |  |  |
|  |  |  |  |  |  |  |  |  |  |  |
|  |  |  |  |  |  |  |  |  |  |  |
|  |  |  |  |  |  |  |  |  |  |  |
|  |  |  |  |  |  |  |  |  |  |  |
|  |  |  |  |  |  |  |  |  |  |  |
|  |  |  |  |  |  |  |  |  |  |  |
|  |  |  |  |  |  |  |  |  |  |  |
|  |  |  |  |  |  |  |  |  |  |  |
|  |  |  |  |  |  |  |  |  |  |  |
|  |  |  |  |  |  |  |  |  |  |  |
|  |  |  |  | influenza INT- D 2 |  |  |  |  | (25) |  |
|  |  |  | GHUS | cleaning organs INT- D 2 | 2 |  | 0 |  | NO | x |
|  |  |  |  | fever INT- E 4 |  |  |  |  | (3) |  |
|  |  |  | cardio | heart disease INT- E 3 | 1 |  | 0 |  | NO | NO |
|  |  |  | skin | eczema INT- D 5 | 1 |  | 0 |  | (10) | x |
|  |  |  |  |  |  |  |  |  |  |  |
| *Sorbus aucaparia* L.  syn: *Sorbus caucasigen*a Kom. ex Gatsch.  **(CK, SO 75, 456)**  (CK, SO 390*, 421*, 447*, 594*, 626*, 1459, 1591, 1660) | chknavi, (ჭნავი, ch’navi), chakvela (ჭვახა, ch’vakha)*; çinav, cinav, çinav, dolık, cahole* | 1 fruits, fresh  2 fruits, infusion in water | endoc | diabetes **INT- E 1** | 1 | 0,04 | 5 | 0,11 | (26) | x |
|  |  |  |  |  |  |  |  |  |  |  |
|  |  |  |  |  |  |  |  |  |  |  |
|  |  |  |  |  |  |  |  |  |  |  |
|  |  |  |  |  |  |  |  |  |  |  |
|  |  |  |  |  |  |  |  |  |  |  |
|  |  |  |  |  |  |  |  |  |  |  |
|  |  |  |  |  |  |  |  |  |  |  |
|  |  |  |  |  |  |  |  |  |  |  |
|  |  |  |  |  |  |  |  |  |  |  |
|  |  |  |  |  |  |  |  |  |  |  |
|  |  |  | respir | asthma *INT- E 1* | 0 |  | 1 |  | NO | x |
|  |  |  | digest | stomach ache *INT- E 1* | 0 |  | 1 |  | NO | x |
|  |  |  | cardio | haemorrhoids INT- E 1 | 1 |  | 1 |  | (26) | x |
|  |  |  |  | high blood pressure *INT- D 2* |  |  |  |  | (24), (25), (26) |  |
|  |  |  |  |  |  |  |  |  |  |  |
| SALICACEAE |  |  |  |  |  |  |  |  |  |  |
| *Salix caprea* L.  **(CK, SO 61*, 192*, 212*, 82, 98, 506, 522)**  (CK, SO 506*, 522*, 1034) | mdgnali (მდგნალი) | 1 flowers, infusion in water | eye | eye diseases EXT- P 1 | 1 | 0,02 | 0 | 0,00 | NO | NO |
|  |  |  |  |  |  |  |  |  |  |  |
|  |  |  |  |  |  |  |  |  |  |  |
|  |  |  |  |  |  |  |  |  |  |  |
|  |  |  |  |  |  |  |  |  |  |  |
|  |  |  |  |  |  |  |  |  |  |  |
|  |  |  |  |  |  |  |  |  |  |  |
|  |  |  |  |  |  |  |  |  |  |  |
|  |  |  |  |  |  |  |  |  |  |  |
|  |  |  |  |  |  |  |  |  |  |  |
|  |  |  |  |  |  |  |  |  |  |  |
|  |  |  |  |  |  |  |  |  |  |  |
|  |  |  |  |  |  |  |  |  |  |  |
|  |  |  |  |  |  |  |  |  |  |  |
|  |  |  |  |  |  |  |  |  |  |  |
| *Salix* sp.  **(CK, SO 208*)**  (CK, SO 1405, 1792) | latso ureni (Ուռենի, urreni), tsitsera; *söğüt* | 1 leaves, infusion in water  2 young branches, warmed on stove  3 branches, pray on *Salix* sp. branches and make a notch on it as the number of warts you have, when it fall down from roof, wart disappear | GHUS | pain killer INT- D 1 | 1 | 0,04 | 0 | 0,01 | (4) | x |
|  |  |  |  |  |  |  |  |  |  |  |
|  |  |  |  |  |  |  |  |  |  |  |
|  |  |  |  |  |  |  |  |  |  |  |
|  |  |  |  |  |  |  |  |  |  |  |
|  |  |  |  |  |  |  |  |  |  |  |
|  |  |  |  |  |  |  |  |  |  |  |
|  |  |  |  |  |  |  |  |  |  |  |
|  |  |  |  |  |  |  |  |  |  |  |
|  |  |  |  |  |  |  |  |  |  |  |
|  |  |  |  |  |  |  |  |  |  |  |
|  |  |  |  |  |  |  |  |  |  |  |
|  |  |  | mu-sk | sprain EXT- P 2 | 1 |  | 0 |  | NO | x |
|  |  |  | skin | wart *EXT- RT 3* | 0 |  | 1 |  | NO | x |
| SANTALACEAE |  |  |  |  |  |  |  |  |  |  |
| *Viscum album* L.  **(FP-SO 5)** | pitri (ფითრი) pintri*; pintri* | 1 aerial parts, infusion in water  2 leaves, infusion in water  3 fruits, macerated | genito | abortion *INT- D 1* | 1 | 0,07 | 2 | 0,07 | NO | NO |
|  |  |  |  |  |  |  |  |  |  |  |
|  |  |  |  |  |  |  |  |  |  |  |
|  |  |  |  | urinary disease INT- D 2 |  |  |  |  | NO |  |
|  |  |  | skin | wart (meçeçi) EXT- P 3 | 2 |  | 0 |  | NO | NO |
|  |  |  | respir | asthma *INT- D 2* | 0 |  | 2 |  | (20), (21), (22) | x |
|  |  |  | endoc | diabetes *INT- D 2* | 0 |  | 1 |  | NO | x |
| SCROPHULARIACEAE |  |  |  |  |  |  |  |  |  |  |
| *Verbascum* spp.  (CK, SO 1547, 1592) | *öküz kuyruğu, öküz pörçiğü, mejerik,  sığır kuyruğu, marjerk,  mavuljak* | 1 aerial parts with flowers, infusion in water  2 aerial parts with flowers, decoction in water  3 aerial parts with flowers, fresh  4 entire plant, infusion in water  5 roots, infusion in water  6 leaves, fresh | digest | stomach ache INT- D 4 | 1 | 0,02 | 0 | 0,07 | NO | x |
|  |  |  | cardio | hemorrhoids *INT- D 5; EXT- B 1* | 0 |  | 2 |  | (25), (17), (9) | x |
|  |  |  | skin | antifungal *EXT- B 2* | 0 |  | 3 |  | (16) | x |
|  |  |  |  | wound *EXT- P 3, 6* |  |  |  |  | NO |  |
| SOLANACEAE |  |  |  |  |  |  |  |  |  |  |
| *Hyoscyamus niger* L.  **(FP-SO 77)**  (FP-SO 76) | lentsopa (ლენცოფა), aghınshpank*; batbatik,  hırbeng, badbad, patpat,  patpada,  deli patpat* | 1 seeds, decoction in water  2 leaves, decoction in water  3 seeds, burned  4 seeds, fresh | digest | tooth inflammation EXT- G 2; **INT- V 3** | 6 | 0,13 | 12 | 0,18 | NO | x |
|  |  |  |  | toothache **INT- V 1, 3,** *C 4* |  |  |  |  | (4), (13), (25), (9), (16), (18), (14) |  |
|  |  |  | mu-sk | bone and joint pain *EXT- B 2* | 0 |  | 1 |  | NO | x |
| *Physalis alkekengi* L.   (FP-SO 78) | *altınçilek* | 1 fruits, fresh | genito | prostate *INT- E 1* | 0 | 0,00 | 1 | 0,01 | NO | x |
| TAXACEAE |  |  |  |  |  |  |  |  |  |  |
| *Taxus baccata* L. | mada, chancholo | 1 fruits, fresh | GHUS | general disease INT- E 1 | 1 | 0,02 | 0 | 0,00 | NO | NO |
|  |  |  |  |  |  |  |  |  |  |  |
|  |  |  |  |  |  |  |  |  |  |  |
| THYMELAEACEAE |  |  |  |  |  |  |  |  |  |  |
| *Daphne mezereum* L.  **(FP-SO 18)**  (CK, SO 608*, 1259, 1476) | *mayasır* | 1 branches with leaves, decoction in water | cardio | hemorrhoids *EXT- B 1* | 0 | 0,00 | 1 | 0,01 | NO | NO |
|  |  |  |  |  |  |  |  |  |  |  |
|  |  |  |  |  |  |  |  |  |  |  |
|  |  |  |  |  |  |  |  |  |  |  |
|  |  |  |  |  |  |  |  |  |  |  |
| URTICACEAE |  |  |  |  |  |  |  |  |  |  |
| *Urtica dioica* L.  **(CK, SO 510)**  (CK, SO 1526) | krapiva (крапива), chinchari (ჭინჭარი), chinchar, yeghinç (Եղինջ, yeghinj), kecan, ısırgan, ğaci, cinçris; *gez gezik, ısırgan, çinçar, geznik, gevgez, kevkesk, yabani ısırgan* | 1 aerial parts, fresh  2 aerial parts, infusion in alcohol at dark place  3 aerial parts, decoction in water  4 aerial parts, infusion in water  5 aerial parts, juice  6 aerial prts, decoction in water, mixed with chammomile  7 entire plant, infusion in water  8 entire plant, decoction in water  9 roots, mix with outer bark *mukha,* infusion in water  10 roots, decoction in water, smashed  11 seeds, fresh, mix with honey  12 seeds, decoction in water  13 leaves, infusion in water  14 leaves, decoction in water  15 leaves, fresh | blood | cleansing blood INT- D 4, 5 | 3 | 1,38 | 1 | 0,73 | (26) | x |
|  |  |  |  | anemia *INT- D 7,* E 15 |  |  |  |  | (1), (26) |  |
|  |  |  | endoc | diabetes *EXT- B 3* | 0 |  | 1 |  | (6), (16), (8),  (17), (9), (22), (15), (26) | x |
|  |  |  | genito | kidney stone *INT- D 4* | 1 |  | 1 |  | (26) | x |
|  |  |  |  | kidney disease INT- D 13 |  |  |  |  | (19), (9), (22), (26) |  |
|  |  |  | mu-sk | bone and joint pain INT- D 3;  *EXT- B 3, 10,* **P 1,** *11* | 21 |  | 23 |  | (24), (26) | x |
|  |  |  |  | cramp EXT- B 3 |  |  |  |  | NO |  |
|  |  |  |  | rheumatism **EXT- B 3, 8, 14***, 6,* **P 1,** 15; ***INT****- E 1,* **D** 2, *12,* 13*, 14* |  |  |  |  | (13), (5), (2), (26)(8), (20), (17), (18), (14), (19), (9), (22) |  |
|  |  |  |  | back pain *EXT- B 13* |  |  |  |  | (20) |  |
|  |  |  | neuro | headache *EXT- H 1* | 1 |  | 1 |  | (26) | x |
|  |  |  |  | increase memory INT- D 13 |  |  |  |  | NO |  |
|  |  |  | respir | sinusitis *INT- V 4* | 3 |  | 5 |  | NO | x |
|  |  |  |  | influenza *INT- D 6* |  |  |  |  | NO |  |
|  |  |  |  | bronchitis *INT- E 11, D 12* |  |  |  |  | (6), (12), (21) |  |
|  |  |  |  | lung disease INT- E 11 |  |  |  |  | (19) |  |
|  |  |  |  | cough INT- D 13 |  |  |  |  | (13), (6), (2),  (21) |  |
|  |  |  | skin | hair care complaints (hair wash, loss, dandruff and growth) **EXT- B 3, 8,** 9, 13, 14, P 1 | 27 |  | 19 |  | (8), (11), (22),  (26) | x |
|  |  |  |  | blister EXT- B 3 |  |  |  |  | NO |  |
|  |  |  |  | itching *EXT- B 3* |  |  |  |  | NO |  |
|  |  |  |  | wound *EXT- P 10, 15; INT- D 13* |  |  |  |  | (24), (19), (22), (26) |  |
|  |  |  | cardio | blood circulation EXT- BS 1 | 3 |  | 0 |  | (26) | x |
|  |  |  |  | high blood pressure *INT- D 8* |  |  |  |  | (6), (25), (16),  (26) |  |
|  |  |  |  | hemorrhoids EXT- B 13 |  |  |  |  | (16), (8), (11),  (22), (26) |  |
|  |  |  | digest | ulcer *INT- E 11* | 3 |  | 2 |  | (6), (8), (19), (26) | x |
|  |  |  |  | constipation INT- E 15 |  |  |  |  | (26) |  |
|  |  |  |  | diarrhea INT- D 13 |  |  |  |  | (26) |  |
|  |  |  |  | stomach ache INT- D 13 |  |  |  |  | (13), (20),  (17), (9), (26) |  |
|  |  |  |  | clean intestine *INT- E 11* |  |  |  |  | NO |  |
|  |  |  | GHUS | good for health *INT- E 11* | 0 |  | 1 |  | NO | x |

^a^ “CK, SO (number)”: Voucher number of species collected by Ceren Kazancı and Soner Oruç. ^“^FP-SO (number)”: Field photo number of the specimen by Soner Oruç. **Bold** numbers indicate specimens from Georgia, the others from Turkey. * represents specimens from 2017 fieldwork; the others from 2018.

^b^ Recorded local names of species in both countries during our fieldworks. Names written in *Italics* are from Turkey, the rest are from Georgia. When exactly the same local/common names were recorded, the correct spelling was shown in parenthesis with Georgian, Russian, Armenian alphabet.

^c^ Each different number (1,2,3…) indicates a single preparation of a remedy.

^d^ digest: Digestive, respir: Respiratory, cardio: Cardiovascular, skin: Skin, endoc: Endocrine, GHUS: General health and unspecified, genito: Genitourinary, mu-sk: Muscle-Skeletal, blood: Blood, eye: Eye; ear: Ear, neuro: Neurological, psych: Psychological.

^e^ Ailments written in **bold** are shared reports between participants in both country. The rest of the reports are either from Georgia or from Turkey depending on their application methods. Applications written in *Italics* are associated with the knowledge from Turkey; **Bolds** are for both countries, the rest are from Georgia. Each number (1,2,3…) at the end of applications match with the process number of the remedy (see in above “^c^”). INT: Internally (D: drink, E: eaten, C: chew, V: inhalation of vapor, RT: ritual, T:tamp); EXT: Externally (P: plaster, G: gargle, B: bath, EM: embrocation, H: headbands, BS: beating of skin, F: fumigate, RT: ritual).

^f^ The numbers matches with references below, indicates that related medicinal emic use of the plant species are same in the cited references. “NO” indicates that “not any similar report have been specified” in the cited references.

^g^ Cross sign indicates similar reports are given in the cited references based on medicinal category. “NO” indicates that “no use is reported in that medicinal category” in the cited references.

Numbers and its corresponding references: **(1) 14; (2) 21; (3) 25; (4) 19; (5) 23; (6) 69; (7) 81; (8) 67; (9) 68; (10) 75; (11) 76; (12) 70; (13) 20; (14) 71; (15) 17; (16) 79; (17) 85; (18) 72; (19) 73; (20) 77; (21) 74; (22) 78; (23) 86; (24) 12; (25) 16; (26) 18**
